# Paternal hypercholesterolemia elicits sex-specific exacerbation of atherosclerosis in offspring

**DOI:** 10.1172/jci.insight.179291

**Published:** 2024-09-10

**Authors:** Rebecca Hernandez, Xiuchun Li, Junchao Shi, Tejasvi R. Dave, Tong Zhou, Qi Chen, Changcheng Zhou

**Affiliations:** 1 Division of Biomedical Sciences, School of Medicine, University of California, Riverside, California, USA.; 2 Molecular Medicine Program, Department of Human Genetics, and; 3 Division of Urology, Department of Surgery, School of Medicine, University of Utah, Salt Lake City, Utah, USA.; 4 Department of Physiology and Cell Biology, University of Nevada, Reno School of Medicine, Reno, Nevada, USA.

**Keywords:** Cell biology, Vascular biology, Atherosclerosis, Cardiovascular disease, Epigenetics

## Abstract

Emerging studies suggest that various parental exposures affect offspring cardiovascular health, yet the specific mechanisms, particularly the influence of paternal cardiovascular disease (CVD) risk factors on offspring cardiovascular health, remain elusive. The present study explores how paternal hypercholesterolemia affects offspring atherosclerosis development using the LDL receptor-deficient (LDLR^–/–^) mouse model. We found that paternal high-cholesterol diet feeding led to significantly increased atherosclerosis in F1 female, but not male, LDLR^–/–^ offspring. Transcriptomic analysis highlighted that paternal hypercholesterolemia stimulated proatherogenic genes, including *Ccn1* and *Ccn2*, in the intima of female offspring. Sperm small noncoding RNAs (sncRNAs), particularly transfer RNA–derived (tRNA-derived) small RNAs (tsRNAs) and rRNA-derived small RNAs (rsRNAs), contribute to the intergenerational transmission of paternally acquired metabolic phenotypes. Using a newly developed PANDORA-Seq method, we identified that high-cholesterol feeding elicited changes in sperm tsRNA/rsRNA profiles that were undetectable by traditional RNA-Seq, and these altered sperm sncRNAs were potentially key factors mediating paternal hypercholesterolemia-elicited atherogenesis in offspring. Interestingly, high-cholesterol feeding altered sncRNA biogenesis–related gene expression in the epididymis but not testis of LDLR^–/–^ sires; this may have led to the modified sperm sncRNA landscape. Our results underscore the sex-specific intergenerational effect of paternal hypercholesterolemia on offspring cardiovascular health and contribute to the understanding of chronic disease etiology originating from parental exposures.

## Abstract 2

High-cholesterol diet feeding in male mice increases cardiovascular disease risk in their offspring, highlighting the influence of unhealthy diet exposures on next generation’s cardiovascular health.

## Graphical abstract

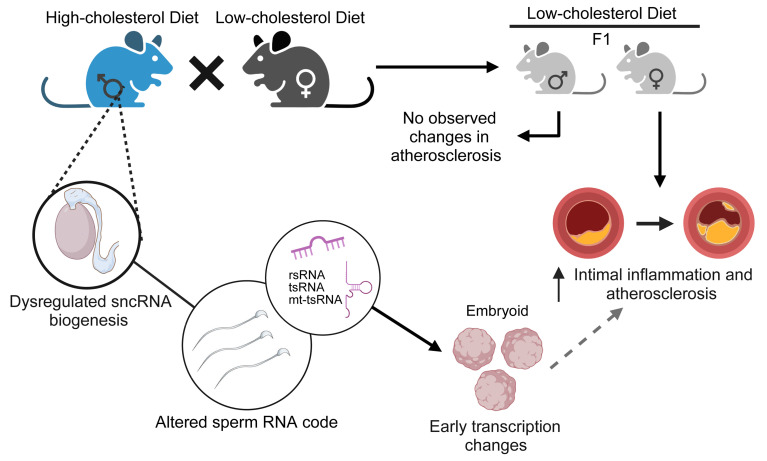

## Introduction

Atherosclerosis is a complex disease characterized by the accumulation of cholesterol in large arteries, leading to plaque development in the intimal layer of the artery (1–3). Despite major advances in diagnoses and treatments, atherosclerotic cardiovascular disease (CVD) is still the leading cause of mortality and morbidity worldwide (4, 5). While many modifiable risk factors such as smoking, diet, and obesity have been well established to contribute to atherosclerosis, there are also genetic and epigenetic factors that can increase the risk of developing atherosclerotic CVD (6), which may reduce the effectiveness of lifestyle interventions and current therapeutic interventions. Therefore, it is critical to identify these genetic and epigenetic factors and to understand their mechanisms in atherosclerosis development to enhance patient preventative care through early detection methods.

In addition to the well-known traditional risk factors, strong evidence has suggested that parental environmental influences can affect the health of future generations (7–19). Several large-scale clinical studies — including the Framingham Heart Study, the Physicians’ Health study, and the Women’s Health Study — have demonstrated the effect of parental CVD on offspring cardiometabolic health and have revealed that offspring of parents with a history of early-onset CVD have a higher risk of developing CVD, even after adjusting for other risk factors (7, 9, 20). A few other human and animal studies have also demonstrated that maternal or in utero exposure to certain CVD risk factors can elicit increased CVD risk in offspring. For example, hypercholesterolemia in mothers during pregnancy can significantly increase the number and size of fatty streaks in fetal aortas (21, 22). The Fate of Early Lesions in Children (FELIC) study demonstrated that aortic lesions in children of hypercholesterolemic mothers progressed much faster than that of children of normocholesterolemic mothers (23, 24). Animal studies have also demonstrated that maternal or perinatal hypercholesterolemia can cause increased cardiovascular dysfunction or atherosclerosis development in the offspring (25–27). We also reported that maternal exposure to an atherogenic endocrine disrupting chemical (EDC), bisphenol A, led to exacerbated atherosclerosis in the adult offspring in mice (28).

While most studies have focused on the effects of maternal factors on offspring health, emerging evidence suggests that paternal exposures can also affect offspring’s cardiometabolic heath (12–19, 29–32). For example, we previously demonstrated that paternal high-fat diet (HFD) feeding in mice can induce metabolic disorders in the offspring (13, 14, 29). Several other studies also found that exposure of male rats or mice to HFD can lead to increased diabetic phenotypes or metabolic disfunctions in offspring (12, 32). Paternal exposure to low-protein diets caused impaired vascular function and metabolic disorders in mouse offspring (16–19). In addition to dietary exposure, we and others reported that paternal exposure to a range of environmental toxicants can have inter- and transgenerational adverse effects on the metabolic health of their offspring (33–36). While various sperm epigenetic factors have been proposed to contribute to intergenerational inheritance of environment-induced phenotypes in mammals (37), sperm small noncoding RNAs (sncRNAs), especially transfer RNA–derived (tRNA-derived) small RNAs (tsRNAs) and rRNA-derived small RNAs (rsRNAs), can significantly contribute to the intergenerational transmission of paternally acquired metabolic phenotypes (29, 32, 38, 39).

While previous studies have suggested that paternal factors affect offspring metabolic health, there are no animal studies investigating the effect of paternal exposures to unhealthy diets on atherosclerosis development of offspring. In the current study, we investigated the effect of paternal hypercholesterolemia on offspring atherosclerosis development in LDL receptor–deficient (LDLR^–/–^) mice. We reported here that paternal high-cholesterol diet (HCD) feeding led to significantly increased atherosclerosis in F1 female LDLR^–/–^ offspring. We then used an innovative small RNA-Seq method, PANDORA-Seq (40, 41), to unveil the paternal hypercholesterolemia-elicited sperm sncRNA changes that may contribute to paternally acquired atherosclerosis in offspring.

## Results

### Male LDLR^–/–^ mice fed a low-fat, HCD develop severe hypercholesterolemia and atherosclerosis.

**Figure 1:**
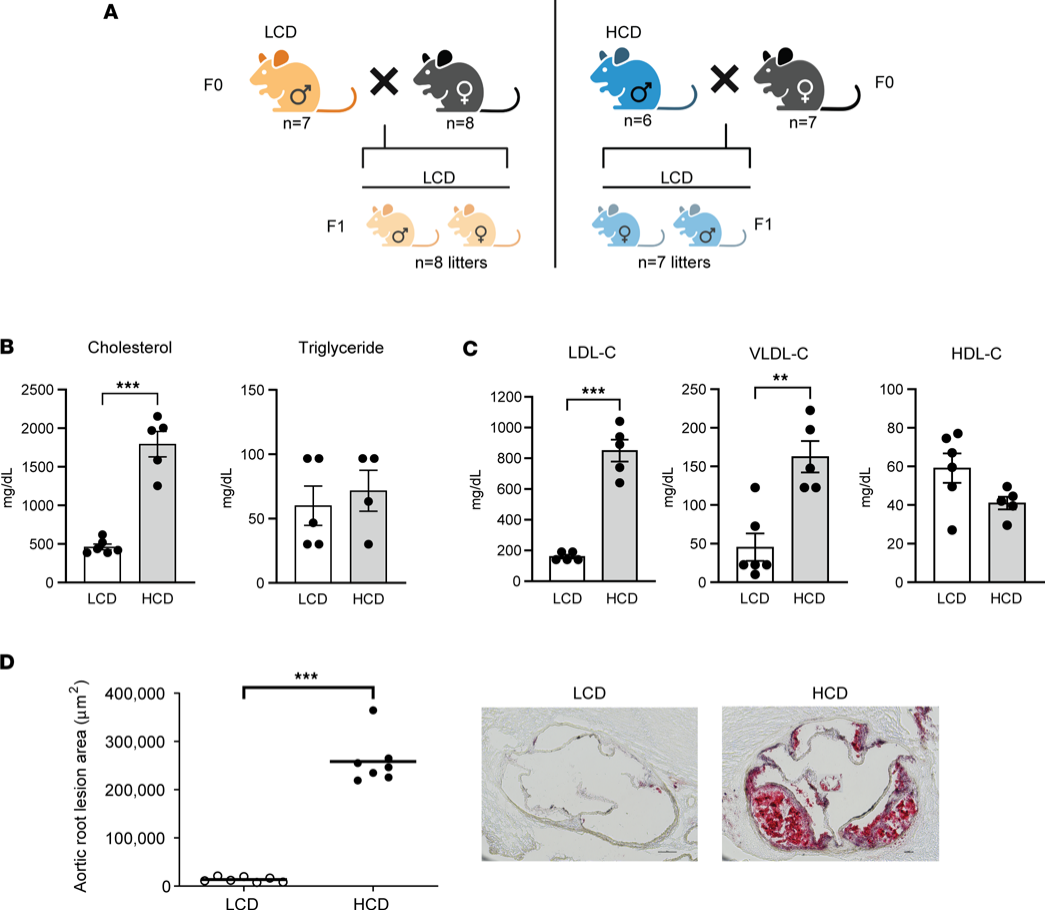
Male LDL receptor–deficient mice fed a low-fat, high-cholesterol diet develop severe hypercholesterolemia-mediated atherosclerosis. Three-week-old male LDLR^–/–^ mice were fed a low-cholesterol diet (LCD, 0.02% cholesterol) or high-cholesterol diet (HCD, 0.5% cholesterol) for 8 weeks before mating with female LDLR^–/–^ mice. The F1 offspring were weaned at 3 weeks old and were fed an LCD for 16 weeks. (**A**) Schematic representation of experimental design and generation of F1 offspring. (**B**) Serum total cholesterol and triglyceride levels were measured (*n* = 4–6, ***P < 0.001, 2-sample, 2-tailed Student’s *t* test). (**C**) Lipoprotein fractions (VLDL-C, LDL-C, and HDL-C) were isolated from serum, and the cholesterol levels of each fraction were measured (*n* = 5–6, **P < 0.01, ***P < 0.001; 2-sample, 2-tailed Student’s *t* test). (**D**) Quantitative analysis of the lesion area in the aortic root of LCD- and HCD-fed LDLR^–/–^ mice (*n* = 7, ***P < 0.05, 2-sample, 2-tailed Student’s *t* test). Representative images are shown to the right. VLDL-C, very low-density lipoprotein cholesterol; LDL-C, low density lipoprotein cholesterol; HDL-C, high density lipoprotein cholesterol. All data are plotted as mean ± SEM. Scale bar: 100 μm.

To investigate the effects of paternal hypercholesterolemia on offspring cardiometabolic health, 3-week-old male LDLR^–/–^ mice were fed a low-fat AIN76 diet (4.3% fat) containing either 0.02% or 0.5% cholesterol for 8 weeks before mating with age-matched control LDLR^–/–^ female mice (42, 43) (Figure 1A). The HCD containing 0.5% cholesterol has been previously used to promote severe hypercholesterolemia or atherosclerosis in mice without inducing obesity and other metabolic disorders (42–46). Pregnant female LDLR^–/–^ mice were housed separately and fed a low-cholesterol diet (LCD) containing 0.02% cholesterol after the vaginal plug was identified (E0.5).

Consistent with our previous studies (43), LDLR^–/–^ mice fed a HCD had similar body weight (BW) or growth curve and lean/fat mass as that of LCD-fed LDLR^–/–^ mice (Supplemental Figure 1, A and B; supplemental material available online with this article; https://doi.org/10.1172/jci.insight.179291DS1). As expected, HCD feeding led to elevated serum total cholesterol levels without affecting triglyceride levels (Figure 1B). Lipoprotein fraction analyses showed that HCD-fed mice had significantly elevated atherogenic LDL and VLDL cholesterol levels but had similar HDL cholesterol levels as compared with LCD-fed mice (Figure 1C). Atherosclerotic lesions at the aortic root were then measured in those mice. As expected, HCD feeding led to larger atherosclerotic lesion sizes at the aortic root of LDLR^–/–^ mice as compared with mice fed the LCD (Figure 1D). Thus, LDLR^–/–^ mice fed this low-fat HCD developed severe hypercholesterolemia and large atherosclerotic lesions without increased obesity.

### Paternal HCD feeding does not affect serum lipid levels and metabolic phenotypes in F1 LDLR^–/–^ offspring.

**Figure 2:**
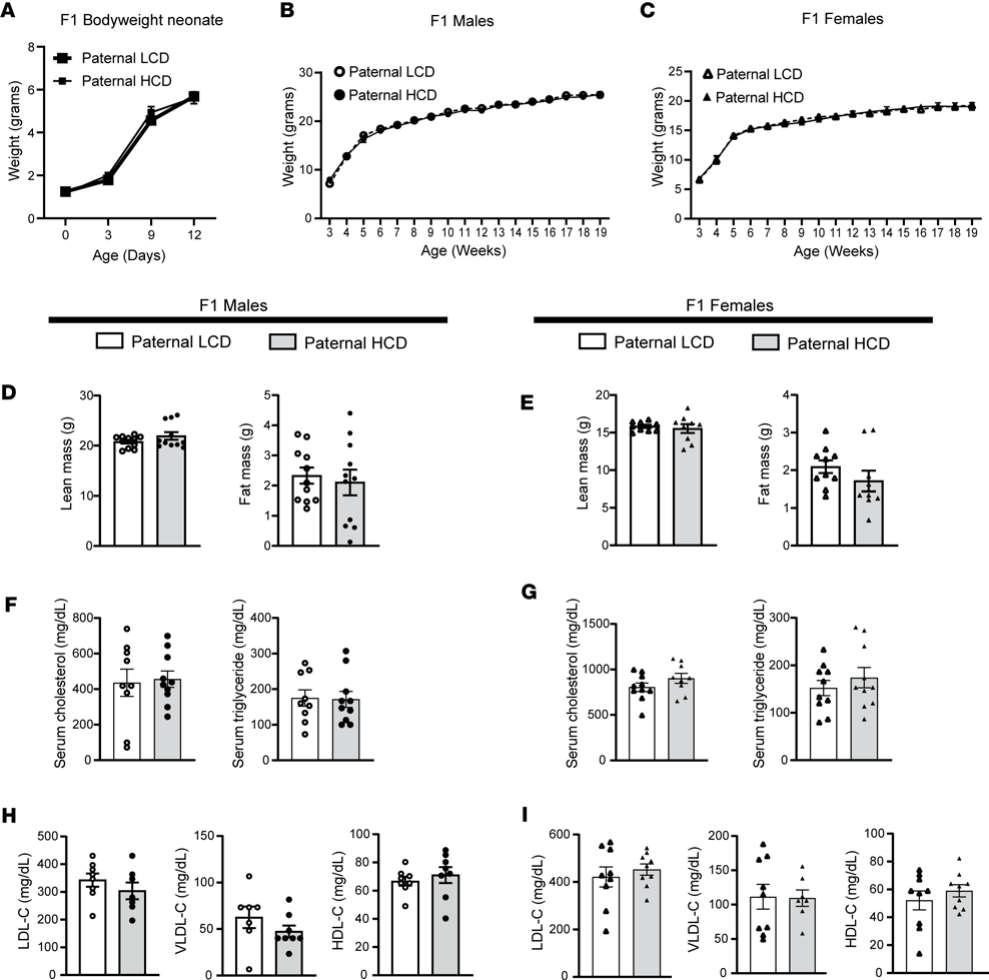
Paternal high-cholesterol diet feeding does not affect body weight or serum lipid levels in F1 offspring. Three-week-old male LDLR^–/–^ mice were fed an LCD or HCD diet for 8 weeks before mating with control female LDLR^–/–^ mice. Three-week-old F1 offspring were fed an LCD for 16 weeks and euthanized at 19 weeks of age. (**A**) Birth weight (day 0) and body weight of F1 pups before weaning (*n* = 5; 2-way ANOVA followed by Bonferroni’s multiple-comparison test). (**B** and **C**) Growth curves of male (**B**) and female (**C**) F1 offspring were measured (*n* = 9–14; 2-way ANOVA followed by Bonferroni’s multiple-comparison test). (**D** and **E**) Lean and fat mass were measure in male (**D**) and female (**E**) F1 offspring (*n* = 9–11, 2-sample, 2-tailed Student’s *t* test). (**F** and **G**) Serum cholesterol and triglyceride levels were measured in male and female offspring (*n* = 9–10, 2-sample, 2-tailed Student’s *t* test). (**H** and **I**) Serum lipoprotein fractions (VLDL-C, LDL-C, and HDL-C) were isolated from male (**H**) and female (**I**) F1 offspring and cholesterol levels from each fraction were measured (*n* = 7–9, 2-sample, 2-tailed Student’s *t* test). VLDL-C, very low-density lipoprotein cholesterol; LDL-C, low density lipoprotein cholesterol; HDL-C, high density lipoprotein cholesterol. All data are plotted as mean ± SEM.

F1 LDLR^–/–^ litters from LCD- or HCD-fed sires were weaned on P21 and fed an LCD for 16 weeks before euthanasia at 19 weeks of age. Paternal HCD feeding did not affect the birth weight or growth curve of the F1 litters before weaning (Figure 2A). After weaning, both male and female offspring from HCD-fed sires also had similar growth curve and BW compared with offspring from LCD-fed sires (Figure 2, B and C). In addition, body composition was analyzed, and paternal HCD feeding did not affect lean or fat mass of male or female offspring (Figure 2, D and E). There were also no changes on major organ weights between the offspring of LCD- or HCD-fed sires (Supplemental Figure 2, A and C). We also performed glucose-tolerance test (GTT) in the offspring and found that paternal HCD feeding did not affect the glucose tolerance in the offspring (Supplemental Figure 2, B and D).

We next measured the serum total cholesterol and triglyceride levels of the F1 offspring. While HCD feeding induced hypercholesterolemia in the sires, it did not affect the total cholesterol or triglyceride levels in the F1 offspring (Figure 2, F and G). In addition, offspring of HCD-fed sires also had similar VLDL, LDL, and HDL cholesterol levels as compared with the offspring of LCD-fed sires (Figure 2, H and I). Taken together, these results demonstrate that paternal HCD feeding did not affect metabolic phenotypes or serum lipid profiles in the offspring.

### Paternal hypercholesterolemia leads to exacerbated atherosclerosis in F1 female but not male LDLR^–/–^ offspring.

**Figure 3:**
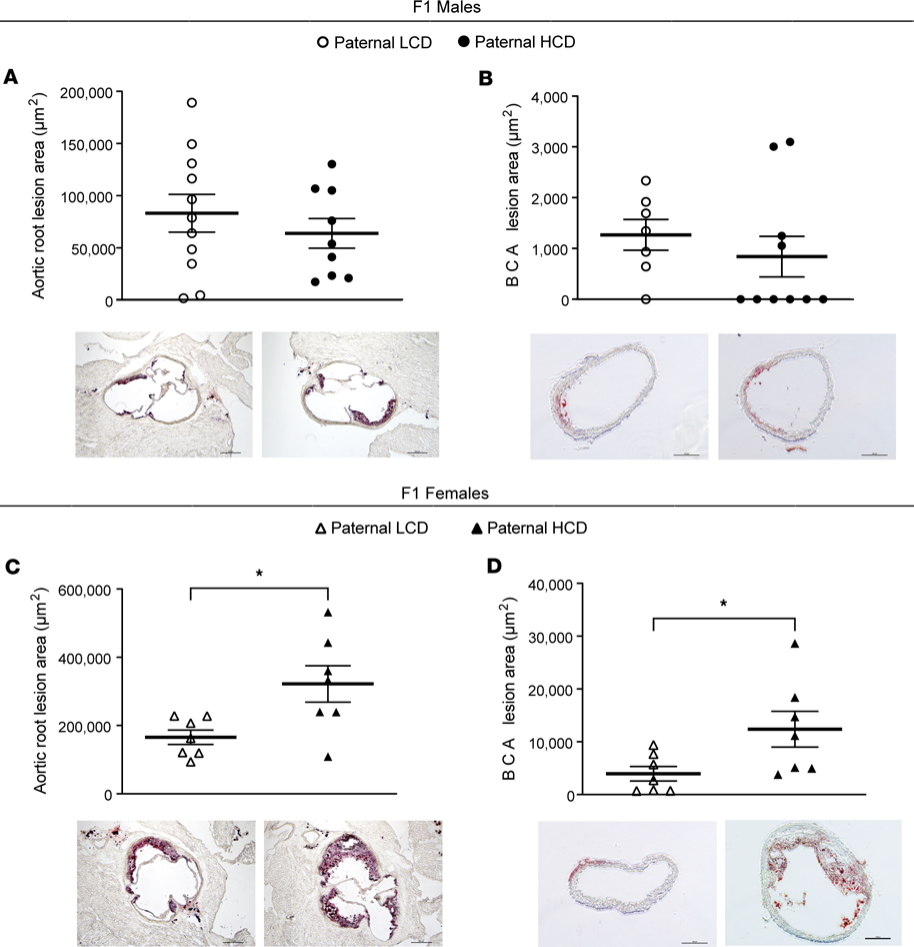
Paternal hypercholesterolemia increases atherosclerosis development in F1 female LDL receptor–deficient offspring. Three-week-old male LDLR^–/–^ mice were fed an LCD or HCD diet for 8 weeks before mating with control female LDLR^–/–^ mice. Three-week-old F1 descendants were fed an LCD for 16 weeks. (**A**–**D**) Quantitative analysis of the lesion area at the aortic root (**A** and **C**) or brachiocephalic artery (**B** and **D**) of male (**A** and **B**) and female (**C** and **D**) offspring (*n* = 7–11, *P < 0.05, 2-sample, 2-tailed Student’s *t* test). Representative Oil Red O–stained sections displayed below the quantification data. Scale bar: 200 μm. All data are plotted as mean ± SEM.

To investigate the effect of paternal hypercholesterolemia on atherosclerosis development in the offspring, we analyzed the atherosclerotic lesion area at the aortic root and brachiocephalic artery (BCA) of F1 LDLR^–/–^ mice. Paternal HCD feeding did not significantly affect atherosclerosis development in the male offspring since F1 male LDLR^–/–^ mice from HCD-fed sires had similar lesion sizes at the aortic root (63,910.06 ± 14,110.76 μm^2^ versus 83,190.78 ± 18,068.08 μm^2^) and BCA (840.31 ± 397.60 μm^2^ versus 1,267.31 ± 303.04 μm^2^) as compared with male offspring from LCD-fed sires (Figure 3, A and B). Interestingly, female offspring from HCD-fed sires developed significantly larger atherosclerotic lesions at both the aortic root (322,168.11 ± 53,287.69 μm^2^ versus 165,765.44 ± 21,105.56 μm^2^) and BCA (12,388.15± 3,410.31 μm^2^ versus 3,960.33 ± 1,368.80 μm^2^) as compared with female mice from LCD-fed sires (Figure 3, C and D).

**Figure 4:**
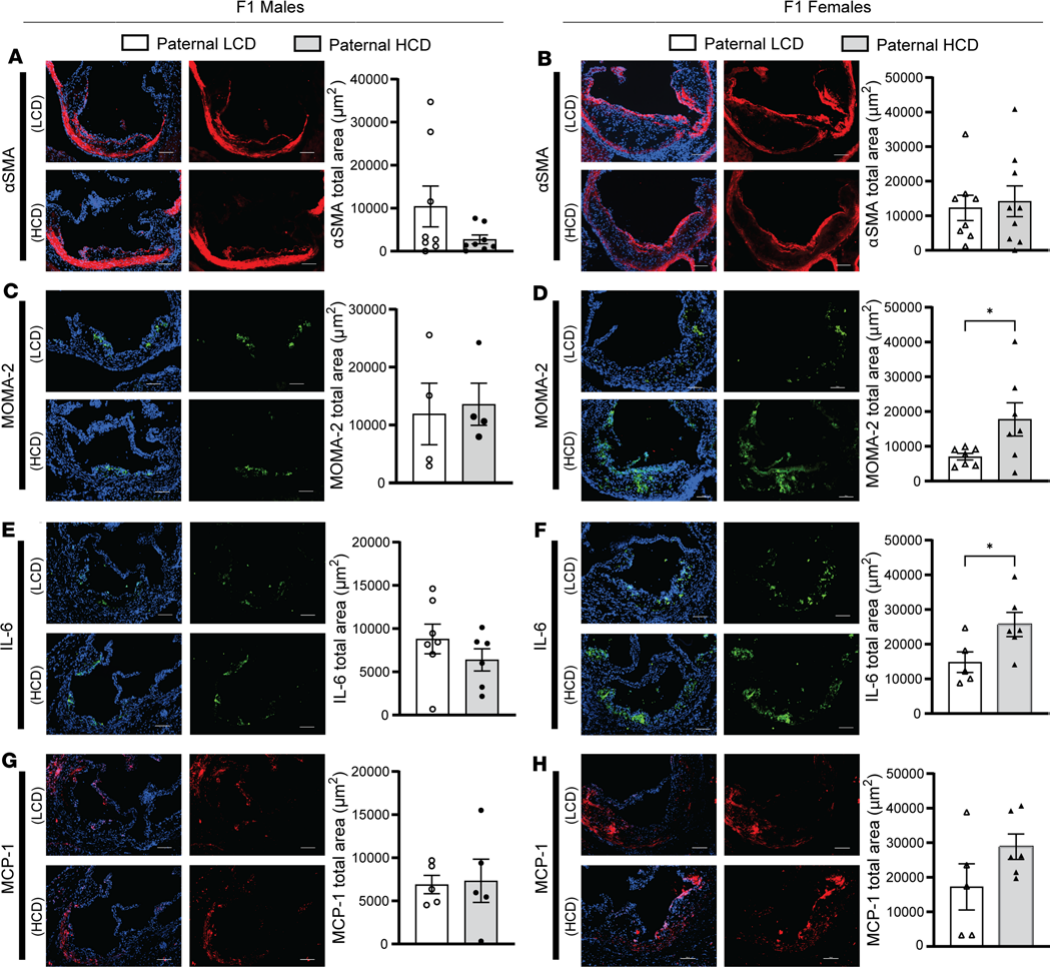
Paternal high-cholesterol diet feeding elicits macrophage accumulation and inflammation in atherosclerotic plaques of F1 offspring. Three-week-old male LDLR^–/–^ mice were fed an LCD or HCD diet for 8 weeks before mating with control female LDLR^–/–^ mice. Three-week-old F1 descendants were fed an LCD for 16 weeks. (**A**–**H**) Representative images of immunofluorescence staining of α-SMA (**A** and **B**), MOMA-2 (**C** and **D**), IL-6 (**E** and **F**), and MCP-1 (**G** and **H**) at the aortic root of male and female offspring. Scale bar: 100 μm. The nuclei were stained with DAPI (blue). Quantification analysis of staining areas is displayed as indicated (*n* = 4–9, *P < 0.05, 2-sample, 2-tailed Student’s *t* test). All data are plotted as mean ± SEM.

We next evaluated the atherosclerotic plaque composition of F1 LDLR^–/–^ offspring. While paternal HCD feeding did not affect the smooth muscle cell (SMC) contents in the plaques of F1 male or female offspring (Figure 4, A and B), paternal hypercholesterolemia led to significantly increased macrophage contents in the atherosclerotic plaques of F1 female but not male mice (Figure 4, C and D). We also assessed the collagen contents and necrotic cores in the atherosclerotic plaques of these mice and found that paternal HCD feeding did not affect collagen contents in male offspring but tended to increase collagen levels in female offspring (Supplemental Figure 3, A and B). In addition, the necrotic core sizes were not affected by paternal hypercholesterolemia in either male or female offspring (Supplemental Figure 3, C and D).

Since inflammatory responses are the driving force of atherosclerosis development (47–49), we then analyzed the expression of key proinflammatory proteins, including IL-6 and MCP1, in the atherosclerotic plaques of the offspring. While MCP1 protein levels in the offspring were not affected by paternal hypercholesterolemia, paternal HCD-feeding led to significantly increased IL-6 protein levels in the atherosclerotic plaques of F1 female but not male LDLR^–/–^ mice (Figure 4, E–H). Collectively, these findings demonstrate that paternal hypercholesterolemia elicited sex-specific atherogenic effects in the offspring of LDLR^–/–^ mice that were independent of serum lipid levels.

### Transcriptomic analysis reveals altered atherosclerosis-related gene expression in the intima of female offspring from HCD-fed sires.

**Figure 5:**
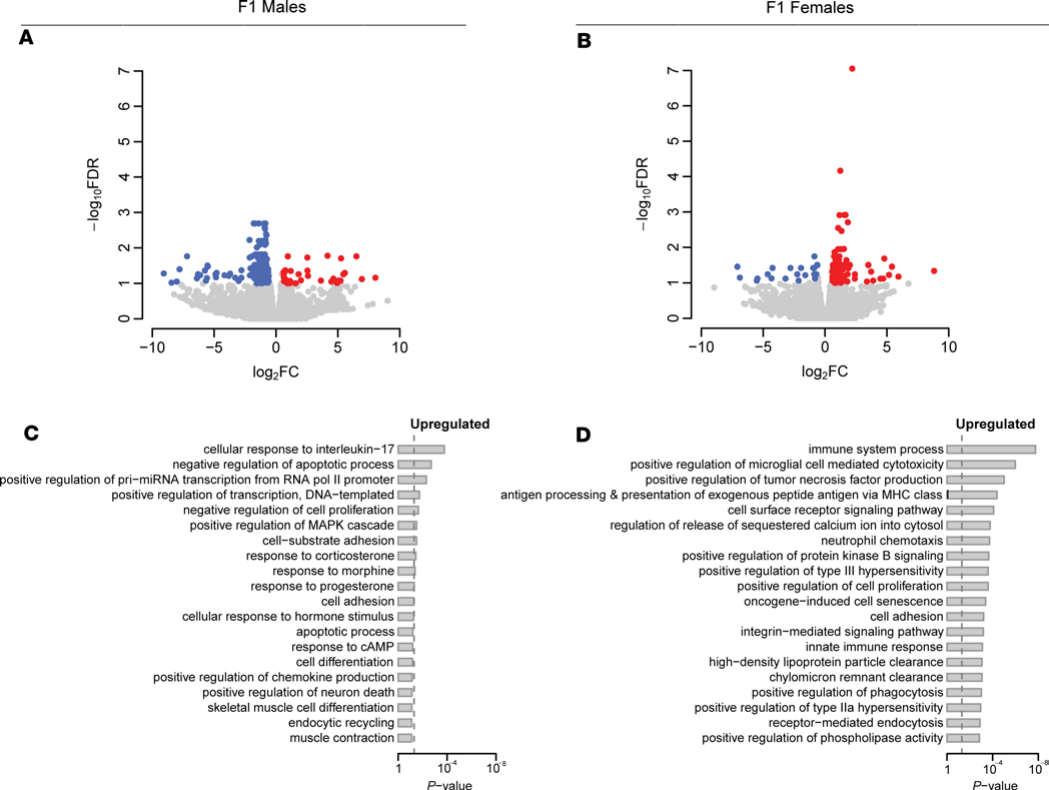
Paternal hypercholesterolemia elicits transcriptomic changes in the intima of F1 LDL receptor–deficient mice. Three-week-old male LDLR^–/–^ mice were fed an LCD or HCD diet for 8 weeks before mating with control female LDLR^–/–^ mice. Three-week-old F1 descendants were fed a LCD for 16 weeks. Total RNAs were isolated from the intima of F1 offspring and used for RNA-Seq analysis. (**A** and **B**) Volcano plots of differentially expressed genes (DEGs) in the intima of male offspring (**A**) and female offspring (**B**) from HCD-fed LDLR^–/–^ sires. Colored dots represent the enriched (red dots) or depleted (blue dots) DEGs with a FDR of < 0.1 and a FC > 1.5 as a cut-off threshold. (**C** and **D**) GOBP terms significantly associated with upregulated DEGs in intima of male offspring (**C**) and female offspring (**D**) from HCD-fed sires. The *P* values were computed by the modified Fisher’s exact test using the DAVID bioinformatics tool. The vertical dash line indicates the significance level of α = 0.05. The *y* axis displays the GOBP terms, while the *x* axis displays the *P* values (*n* = 4–7 each group).

To further understand the paternal hypercholesterolemia-elicited atherogenic effects in offspring, RNAs were isolated from the intima of F1 male and female offspring for RNA-Seq analysis. RNA-Seq analysis revealed that paternal HCD feeding led to 197 differentially expressed genes (DEGs) in the intima of male offspring, and most of them (164 genes) were downregulated genes (Figure 5A and Supplemental Table 1). By contrast, paternal HCD feeding led to 147 upregulated genes but only 21 downregulated genes in the intima of female offspring (Figure 5B and Supplemental Table 2).

We then performed gene ontology (GO) analysis (50, 51), and the results uncovered that the upregulated genes by paternal HCD feeding in the F1 female but not male intima were enriched in several biological processes related to atherosclerosis or inflammation, including “immune system process,” “positive regulation of tumor necrosis factor production,” “neutrophil chemotaxis,” “integrin-mediated signaling pathway,” “innate immune response,” “positive regulation of phagocytosis,” and “receptor-mediated endocytosis” (Figure 5, C and D).

**Figure 6:**
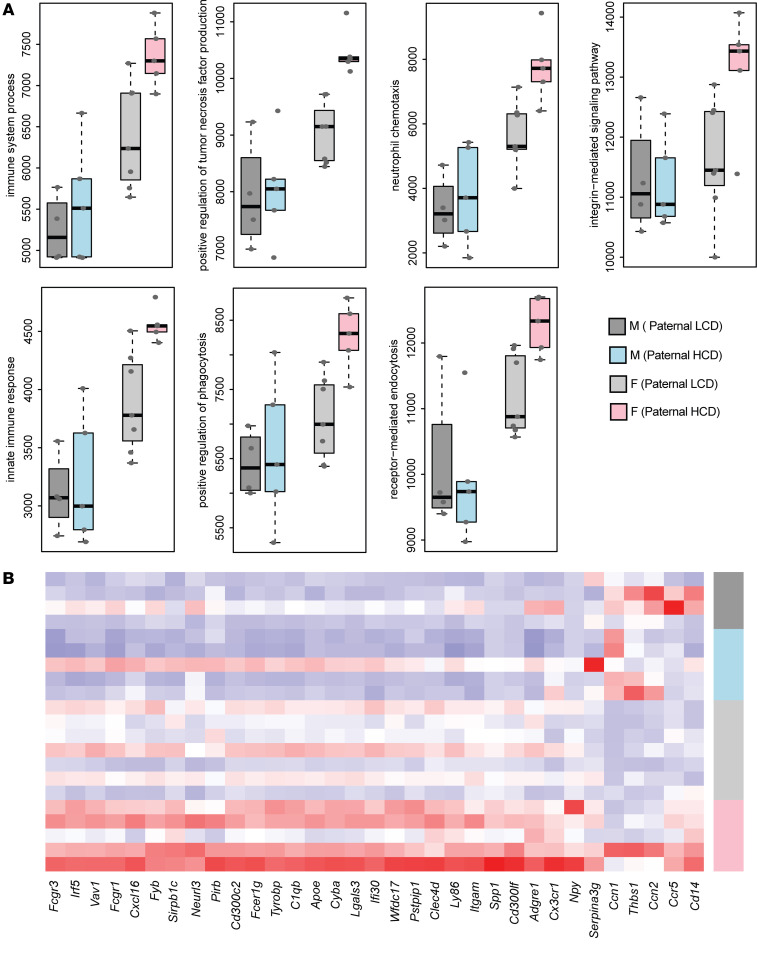
Paternal hypercholesterolemia alters atherosclerosis-related gene expression in the intima of female F1 offspring. Three-week-old male LDLR^–/–^ mice were fed an LCD or HCD diet for 8 weeks before mating with control female LDLR^–/–^ mice. Three-week-old F1 descendants were fed a LCD for 16 weeks. Total RNAs were isolated from the intima of F1 offspring and used for RNA-Seq analysis. (**A**) Gene set scores of the prioritized GOBP terms of male and female offspring from LCD or HCD-fed sires. The gene set score was calculated using the FAIME algorithm. (**B**) Heatmap representation of DEGs involved in the indicated GOBP terms. Each column shows 1 individual gene, and each row shows a biological replicate of mouse. Red represents relatively increased gene expression, whereas blue denotes downregulation (*n* = 4–7 each group).

The Functional Analysis of Individual Microarray Expression (FAIME) algorithm was then performed to evaluate the gene set scores of the GO biological process (GOBP) terms (43). FAIME results confirm that paternal hypercholesterolemia led to significant upregulation of gene set scores of those GOBP terms associated with atherosclerosis or inflammation processes in female but not male offspring (Figure 6A). Consistently, the genes associated with these GOBP terms were upregulated in the intima of F1 female but not male mice from HCD-fed sires as shown in the heatmap (Figure 6B).

### CCN1 and CCN2 proteins are elevated in the lesions of F1 females from HCD-fed sires and promote proatherogenic gene expression in endothelial cells in vitro.

While paternal HCD feeding led to significant upregulation of some known proatherogenic and proinflammatory genes or pathways in the intima of F1 female but not male offspring, several other DEGs induced by paternal hypercholesterolemia have not been well studied. To explore genes or pathways that may contribute to paternal hypercholesterolemia-elicited atherogenic effects in the offspring, we investigated several candidate genes including *Ccn1* and *Ccn2*, which are 2 upregulated intimal genes in female offspring that are implicated in regulating vascular function and inflammatory responses (52–55).

**Figure 7:**
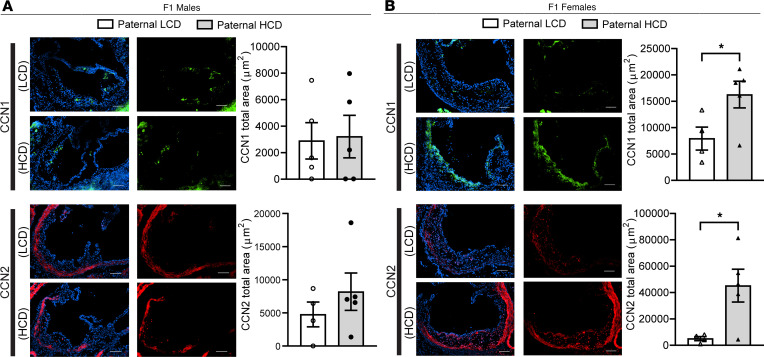
CCN1 and CCN2 proteins are elevated in the atherosclerotic lesions of F1 female LDL receptor–deficient descendants from high-cholesterol diet–fed sires. Three-week-old male LDLR^–/–^ mice were fed an LCD or HCD diet for 8 weeks before mating with control female LDLR^–/–^ mice. Three-week-old F1 descendants were fed an LCD for 16 weeks. (**A** and **B**) Representative immunofluorescence images of CCN1 (green) and CCN2 (red) at the aortic root of F1 male (**A**) and female (**B**) offspring. The nuclei were stained with DAPI (blue). Scale bar: 100 μm. Quantification analysis of stating areas is displayed as indicated (*n* = 4–5, *P < 0.05, 2-sample, 2-tailed Student’s *t* test). All data are plotted as mean ± SEM.

**Figure 8:**
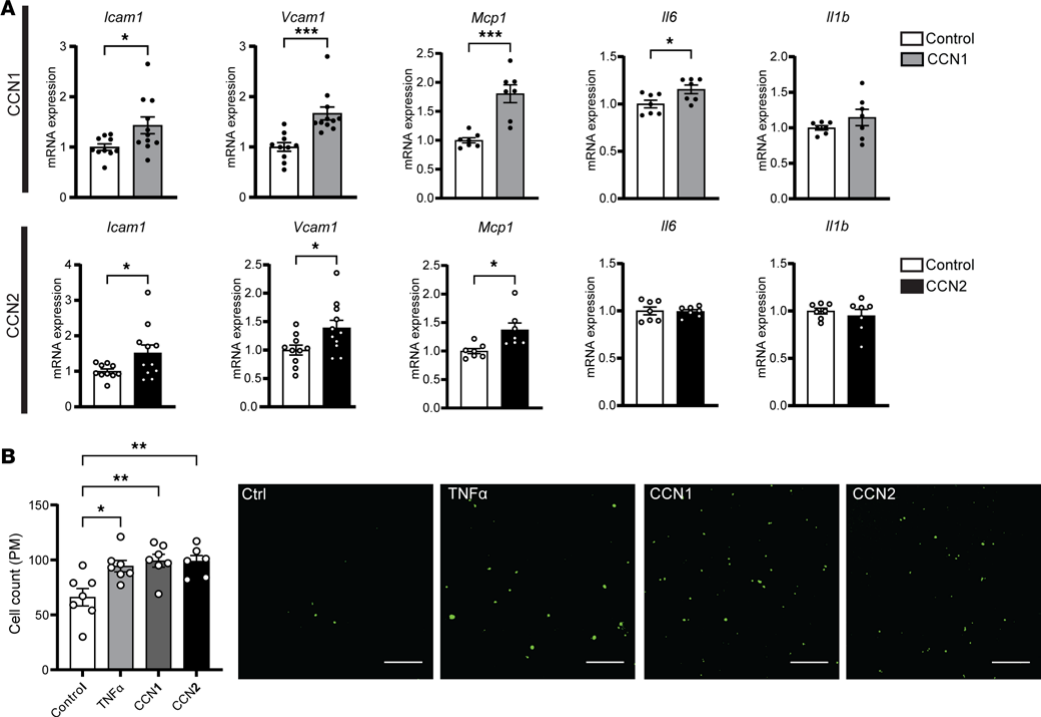
CCN1 and CCN2 proteins promote proatherogenic gene expression in endothelial cells in vitro. (**A**) Human endothelial cells, HMEC-1 cells, were treated with 1 μg/mL CCN1 or CCN2 for 4 hours followed by total RNA isolation. The expression levels of indicated genes were analyzed by qPCR (*n* = 7–11, *P < 0.05, ***P < 0.001, 2-sample, 2-tailed Student’s *t* test). (**B**) HMEC-1 endothelial cells were pretreated with 50 ng/mL CCN1 or CCN2 or 10 ng/mL TNF-α for 24 hours before incubating with calcein acetoxymethyl–stained peritoneal macrophages isolated from LDLR^–/–^ mice for 4 hours. Adhered cells were counted under a fluorescence microscope. Quantitative analysis of the adhered cells is displayed to the left of representative images (*n* = 6–7, *P < 0.05, **P < 0.01, 1-way ANOVA followed by Bonferroni’s multiple-comparison test). All data are plotted as mean ± SEM.

We first evaluated the protein levels of CCN1 and CCN2 in the atherosclerotic plaques by immunofluorescence staining. Consistent with RNA-Seq results, both CCN1 and CCN2 protein levels were significantly elevated in the atherosclerotic plaques of F1 female but not male offspring from HCD-fed sires as compared with the offspring from LCD-fed sires (Figure 7, A and B). Interestingly, we found that CCN1 and CCN2 proteins can be colocalized with both macrophages and endothelial cells in atherosclerotic lesions (Supplemental Figure 4). To elucidate the potential role of CCN1 and CCN2 in regulating endothelial cell function related to atherosclerosis, we treated human endothelial cells, HMEC-1 cells (43, 56), with recombinant human CCN1 or CCN2 proteins. Interestingly, both CCN1 and CCN2 protein treatments led to increased expression of several key proatherogenic genes, including *Vcam1*, *Icam1*, *Mcp1*, and *Il6*, in HMEC-1 cells (Figure 8A).

To determine whether CCN1 and CCN2 can regulate endothelial cell function, we examined the effect of CCN1 and CCN2 treatment on macrophage adhesion properties to endothelial cells. Freshly isolated primary macrophages from LDLR^–/–^ mice were incubated with HMEC-1 cells treated with CCN1, CCN2, or TNF-α. Similar to TNF-α treatment, CCN1 and CCN2 treatment increased macrophage adhesion to HMEC-1 cells (Figure 8B). Previous studies have demonstrated that CCN1 and CCN2 can activate NF-κB signaling in other cell types (52, 53, 57, 58). NF-κB is a master transcriptional factor that regulates immune responses and has also been shown to promote atherosclerosis development (48, 49, 59). Interestingly, we found that treatment of HMEC-1 cells with CCN1 and CCN2 led to increased phosphorylation of the NF-κB p65 subunit and induction of p65 nuclear colocalization (Supplemental Figure 5), indicative of NF-κB signaling activation. Taken together, these results demonstrate that paternal HCD feeding leads to increased intimal expression of CCN1 and CCN2 in female offspring, which may promote endothelial cell dysfunction through NF-κB signaling pathway.

### PANDORA-Seq detects differentially expressed tsRNAs and rsRNAs in the sperm of hypercholesterolemic LDLR^–/–^ male mice.

Sperm contain specific sncRNAs, including tsRNAs and rsRNAs, that have been reported to function as transmissible epigenetic regulators to mediate offspring’s metabolic phenotypes (13, 29, 39). Many sperm tsRNAs and rsRNAs are highly modified, and these RNA modifications render sncRNAs undetectable by widely used traditional RNA-Seq methods. To overcome this limitation, we utilized a newly developed small RNA-Seq method, PANDORA-Seq (40), which can unveil a more comprehensive tsRNA/rsRNA landscape in sperm and tissues (40, 43).

**Figure 9:**
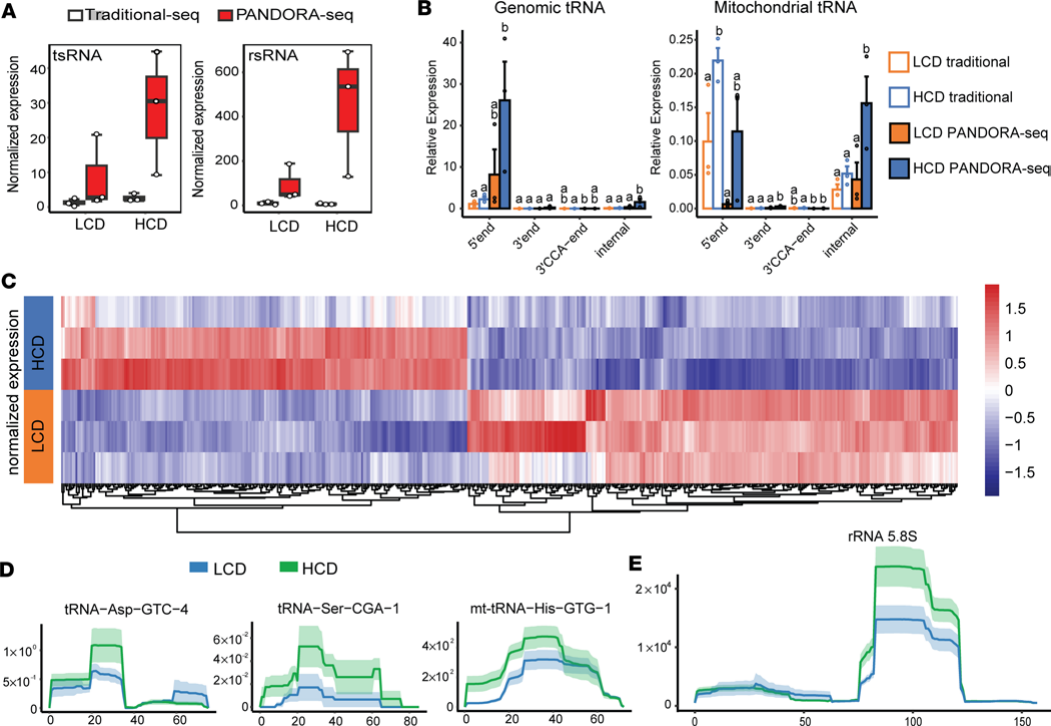
PANDORA-Seq reveals significantly changed sperm tsRNAs and rsRNAs induced by high-cholesterol diet feeding in male LDL receptor–deficient mice. Three-week-old male LDLR^–/–^ mice were fed an LCD or HCD for 9 weeks. Total RNAs were isolated from the sperm and used for PANDORA-Seq and traditional small RNA sequencing. (**A**) Sperm tsRNA and rsRNA relative expression (normalized to miRNAs) under traditional sequencing and PANDORA-Seq protocols. (**B**) Sperm tsRNA responses to traditional sequencing and PANDORA-Seq in regard to different genomic or mitochondria tRNA origins (5′tsRNA, 3′tsRNA, 3′tsRNA-CCA end, and internal tsRNAs). The *y* axes represent the relative expression levels compared with total reads of miRNA. Different letters above the bars indicate statistically significant differences (*P* < 0.05). Same letters indicate *P* > 0.05. Statistical significance was determined by 2-sided 1-way ANOVA with uncorrected Fisher’s least significant difference test. All data are plotted as mean ± SEM. (**C**) Heatmap representation of differentially expressed sperm tsRNAs detected by PANDORA-Seq. Biological replicates are represented in each row. Red represents relatively increased expression, whereas blue represents decreased expression with adjusted *P* < 0.05 and FC > 2 as the cutoff threshold. (**D** and **E**) Dynamic responses to LCD or HCD of representative sperm tsRNAs (**D**) and rsRNAs (**E**) detected by PADNORA-Seq. Mapping plots are presented as mean ± SEM (*n* = 3 in each group).

To investigate whether exposure to HCD can elicit sncRNA changes in the sperm of LDLR^–/–^ mice that may confer proatherogenic effects in offspring, we isolated total RNAs from sperm of LCD- and HCD-fed male LDLR^–/–^ mice and conduced both traditional RNA-Seq and PANDORA-Seq. The sequence data were then analyzed using the *SPORTS1.1* bioinformatics tool (40, 60). Consistent with our previous results (33, 40), PANDORA-Seq but not traditional RNA-Seq uncovered abundant tsRNA and rsRNA populations in sperm of both LCD- and HCD-fed mice (Figure 9A). In addition, only PANDORA-Seq can detect that exposure to HCD induced differentially expressed sperm total tsRNAs and rsRNAs. We then analyzed the origins of sperm tsRNA related to their loci from tRNA precursors, including 5′tsRNAs, 3′tsRNAs, 3′tsRNAs with a CCA end, and internal tsRNAs. PANDORA-Seq results show elevated relative expression of specific genomic and mitochondrial-derived (mt-derived) tsRNA origins (normalized to miRNAs) as compared with traditional RNA-Seq results (Figure 9B). Interestingly, most genomic tsRNAs were derived from the 5′-end of mature tRNAs while, many mitochondrial tsRNAs originated from the 5′end and internal regions of tRNAs (Figure 9B). Overall, PANDORA-Seq detected 813 differentially regulated tsRNAs and rsRNAs elicited by HCD feeding as shown in the heatmap (Figure 9C and Supplemental Table 3). Furthermore, mapping of tsRNA and rsRNA expression patterns on selected individual tRNA or rRNA length scales, including tRNA-Asp-GTC, tRNA-Ser-CGA, mt-tRNA-His-GTG, and 5.8S rRNA, revealed that these tsRNAs/rsRNAs also contain distinct dynamic responses to HCD feeding (Figure 9, D and E, and Supplemental Figure 6, A and B). The functions of these tsRNAs or rsRNAs as epigenetic regulators are mostly unknown, but previous studies from us and others have demonstrated that sperm sncRNA fractions enriched by tsRNAs/rsRNAs contributed to the epigenetic inheritance of paternally acquired metabolic disorder through zygotic injection (13, 14, 61–63). Thus, it is plausible that these altered tsRNAs or rsRNAs contribute to paternal hypercholesterolemia-induced intergenerational atherogenic effects.

### Exposure to HCD alters sncRNA biogenesis–related genes in the epididymis.

**Figure 10:**
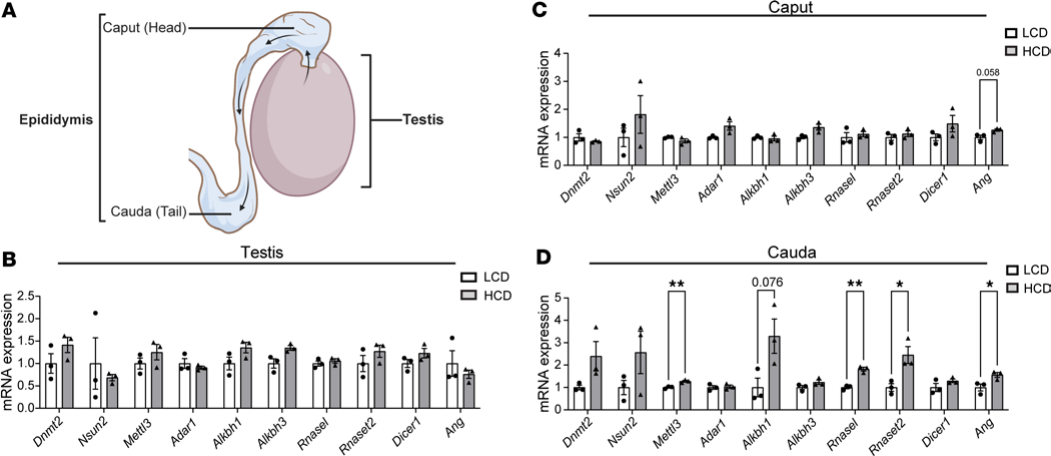
Hypercholesterolemia alters the expression of sncRNA biogenesis–related genes in cauda epididymis. (**A**) Schematic of the testis and epididymis. Sperm generated in the testis undergo maturational changes during transiting through the caput and cauda epididymis. (**B**–**D**) Three-week-old male LDLR^–/–^ mice were fed an LCD or HCD for 9 weeks. Total RNAs were isolated from the testis (**B**), and caput (**C**), and cauda (**D**) epididymis. The expression levels of indicated genes related to sncRNA biogenesis were analyzed by qPCR (*n* = 3, *P < 0.05, **P < 0.01, 2-sample, 2-tailed Student’s *t* test). All data are plotted as mean ± SEM.

Biogenesis of sperm sncRNAs, including tsRNAs, mainly occurs in the epididymis rather than testis (29, 38, 64). To determine whether changes in the sperm sncRNA profile from HCD-fed LDLR^–/–^ mice are due to altered tsRNA/rsRNA biogenesis in the epididymis, we examined the expression of sncRNA biogenesis–related genes in the testis, caput, and cauda epididymis (Figure 10A). HCD feeding did not significantly affect the expression of sncRNA biogenesis–related genes in the testis or caput epididymis (Figure 10, B and C). Interestingly, exposure to HCD led to increased expression of several key sncRNA biogenesis–related genes inducing tsRNA cleavage enzymes *Ang*, *Rnasel*, and *Rnaset2* and RNA modification enzymes *Mettl3* in the cauda epididymis (Figure 10D). There results indicate that hypercholesterolemia affects tsRNA/rsRNA biogenesis–related gene expression in the epididymis (primarily in the cauda), leading to the altered sperm sncRNA landscape.

### Hypercholesterolemia-stimulated sperm tsRNAs/rsRNAs induce early transcription changes in murine embryoid bodies.

We previously demonstrated that tsRNAs and rsRNAs can affect murine embryonic stem cell (mESC) differentiation and embryoid body (EB) transcriptome (40). To explore the potential functions of the dysregulated sperm tsRNAs/rsRNAs, we transfected a pool of selected tsRNAs and rsRNAs including tsRNA-Glu-CTC/TTC, mt-tsRNA-His-GTG, rsRNA-18S, and 2 rsRNAs derived from rRNA-28S that were stimulated by HCD feeding into mESCs followed EB formation (Supplemental Table 3). Interestingly, we found that transfection of these tsRNAs/rsRNAs led to significantly increased expression of several proatherogenic or endothelial dysfunction–related genes including Endothelin (*End1*), E-selectin (*Sele*), and *Il1b* (Supplemental Figure 7). In addition, the expression levels of *Sox17*, a key transcription factor regulating cardiovascular development and endoderm differentiation (65, 66), was also elevated by transfection of those tsRNAs/rsRNAs (Supplemental Figure 7). Thus, HCD feeding–stimulated sperm tsRNAs and rsRNAs may have adverse effects on offspring cardiovascular development or atherogenesis.

## Discussion

**Figure 11:**
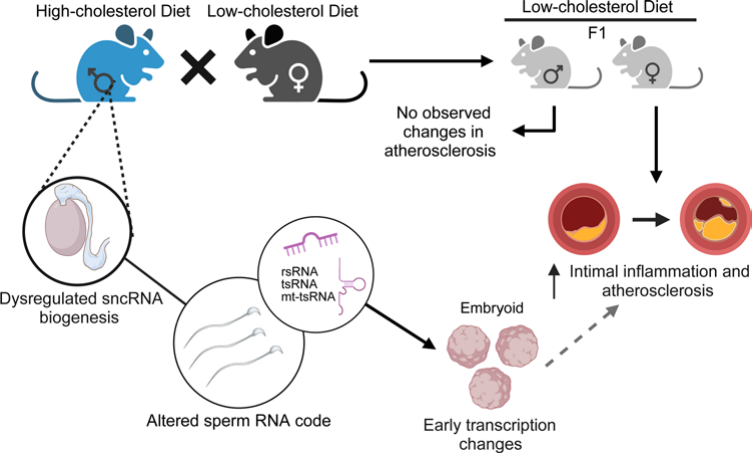
Schematic of the effect of paternal exposure to the high-cholesterol diet on sperm sncRNAs and offspring atherosclerosis development. Paternal high-cholesterol feeding led to significantly increased atherosclerosis and intimal inflammation in F1 female, but not male, LDLR^–/–^ offspring. PANDORA-Seq identified an altered sncRNA landscape in the sperm of high-cholesterol diet–fed LDLR^–/–^ sires. Overexpression of a pool of sperm tsRNAs/rsRNAs that were upregulated in the sperm of hypercholesterolemic sires induced transcription changes in embryoid bodies that may contribute the increased atherosclerosis in the adult offspring. The image was created with BioRender.com.

Increasing lines of evidence demonstrate that parental exposure–acquired diseased phenotypes can be encoded in the germline epigenome and transmitted to future generations, leading to adverse health outcomes (31, 67–70). While many studies have investigated the adverse effects of maternal exposures to suboptimal factors on offspring cardiometabolic health, little is known about the contribution of paternal factors to CVD risk in offspring. In the current study, we investigated the effects of paternal HCD feeding on offspring atherosclerosis development in LDLR^–/–^ mice (Figure 11). We found that paternal hypercholesterolemia significantly increased atherosclerosis in F1 female but not male LDLR^–/–^ mice. Interestingly, paternal hypercholesterolemia–elicited sex-specific atherogenic effects in the offspring were independent of serum lipid levels or metabolic dysfunction. RNA-Seq analysis then revealed that paternal hypercholesterolemia can lead to upregulation of many known proatherogenic genes or pathways in the intima of F1 female mice. We then identified 2 potentially novel proatherogenic genes, *Ccn1* and *Ccn2*, that may contribute to increased atherosclerosis development in female F1 LDLR^–/–^ mice by regulating proatherogenic gene expression in endothelial cells. Using the innovative PANDORA-Seq method, we revealed that HCD feeding can alter the sperm tsRNA and rsRNA landscape, which may contribute to paternally acquired atherosclerosis in offspring. We also discovered that exposure to HCD alters sncRNA biogenesis–related genes in the epididymis but not testis, which may lead to the altered sperm sncRNA landscape. Although the exact mechanism through which sperm tsRNAs and rsRNAs mediate paternally acquired atherosclerosis in offspring remains elusive, we found that hypercholesterolemia-stimulated sperm tsRNAs and rsRNAs can induce early transcription changes in murine EBs. Several proatherogenic or endothelial dysfunction–related genes were upregulated by these tsRNAs/rsRNAs in EBs, and this occurrence may lead to adverse effects on cardiovascular development or increased atherosclerosis in the offspring. Our study demonstrates that paternal dietary exposure can elicit sex-specific intergenerational atherosclerosis in offspring using appropriate animal models.

Human longitudinal studies, such as the Framingham Offspring Study, have suggested that the presence of parental CVD risk factors can lead to increased CVD in offspring (7, 20, 71). Moreover, clinical and animal studies have demonstrated that maternal exposures to suboptimal environmental factors such as unhealthy diets and toxicants may lead to CVD complications, including hypertension (25, 72, 73), increased intima-media thickness (74, 75), altered lipid profile (76), and increased atherosclerosis (21, 23, 26, 27), in offspring. In addition to maternal influences, offspring CVD can be closely associated with paternal CVD risk factors in humans (7–9, 20). However, limited studies have investigated paternal exposure–elicited cardiometabolic disease risk in animal models. Furthermore, most of the paternal studies, including our previous ones, have focused on the intergenerational transmission of paternally acquired metabolic disorders but not CVD (12–14, 19, 29–31, 33, 63, 77). In the current study, we utilized the widely used atherosclerosis-prone LDLR^–/–^ mouse model to investigate the effect of paternal HCD feeding on offspring atherosclerosis development. We report that paternal hypercholesterolemia can lead to increased atherosclerosis development and intimal proatherogenic gene expression in female LDLR^–/–^ descendants, demonstrating the atherogenic effects of paternal unhealthy diet exposure in offspring.

It is intriguing that paternal hypercholesterolemia can lead to increased atherosclerosis in female but not male offspring in LDLR^–/–^ mice. Parental exposure–elicited sex-specific effects on offspring cardiometabolic health have been reported in both human and animal studies (8, 78). For example, human studies suggest that maternal undernutrition may lead to increased adiposity and BW in middle-age female but not male descendants (78, 79). In animal studies, maternal hypercholesterolemia also led to early atherosclerosis development in female but not male offspring in apolipoprotein E–deficient mice (26, 27) and circulating lipid levels were unlikely to be the main factor contributing to accelerated atherosclerosis development in the offspring. An earlier study in rats claimed that F1 female but not male offspring from HFD-fed sires had glucose tolerance impairment (12). A very recent report showed that paternal HFD feeding in mice led to increased metabolic disorders in male offspring, but female offspring were not included in the study (32). We also demonstrated that paternal exposure to a plastic-associated EDC, dicyclohexyl phthalate, elicited sex-specific transgenerational effects in F2 offspring in mice (33). Interestingly, paternal EDC exposure induced glucose intolerance in F2 female but not male descendants (33). The mechanisms responsible for sex differences in the intergenerational inheritance of metabolic phenotypes remain largely unknown. Several potential mechanisms related to sex hormones (30, 78, 80, 81), sex chromosomes (82, 83), mitochondrial function (84, 85), or developmental epigenetic reprogramming (78) have been suggested. Furthermore, the sex-difference in offspring phenotype may also be derived from the different sperm RNA information carried in X versus Y sperm (86), with mechanisms that remain unknown. Sexual dimorphic responses to early life perturbations, from either ancestral side, remain an important but understudied research topic. Future studies are required to understand the detailed mechanisms for sex differences in the intergenerational transmission of paternally acquired cardiometabolic phenotypes.

In addition to known proatherogenic genes, our RNA-Seq analysis identified 2 potentially new proatherogenic genes, *Ccn1* and *Ccn2*, that may contribute to increased atherosclerosis in F1 females. CCN1 and CCN2 are matricellular proteins that are essential for cardiovascular development during embryogenesis (52, 53, 87, 88). The expression of these genes is reduced later in life and can be increased in atherosclerotic plaques of both humans and rodents (52–55, 89, 90). We found that CCN1 and CCN2 can be colocalized with both endothelial cells and macrophages within atherosclerotic lesions. The function of CCNs in macrophages has been previously reported (91, 92), but their role in endothelial cell biology remains unclear. We then discovered that CCN1 and CCN2 proteins can activate NF-κB signaling and stimulate proatherogenic gene expression in endothelial cells in vitro. Thus, it is plausible that the increased endothelial *Ccn1/Ccn2* expression may affect other cell types such as macrophages to contribute to the exacerbated atherosclerosis in F1 female mice from HCD-fed sires.

Emerging evidence supports the notion that the paternal environment can influence offspring health, but the underlying molecular mechanisms remains largely unknow. Recent studies by us and others have demonstrated that environmental exposures, including unhealthy diet, environmental toxicants, and stress, can alter the sperm RNAs to mediate intergenerational inheritance (13, 14, 32, 33, 63, 93–97). We previously discovered that mouse sperm is enriched with a subset of sncRNAs, including tsRNAs and rsRNAs, that contribute significantly to intergenerational inheritance of paternally acquired metabolic disorders (13, 14, 29, 38, 39, 98). The injection of sperm total RNAs or tsRNA/rsRNA-enriched RNA fractions from HFD-exposed sires can induce offspring phenotypes that fully or partially recapitulated the paternal environmental input, including obesity and altered glucose metabolism (13, 14), thus demonstrating that sperm RNAs have causal effects in mediating intergenerational inheritance in mammals. Many sperm tsRNAs/rsRNAs are highly modified, and these RNA modifications may shape the RNA secondary structures and biological properties to mediate epigenetic inheritance (14, 38, 99). While RNA modifications are essential for the functions of sncRNAs such as tsRNAs (38, 100, 101), these modifications can also interfere with either reverse transcription or adaptor ligation process when constructing cDNA library for RNA-Seq analysis, thereby limiting the sncRNA detection capacity of the traditional RNA-Seq method. To address this obstacle, we utilized the PANDORA-Seq method, which enabled us to identify highly modified sncRNAs that are otherwise undetectable by traditional RNA-Seq (40, 41). In the current study, PANDORA-Seq revealed abundant tsRNA/rsRNA expression in the sperm of LDLR^–/–^ mice as compared with traditional RNA-Seq results. These results are consistent with our recent results demonstrating that these understudied tsRNAs/rsRNAs are much more abundant than the well-studied miRNAs across many human and murine tissues or cells, including sperm (33, 40, 43, 100, 101). Interestingly, HCD-feeding also led to upregulation of sperm mt-tsRNAs that have recently been linked to mitochondrial dysfunction in HFD-fed mice and obese humans (32, 102). These sperm sncRNAs has also been shown to affect altered early-embryo transcription, which may lead to offspring metabolic disorders (32). Therefore, the sperm nuclear and mitochondrial-derived sncRNAs could act as key factors in mediating paternal hypercholesterolemia–elicited atherosclerosis in offspring.

To explore how the altered sperm sncRNAs may affect offspring cardiovascular heath, we investigated the effect of a pool of sperm tsRNA/rsRNA on murine EB transcription changes. We and others have demonstrated that tsRNAs/rsRNAs, including sperm mt-tsRNAs, can affect EB or early-embryo transcription (32, 40). Therefore, we also included HCD-stimulated sperm mt-tsRNAs in our assays. Interestingly, we found that overexpression of these sperm tsRNAs/rsRNAs, including sperm mt-tsRNA, led to increased expression of proatherogenic genes such as *End1*, *Sele*, and *Il1b* in murine EBs. While IL-1β has been well established to promote atherosclerosis, END1 and SELE are markers for endothelial dysfunction and may also contribute to the development of CVD (103–106). In addition, the expression levels of *Sox17* were also elevated by overexpression of those tsRNAs/rsRNAs. Sox17, an essential factor controlling endothelial and hematopoietic cell lineages (65, 66), also plays an important role in regulating endothelial cell function and can be upregulated in response to endothelial dysfunction (65). It is plausible that tsRNA/rsRNA-elicited early transcription changes in EBs lead to adverse effects on of the increased expression of proatherogenic genes, causing adverse effects on atherogenesis later in life. Future studies are needed to investigate the functions and detailed mechanisms of these tsRNAs/rsRNAs in mediating HCD feeding–induced intergenerational atherosclerosis risk.

It is interesting that HCD feeding can alter the sperm tsRNA/rsRNA landscape. Biogenesis of sperm sncRNAs, including tsRNAs, mainly occurs in the epididymis (29, 38, 64). Epididymal but not testicular sperm sncRNAs are also more susceptible to unhealthy diet exposures (32). While the mechanisms underlying the diet-induced sperm sncRNA changes remain unknown, it is possible that dietary changes altered the levels or activities of related enzymes for RNA modifications and cleavage enzymes, leading to altered tsRNA/rsRNA biogenesis. We found that HCD feeding mainly affected the expression of RNA biogenesis–related genes in cauda epididymis but not in testis or caput epididymis. Specifically, exposure to HCD led to significantly increased expression of cleavage enzymes *Ang*, *Rnasel*, and *Rnaset2* and modification enzyme *Mettl3* in cauda epididymis. *Ang*, *Rnasel*, and *Rnaset2* are ribonucleases that cleave tRNAs into tsRNAs and have been shown to alter the sperm tsRNA/rsRNA composition (63, 107). *Ang* has also been well documented to be induced under various stress conditions including inflammation (63, 108). *Mettl3* is a m^6^A methyltransferase that can also regulate RNA translation and many biological processes (109). It is plausible that the high cholesterol environment affects the expression levels or activities of these enzymes to modulate sperm sncRNA biogenesis in the epididymis, leading to an altered “sperm RNA code” that affects offspring health (29, 39, 110). Future studies are required to study the detailed mechanism through which consumption of unhealthy diets affects sperm tsRNA/rsRNA biogenesis to mediate intergenerational transmission of paternally acquired CVD and other chronic diseases.

In summary, we investigated the effect of paternal hypercholesterolemia on offspring atherosclerosis development in LDLR^–/–^ mice and revealed that paternal hypercholesterolemia induced sex-specific atherogenic effects in LDLR^–/–^ offspring. Paternal HCD feeding led to significantly increased atherosclerosis in F1 female but not male LDLR^–/–^ mice. The increased atherosclerosis in female offspring also correlated with elevated proatherogenic gene expression in the intima. PANDORA-Seq then uncovered that HCD feeding can lead to altered sperm tsRNA and rsRNA profiles, which could be key contributing factors that convey intergenerational transmission of paternally acquired atherosclerosis in offspring. The altered sperm sncRNA profiles were probably due to HCD-elicited changes in epidydimal RNA modification and cleavage enzymes. Lastly, hypercholesterolemia-stimulated sperm tsRNAs and rsRNAs can induce early transcription changes of cardiovascular development and atherosclerosis-related genes in murine EBs. Our findings are intended to stimulate further investigations of the effect of parental exposures on offspring cardiovascular health and the underlying mechanisms of parentally acquired CVD and other chronic diseases.

## Methods

### Sex as a biological variable.

Our study examined both male and female LDLR^–/–^ mice, and we found there were sex-dimorphic effects for atherosclerosis development in F1 offspring. For F0 mice, only male LDLR^–/–^ mice were exposed to different diets, since the study was designed to investigate the effect of paternal exposures on offspring atherosclerosis development.

### Animals.

Three-week-old littermate male LDLR^–/–^ mice (The Jackson Laboratory) were fed ad libitum on a semisynthetic low-fat (4.2% fat) AIN76 diet containing low cholesterol (LCD; 0.02% cholesterol; Research Diets) or high cholesterol (HCD; 0.5% cholesterol; Research Diets) for 8 weeks before mating with age-matched LCD-fed female LDLR^–/–^ mice (C57BL/6 strain, The Jackson Laboratory) (42–46). Male mice were housed with female mice with free access to LCD during the light cycle. Male mice were returned to their cages overnight with their assigned LCD or HCD, and female mice were maintained on LCD during mating, gestation, and lactation. Female mice were never exposed to HCD. After copulation was confirmed by vaginal plug detection, the male mice were removed from the mating cage and humanely euthanized. These initial mouse pairs are designated F0. The F1 offspring were weaned at 3 weeks of age and were given a LCD until euthanasia at 19 weeks old. To ensure that the atherosclerotic phenotype we observed was not batch specific, 7 LCD-fed sires were mated with 8 control females and 6 HCD-fed sires were mated with 7 control females. Eight litters were generated from LCD-fed sires, and 7 litters were generated from HCD-fed sires. At least 1 mouse from each litter was used for the study. All animals were housed in pathogen-free microisolator cages in a temperature controlled (~21°C) environment with a 12-hour light-dark cycle. On the day of euthanasia, mice were fasted for 6 hours following the dark cycle (feeding cycle). Mice were anesthetized with ketamine/xylazine (100/10 mg/kg BW) by i.p. injection. The peritoneum and chest cavity of the mouse was opened to expose the heart. Blood was collected from the right ventricle of the heart using a 23G needle and 1 mL syringe. Following blood collection, the right atrium was nicked, and 10 mL of saline was injected into the left ventricle to perfuse the circulatory system, which also killed the mouse by exsanguination. The major tissues and organs were collected and weighed as previously described (43, 44, 51, 111).

### Metabolic phenotype analysis.

BW was measured weekly. Body lean and fat mass was measured by NMR spectroscopy (EchoMRI, Echo Medical System). I.p. GTT (IPGTT) was performed as previously described 1 week before sacrifice (33, 44).

### Sperm isolation.

Sperm from F0 mice were collected as previously described (40, 112). Mature sperm was released from the cauda epididymis and incubated in 5 mL of PBS at 37°C for 15 minutes. Afterward, the sperm were filtered through a 40 μm cell strainer to remove any residual tissue debris. Sperm were then incubated in somatic cell lysis buffer (0.1% SDS and 0.5% Triton X-100) for 40 minutes on ice to remove somatic cells. Sperm was pelleted and collected in 1 mL of Trizol (Sigma-Aldrich, T9424) for RNA isolation.

### Blood analysis.

Blood samples were collected from the right ventricle and centrifuged at 1,500*g* for 15 minutes at 4°C. The upper clear phase (serum) was collected for lipid analysis. Serum total cholesterol and total triglyceride concentrations were measured using the Wako Cholesterol E enzymatic colorimetric assay (Wako, 999-02601) and the Wako L-type TG M assay (Wako, 994-02891) according to the manufacturer’s instructions (FUJIFILM Medical Systems). The lipoprotein fractions were isolated in a Beckman Coulter XPN100-IVD ultracentrifuge as previously described (43, 49, 113).

### RNA isolation.

Total RNAs were collected from F0 mouse sperm; testis, caput, and cauda epididymis; F1 mouse intima; and cultured cells as previously described (33, 40, 43, 114). For the intimal RNA isolation, aortas of F1 mice were isolated and flushed with PBS followed by intimal peeling using TRIzol reagent (Sigma-Aldrich, T9424). A total of ~300–400 μL of Trizol was flushed into the aorta for 10 seconds (~100 μL) followed by a 10-second pause 3 times as previously described (43). The flowthrough was collected in a 1.5 mL Eppendorf tube followed by RNA extraction.

### Atherosclerotic lesion analysis.

The atherosclerotic plaque sizes were quantified as previously described (112, 115, 116). To quantify the plaque area at the aortic root, optimal cutting temperature (OCT) compound–embedded hearts were sectioned at a 12 μm thickness, keeping all the 3 valves of the aortic root in the same plane, and stained with Oil Red O. To quantify the atherosclerotic plaque area at the BCA, the OCT-embedded BCAs were sectioned from distal to proximal at a thickness of 10 μm. BCA atherosclerotic lesions from the lumenal to the internal elastic lamina were quantified in 3 equidistant Oil Red O–stained sections at 200, 400, and 600 μm proximal from the branching point of the BCA into the carotid and subclavian arteries. Images were taken and plaque size was quantified using a Nikon microscope (Nikon).

### Cell culture.

Human endothelial cell line HMEC-1 was purchased from ATCC (catalog CRL-3243) (43). Cells were treated with 1 μg/mL CCN1 or CCN2 for 1, 3, or 4 hours and then used for the indicated quantitative PCR (qPCR) and Wester blotting experiments. For macrophage adhesion assay, HMEC-1 cells were pretreated with 10 ng/mL TNF-α or with 1 μg/mL CCN1 or CCN2 for 24 hours. LDLR^–/–^ mice were injected with 1 mL of thioglycolate for 3 days to stimulate macrophage production. Isolated peritoneal macrophages were stained with calcein acetoxymethyl and incubated with HMEC-1 for 4 hours. The attached macrophages were fixed and counted under a fluorescence microscope. mESCs containing an Oct4-GFP reporter were provided by Sihem Cheloufi (University of California, Riverside). mESCs were maintained in stem cell media and passaged every 2 days in gelatin-coated dishes as we previously described (40).

### mESC transfection and EB formation assay.

mESC transfection and EB formation assays were performed as we previously described (40). mESCs were transfected with vehicle, control oligos, and tsRNA/rsRNA pool oligos for EB differentiation assay. After 24 hours, the EBs were collected and total RNAs were extracted for further analysis. For each transfection, 3 independent replicates were performed. Vehicle and control oligos were used as controls. The tsRNA/rsRNA pool oligo contained the following RNAs: tsRNA Glu-CTC/TTC (5′-ACCGCCGCGGCCCGGGTTCGTTTCCCGGTCAGGGAAC-3′), mt-tsRNA His-GTG (5′-GGTGAATATAGTTTACAAAAAACATTAGACTGTGAATCTGACAA-3′), rsRNA 18S (5′-TGGATCTTGGGAGCGGGCGGGCGGTCCGCCGCGAGGCGA-3′), and rsRNA 28S (5′-CGCGACCTCAGATCAGACGTGGCGACCCGCTGAATTTAAGCAT-3′ and 5′-TCCTTCTGATCGAGGCCCAGCCCGTGGACGGTGTGAGGCCG-3′).

### qPCR.

We measured the relative mRNA expression levels by qPCR with the SYBR Green (Bio-Rad, 170–8886) kit using a Bio-Rad CFX Real-Time-PCR Machine (Bio-Rad, 184–5096) (33, 43, 114). The primer sequences are included in Supplemental Table 4.

### Western blotting.

Western blotting experiments were performed as previously described (33, 111, 113). Primary antibodies including anti-actin (1:5,000 dilution, MilliporeSigma A2066), anti-p65 (1:1,000 dilution, Cell Signaling Technology, 3034), and anti–phospho-p65 (Ser 536) (1:1,000 dilution, Cell Signaling Technology, 93H1) as well as anti–rabbit secondary antibodies (1:5,000 dilution, MilliporeSigma, 12–348) were used for these experiments.

### Immunofluorescence staining.

The cryosections of mouse aortic root and cultured human HMEC-1 cells were used for immunofluorescence staining as previously described (43, 115). For immunostaining, samples were incubated with antibodies against MOMA2 (Bio-Rad, MCA519a), MCP-1 (Abcam, ab7202), IL-6 (Bio-Rad AbD Serotec, MCA1490), α-SMA (Abcam, ab5694), CCN1 (R&D, 4055CR050), CCN2 (R&D, 9190CC050), or p65 (Santa Cruz Biotechnology, sc-372) at 4°C for 12–15 hours. The sections were rinsed with PBS and incubated with fluorescein-labeled secondary antibodies (Invitrogen). The nuclei were stained by mounting the slides with DAPI medium (Vector Laboratories).

### RNA-Seq and transcriptomic data analysis.

The creation of cDNA libraries and sequencing were performed using the Illumina standard operation pipeline as previously described (33, 51, 117, 118). For data analysis, we applied the *Salmon* tool to quantify the mRNA expression from the raw sequencing data with the default setting, based on the Ensembl mouse cDNA annotation (GRCm38). We then employed the *edgeR* algorithm (119) to perform the groupwise comparison in transcriptomic pattern, using the *TMM* algorithm to perform read count normalization and effective library size estimation and the likelihood ratio test to identify the DEGs. The genes with a FDR < 0.1 and a fold change (FC) > 1.5 was deemed differentially expressed. We further performed GO analysis upon the DEGs using the definition from GO project. The DAVID bioinformatics tool (120) was applied to detect the GOBP terms enriched by the DEGs. For each prioritized GOBP term, we computed a gene set score, using the FAIME algorithm (121). A higher FAIME score suggests an increased overall expression of a given GOBP term/gene set. All the RNA-Seq data sets have been deposited in the Gene Expression Omnibus (GEO; GSE251713).

### PANDORA-Seq of sperm small RNAs.

PANDORA-Seq protocol has been described in detail in our recent reports (33, 40, 43). Briefly, sperm total RNAs isolated for LCD- and HCD-fed male LDLR^–/–^ mice were run through a 15% urea polyacrylamide gel. Small RNA of 15–50 nucleotides was visualized with SYBR Gold solution (Invitrogen, S11494) and excised (40). A sample of the eluted RNA was stored in –80°C for Traditional-Seq. The remaining RNA was eluted and then treated with T4PNK reaction mixture (5 μL 10× PNK buffer, 1 mM ATP, 10 U T4PNK) followed by RNA isolation with TRIzol. The collected RNAs were then treated with AlkB mixture (50 mM HEPES, 75 μM ferrous ammonium sulfate, 1 mM α-ketoglutaric acid, 2 mM sodium ascorbate, 50 mg/L BSA, 4 μg/mL AlkB, 2,000 U/mL RNase inhibitor) followed by RNA isolation with TRIzol. The recombinant AlkB enzyme was prepared by Linlin Zhao (University of California, Riverside) as previously described (40). The adapters (New England Biolabs, E7330S) were ligated sequentially (3′ adapter, revers transcription primer, 5′ adapter). First-strand cDNA synthesis was performed followed by PCR amplification with PCR Primer Cocktail and PCR Master Mix to enrich the cDNA fragments. Finally, the PCR products were purified from PAGE gel and prepared for sequencing at the Genomics Center of UCSD (San Diego, California, USA) (Illumina system) (33, 40, 43).

Small RNA-Seq results were annotated using the software *SPORTS1.1* with one mismatch tolerance (*SPORTS1.1* parameter setting: -M 1). Statistical significance among different groups was determined by 2-sided 1-way ANOVA with uncorrected Fisher’s least significant difference test. Pairwise comparison of differentially expressed sncRNAs (average raw counts of each sncRNA > 10 in the compared treatments) among different diets was performed using the R package DEseq2 with a normalized RPM FC > 2 and *P* < 0.05. All the small RNA-Seq data sets have been deposited in the GEO (GSE251713).

### Statistics.

All data except the high-throughput sequencing data are presented as the mean ± SEM. Individual pairwise comparisons were analyzed by 2-sample, 2-tailed Student’s *t* test. One-way ANOVA was used for analyzing different origins of the tsRNA/miRNA expression ratio under different treatments (uncorrected Fisher’s LSD test) or when the statistical significance of more than 2 groups were analyzed (Bonferroni’s multiple-comparison test). Two-way ANOVA was used when multiple comparisons were made, followed by a Bonferroni multiple comparisons test. Data analysis was performed using the GraphPad Prism 10 software with statistical significance set at *P* < 0.05.

### Study approval.

All animal studies were performed in compliance with the IACUC protocols approved by the University of California, Riverside.

### Data availability.

All the RNA-Seq data sets have been deposited in the GEO under the accession no. GSE251713. Values for graphs in the figures and supplemental figures are provided in the Supporting Data Values file.

## Author contributions

CZ and RH conceptualized and designed the research. RH performed most of the experiments and analyzed the data with the help from XL, JS, TRD, TZ, and QC. RH, TZ, QC, and CZ wrote the manuscript.

## Supplementary Material

Supplemental data

Unedited blot and gel images

Supporting data values

## Version 1 09/10/2024

Electronic publication
