# Supplementary material for: Paternal hypercholesterolemia elicits sex-specific exacerbation of atherosclerosis in offspring
Source: JCI Insight. 2024 Sep 10;9(17):e179291. doi: 10.1172/jci.insight.179291 (PMC11385100; doi:10.1172/jci.insight.179291)
Supplement: Supplemental data [file jciinsight-9-179291-s012.pdf]

Supplemental Material for

Paternal hypercholesterolemia elicits sex-specific exacerbation of atherosclerosis in offspring

Rebecca Hernandez,[1] Xiuchun Li,[1] Junchao Shi,[1,2] Tejasvi Dave[1], Tong Zhou,[3] Qi Chen,[2] and Changcheng Zhou[1]

[1]Division of Biomedical Sciences, School of Medicine, University of California, Riverside, California, USA
[2] Molecular Medicine Program, Department of Human Genetics, and Division of Urology, Department of Surgery, University of Utah School of Medicine, Salt Lake City, Utah, USA
[3]Department of Physiology and Cell Biology, University of Nevada, Reno School of Medicine, Reno, Nevada, USA

This PDF file includes:

Supplemental Figure 1-7
Supplemental Table 1-3

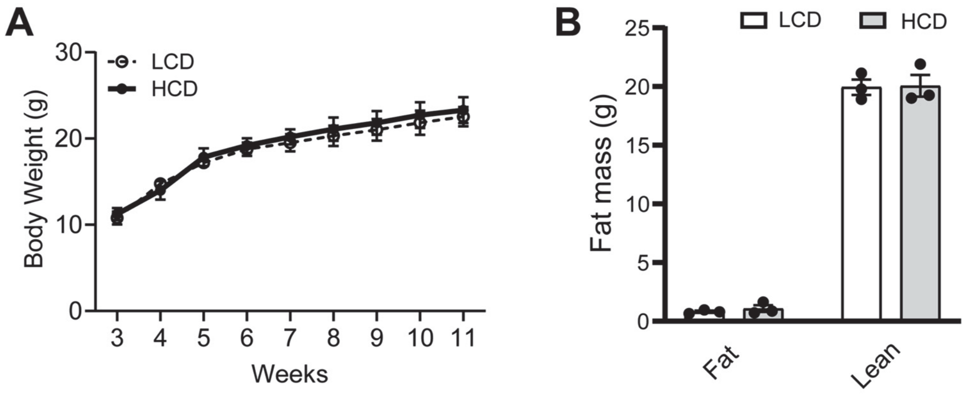

Supplemental Figure 1. High-cholesterol diet feeding does not affect body weight and adiposity in LDLR$^{-/-}$ mice.
Three-week-old male LDLR$^{-/-}$ mice were fed an LCD or HCD for 9 weeks. (A) Growth curves were measured (n=5-6, two-way *ANOVA* followed by Bonferroni's multiple comparison test). (B) Body composition of LDLR$^{-/-}$ mice fed an LCD or HCD were measured (n=3, two-sample, two tailed Student's t-test). All data are plotted as means ± S.E.M.

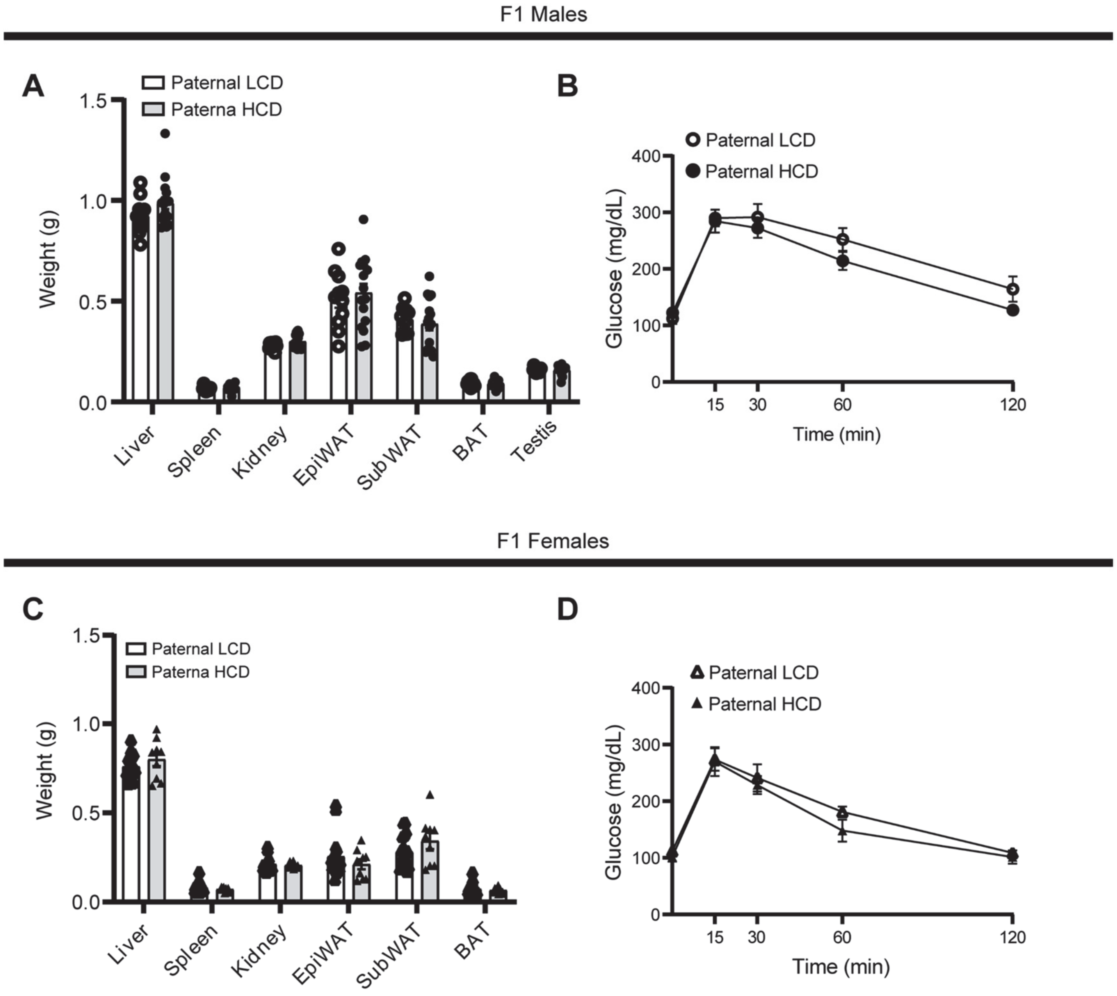

Supplemental Figure 2. Paternal hypercholesterolemia does not affect organ weight or glucose tolerance tests in F1 LDLR$^{-/-}$ offspring.
Three-week-old male LDLR$^{-/-}$ mice were fed an LCD or HCD diet for 8 weeks before mating with female LDLR$^{-/-}$ mice. Three-week-old F1 offspring were fed an LCD for 16 weeks and euthanized at 19 weeks of age. Major organ weights (A and C) of male and female offspring were measured (n=9-18, *P<0.05, two-sample, two tailed Student's t-test). (B and D) Intraperitoneal glucose tolerance test (IPGTT) of male and female offspring was performed (n=8-10, two-way *ANOVA* followed by Bonferroni's multiple comparison test). All data are plotted as means ± S.E.M.

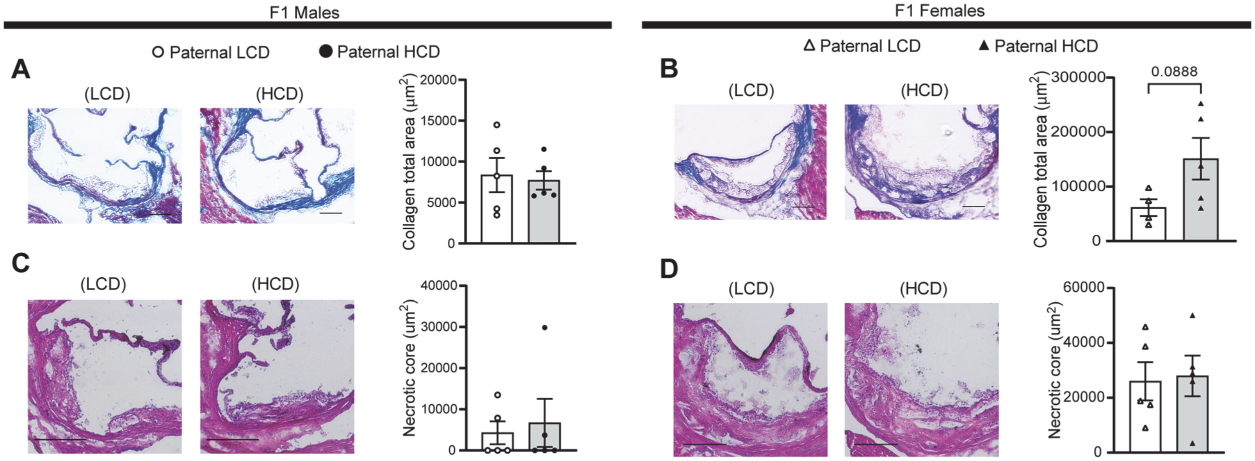

Supplemental Figure 3. Paternal high cholesterol diet does not affect collagen production or necrotic core formation in the aortic root of F1 offspring.

Three-week-old male C57BL/6 LDLR$^{-/-}$ littermates were fed an LCD or HCD diet for 8 weeks before mating with female LDLR$^{-/-}$ mice. Three-week-old F1 offspring were fed a LCD for 16 weeks and euthanized at 19-weeks of age. (A and B) Masson's Trichrome stain was used to measure the collagen content at the aortic root of male (A) and female (B) offspring. Representative images are shown to the left of each data set (scale bar=100μm). (C and D) Hematoxylin and eosin staining was used for analyzing the necrotic core at the aortic root of male (C) and female (D) offspring. Representative images are shown to the left of each data set (scale bar=200μm). Statistical significance was determined by independent t-test. All data are plotted as means ± S.E.M. (n=4-5).

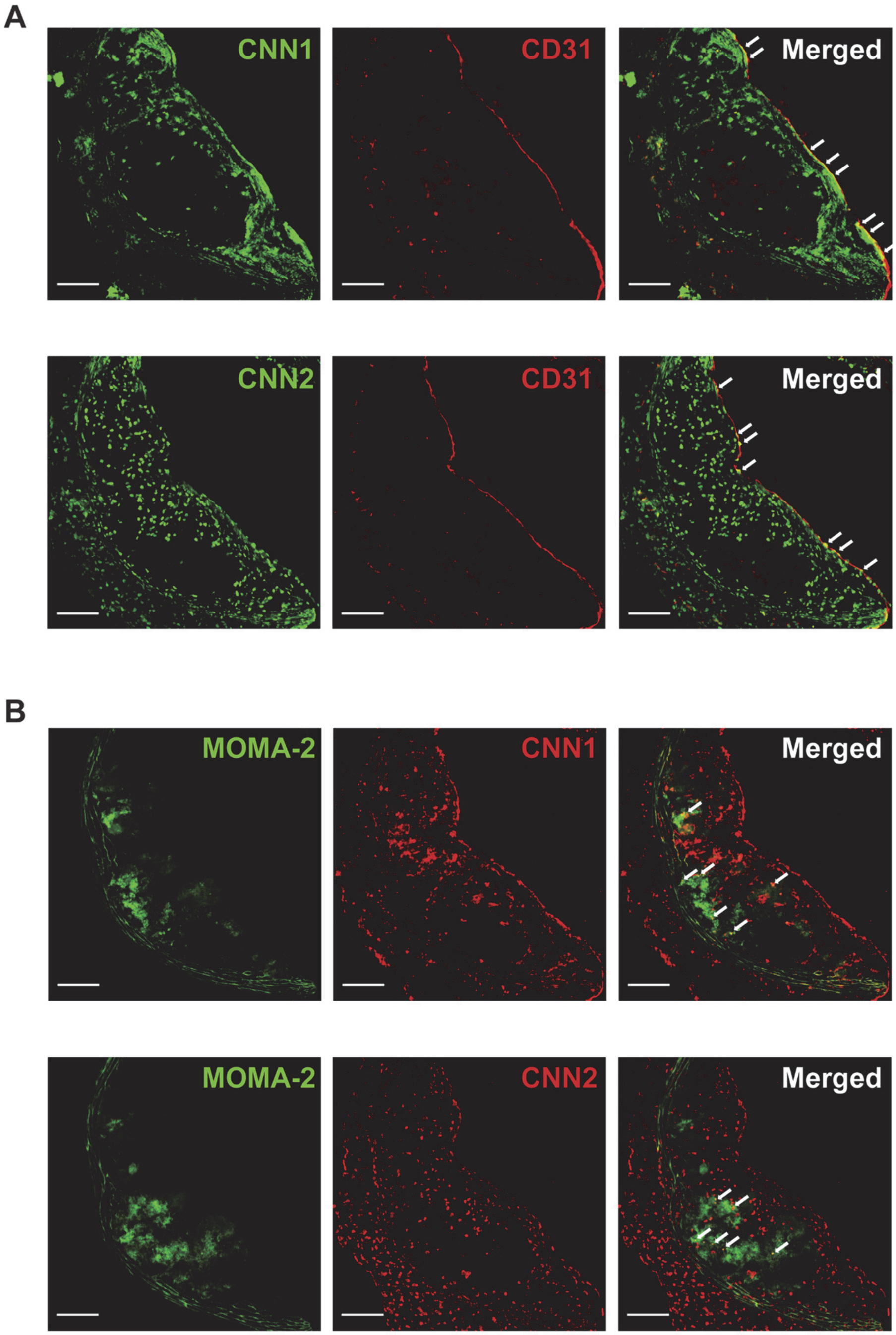

Supplemental Figure 4. CCN proteins are co-localized with macrophage and endothelial cells in atherosclerotic lesions.

(A) Representative images of CD31 and CCN1 or CCN2 immunofluorescent staining in the aortic root of LDLR$^{-/-}$ mice (scale bar=100μm). (B) Representative images of 3 experimental replicates for MOMA-2 and CCN1 or CCN2 immunofluorescent staining in the aortic root of LDLR$^{-/-}$ mice (scale bar=100μm).

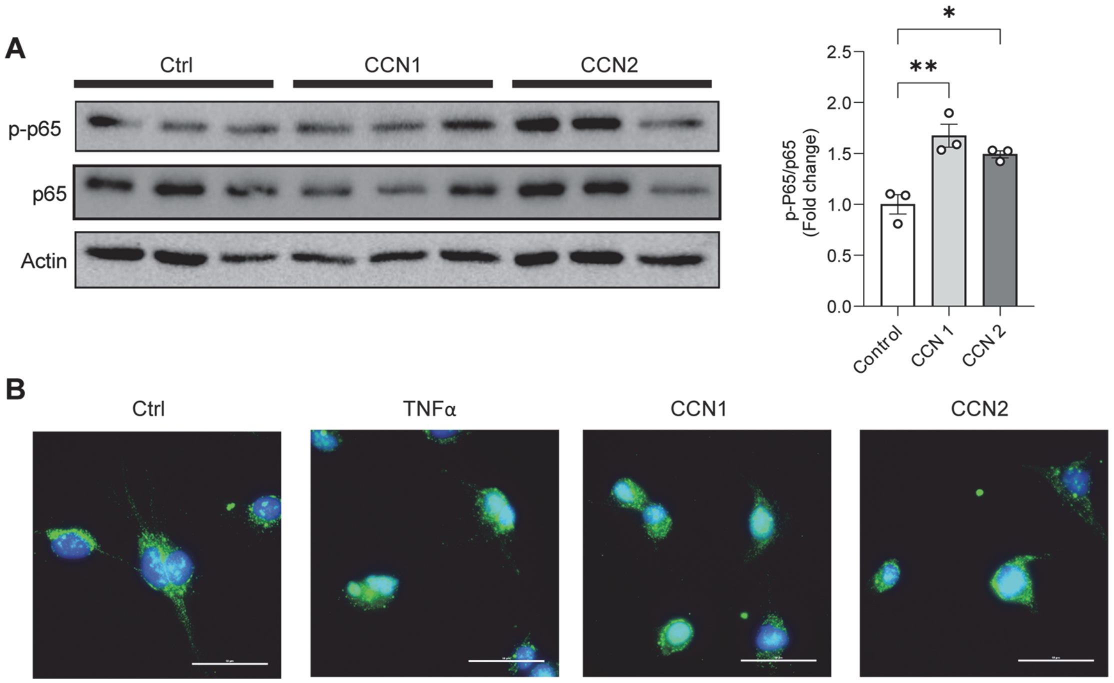

Supplemental Figure 5. CCN proteins stimulate NF-κB activation in human endothelial cells. (A) Human endothelial cells, HMEC-1 cells, were treated with 1 μg/ml CCN1 or CCN2 for 1 hour followed by protein extraction. Immunoblotting of phosphorylated NF-κB subunit p65, total 65, and Actin (left) and quantification data (right) (n=3, *P<0.05, *P<0.05, one-way *ANOVA* followed by Bonferroni's multiple comparison test). (B) Representative immunofluorescence staining images of 3 experimental replicates for NF-κB p65 (green) in HMEC-1 cells treated with vehicle control or 1 μg/ml CCN1 or CCN2 for 3 hours. The nuclei were stained with DAPI (blue). (scale bar= 50μm). All data are plotted as means ± S.E.M.

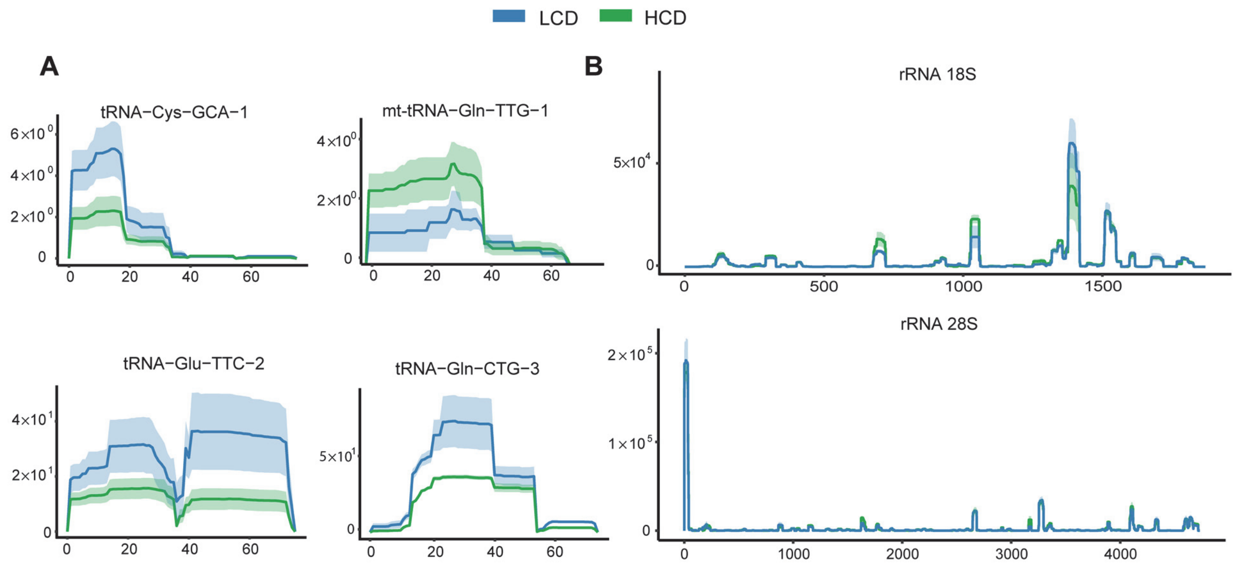

Supplemental Figure 6. PADNORA-seq reveals dynamic changes to high-cholesterol diet feeding of representative sperm tsRNAs and rsRNAs.
Three-week-old male $LDLR^{-/-}$ mice were fed an LCD or HCD for 9 weeks. Total RNAs were isolated from the sperm and used for PANDORA-seq. (A-B) Dynamic responses to the LCD or HCD of representative individual sperm tsRNAs (A) and sperm rsRNAs (B) detected by PADNORA-seq. Mapping plots are presented as mean ± SEM (n = 3 in each group).

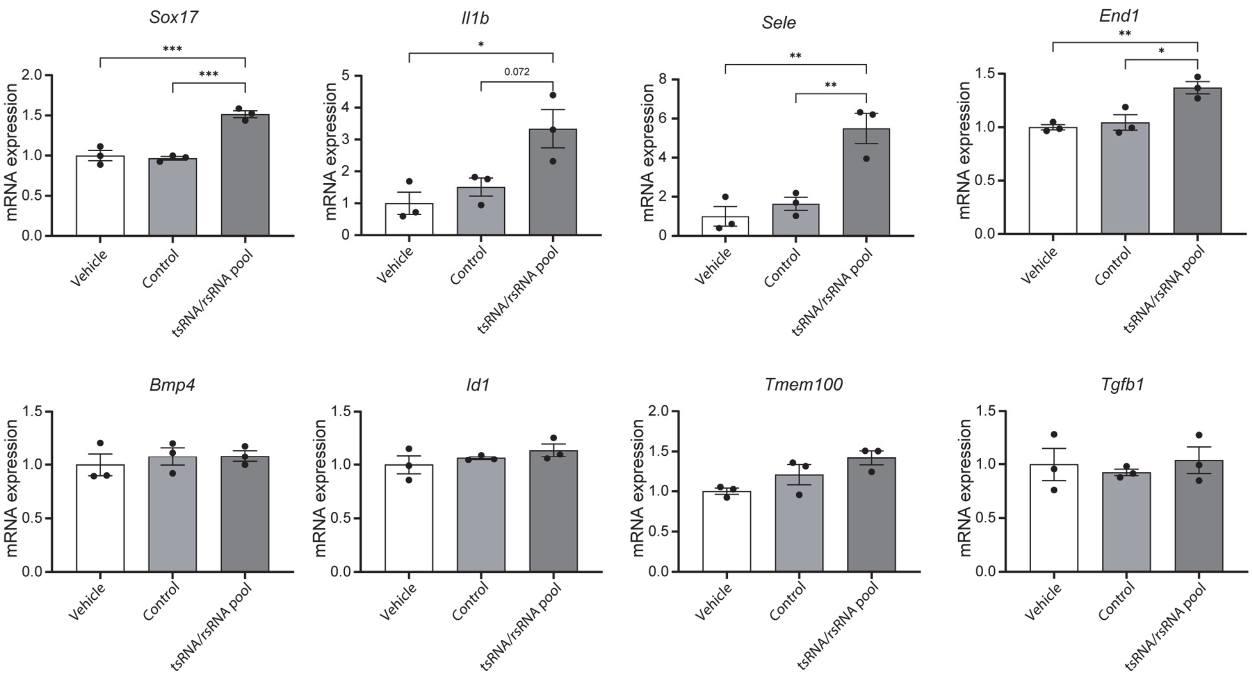

Supplemental Figure 7. Hypercholesterolemia-stimulated sperm tsRNAs/rsRNAs induce early transcription changes in murine embryoid bodies.

Murine embryonic stem cells were transfected with a pool of tsRNA/rsRNAs, control oligo or vehicle followed by 24 hours of embryoid body (EB) formation assay. Total RNA was isolated from EBs for gene expression analysis. The expression levels of indicated genes were analyzed by quantitative real-time PCR (n=3, *P<0.05, **P<0.01). Statistical significance was determined by one-way ANOVA followed by Bonferroni's multiple comparison test. All data are plotted as means ± S.E.M.

Supplemental Table 1. Detailed RNA-seq data of intimal DEGs induced by paternal HCD feeding in F1 male LDLR$^{-/-}$ mice

Gene	log2 (FC)	P-value	FDR	FC	Gene	log2 (FC)	P-value	FDR	FC
Ppm1n	8.031722239	0.000437032	0.069087641	261.6913	Islr	-1.183308958	0.000305797	0.057657344	0.431904
Mup7	6.93620077	0.000518341	0.075430815	122.4629	Nexn	-1.211218886	0.000845023	0.095430365	0.429626
Olfr731	6.497106881	2.89304E-05	0.017424912	90.32835	Col6a2	-1.21884639	0.000121527	0.03981086	0.428694
Myh1	5.559119457	0.000199743	0.050376497	47.14783	Fzd2	-1.221980808	0.000279886	0.055152134	0.423375
Dusp13	5.447649862	0.000280667	0.055152134	43.64214	Ms4a4d	-1.239991122	0.000661709	0.083512139	0.421398
Il6	5.254781324	3.97313E-05	0.019895097	38.18096	Ano1	-1.246745267	0.000403924	0.067183507	0.419896
Bcan	5.215276802	0.000661513	0.083512139	37.14965	Serpine2	-1.251896623	0.000123015	0.03981086	0.418756
Mab21l3	5.04002662	0.000666233	0.083512139	32.90025	Rcan2	-1.255819099	1.59568E-05	0.015364023	0.413882
Ldlrad1	4.940193852	0.000872211	0.097485278	30.70058	Akap12	-1.272707128	0.000282336	0.055152134	0.412841
Tpm3-rs7	4.636642279	0.000502501	0.073619722	24.8753	Gria4	-1.276340421	0.000294793	0.056070081	0.408718
Vmn1r73	4.475230886	0.000753086	0.088745451	22.24225	Mark1	-1.290821309	0.000258757	0.054472117	0.407925
Fosb	4.164003971	2.39326E-05	0.016771729	17.92628	Hmgcs2	-1.293624662	0.000281839	0.055152134	0.406516
Rspo2	3.627875217	0.00063485	0.083426988	12.3623	Mrvi1	-1.298614378	0.000246243	0.054265918	0.402605
Egr1	2.583294077	0.000349608	0.061133504	5.993065	F13a1	-1.312563207	0.000119326	0.03981086	0.402533
Fos	2.541358626	0.000143499	0.043380327	5.82137	Tent5b	-1.312820949	0.000149805	0.043894986	0.402099
Socs3	2.536426146	3.39487E-05	0.018874634	5.801501	Fmo3	-1.314377057	6.44109E-06	0.008215418	0.40114
Cxcl1	2.016171098	0.000599181	0.081750448	4.045088	Thsd4	-1.317822738	0.000338096	0.060089694	0.400061
H2ac6	1.826818135	0.000271592	0.055036622	3.547538	Lgr6	-1.321708375	6.92187E-06	0.008227875	0.399927
Eps8l1	1.575066609	0.000896725	0.098698943	2.979492	Brd3os	-1.323098003	0.000568185	0.079483634	0.399676
Gadd45b	1.190810079	0.000154217	0.044585195	2.282809	Slc24a3	-1.328412167	6.36869E-05	0.02761846	0.398206
Plk3	1.138785961	0.000702001	0.085513973	2.201956	Sec16b	-1.33423745	0.000792813	0.092832959	0.396602
Sox7	1.126276399	0.000889665	0.098421442	2.182946	Gucy1b1	-1.33614819	0.000374694	0.063972363	0.396077
Micall2	0.956059065	2.70765E-05	0.017424912	1.940003	Cd163	-1.342369462	0.000796335	0.092832959	0.394372
Smad6	0.90873493	0.000911852	0.098918842	1.877399	Fbln1	-1.342675179	0.000716601	0.086322564	0.394289
Pim1	0.885693755	0.000742437	0.088451927	1.847653	Plekhh2	-1.351740403	0.000693874	0.085335567	0.391819
Nt5e	0.778248835	0.000549323	0.077849498	1.715048	Gabra3	-1.355973843	3.60956E-05	0.019566527	0.390671
Cadm1	0.745583817	0.000136698	0.042576856	1.676653	Dtna	-1.361343626	6.13684E-05	0.02761846	0.38922
Arl4c	0.69423731	0.000174582	0.047624487	1.618029	Rbm24	-1.365103912	0.000842291	0.095430365	0.388206
Nova1	0.686111871	0.000649243	0.083512139	1.608941	Gli1	-1.371134371	0.000149179	0.043894986	0.386587
Exoc6	0.654272716	0.000723408	0.08666108	1.573822	Ldb3	-1.37744501	0.000537726	0.076707366	0.3849
Prkce	0.632445805	0.000399016	0.067068721	1.550191	Abcc9	-1.382811676	0.000332723	0.059623346	0.383471
Slc46a3	0.625453293	0.000168443	0.047291574	1.542695	Sh3bgr	-1.385991247	6.61058E-07	0.002047675	0.382627
Adgre5	0.620944872	0.000271484	0.055036622	1.537882	Susd5	-1.386696526	0.000535237	0.076707366	0.38244
Lgalsl	-0.595786994	0.000630641	0.083379179	0.661683	Epha3	-1.386935351	0.00062363	0.083186515	0.382376
Smarcd3	-0.596622964	0.000362473	0.062876043	0.6613	Dmpk	-1.390703226	4.43361E-06	0.006408929	0.381379
Itgbl1	-0.621599556	0.00059526	0.081750448	0.64995	Scube3	-1.403914798	0.000692737	0.085335567	0.377902
Col4a5	-0.631624619	0.000115263	0.03981086	0.645449	Dsp	-1.404824307	5.39495E-07	0.002047675	0.377664
Cercam	-0.642652306	0.000164239	0.04685788	0.640534	Ncald	-1.413533838	0.000817014	0.0944789	0.375391
Serpinh1	-0.646872065	0.000819169	0.0944789	0.638664	Stum	-1.437786654	4.03719E-05	0.019895097	0.369133
Twsg1	-0.668798654	0.000594238	0.081750448	0.62903	Postn	-1.438783302	0.000426565	0.069023926	0.368878
Tspan11	-0.728331586	0.000603096	0.081750448	0.603602	Col16a1	-1.469359186	0.000692026	0.085335567	0.361143
Ift43	-0.730888683	4.38286E-05	0.021118556	0.602533	Jph2	-1.485687798	0.000241563	0.053998044	0.357078
Ndrg2	-0.731063746	0.000437502	0.069087641	0.60246	Kcnmb1	-1.493455181	7.40734E-05	0.030304404	0.355161
Nkd1	-0.736711068	0.000290196	0.055684196	0.600106	Myh11	-1.494171034	2.07406E-05	0.016061401	0.354985
Synpo2	-0.750401469	0.00066631	0.083512139	0.594438	Adamtsl2	-1.50718569	0.000247767	0.054265918	0.351797
Cbr2	-0.772823822	2.23838E-06	0.004412249	0.585271	Nox4	-1.509113938	1.50928E-05	0.015364023	0.351327
Jam3	-0.774276521	7.00905E-05	0.029799438	0.584682	Cap2	-1.526800546	0.000527529	0.076256117	0.347046
Arhgef17	-0.782973123	0.000194041	0.050376497	0.581168	Fstl3	-1.550734754	2.35737E-05	0.016771729	0.341336
Col4a6	-0.795788843	3.95047E-05	0.019895097	0.576028	Cldn10	-1.553025711	0.000928491	0.099665662	0.340795
Papss2	-0.803700073	1.89657E-06	0.004112342	0.572878	Ntf3	-1.553242331	8.96962E-06	0.009724419	0.340743
Il33	-0.813819868	5.55007E-06	0.007521389	0.568874	Itga8	-1.57375689	0.000220567	0.052405482	0.335932
Tead3	-0.818153815	0.000425299	0.069023926	0.567167	Prss12	-1.598586	0.00056493	0.079483634	0.3302
Sema5a	-0.824626071	0.000825541	0.094710139	0.564629	Clmp	-1.601280721	0.000105333	0.037441518	0.329584
Mylk	-0.851025007	3.57384E-06	0.006007331	0.554391	Filip1	-1.611073001	0.000250508	0.054317667	0.327355
Vasn	-0.852856604	1.57288E-07	0.002047675	0.553687	Sgcg	-1.679981444	1.82682E-05	0.015364023	0.312087
Wfdc1	-0.887510008	7.20978E-06	0.008227875	0.540546	Fbxo40	-1.687762406	0.000221862	0.052405482	0.310408
Bcl6	-0.891017556	1.20311E-06	0.002898561	0.539234	Mst1r	-1.689093439	0.000224769	0.052405482	0.310122
Osr1	-0.897578733	2.59437E-05	0.017424912	0.536787	Sync	-1.693337888	7.29241E-05	0.030304404	0.309211
Nbl1	-0.898290413	0.000189774	0.050181368	0.536522	Fibin	-1.699699709	4.35089E-07	0.002047675	0.30785
St5	-0.90493589	1.70653E-05	0.015364023	0.534056	Ngef	-1.750458197	0.000396503	0.067068721	0.297207
Sema3d	-0.915736279	9.75752E-07	0.002644654	0.530073	Bmpr1b	-1.754105576	7.68223E-05	0.030846998	0.296457
Enah	-0.915873599	0.000642699	0.083512139	0.530023	Col15a1	-1.757757099	2.03611E-07	0.002047675	0.295708
Cfb	-0.922848931	5.7393E-05	0.026611807	0.527466	Tub	-1.761767169	0.000471684	0.070769966	0.294887
Aldh1a1	-0.934698547	0.000332522	0.059623346	0.523152	Mylk4	-1.800942192	3.59771E-07	0.002047675	0.286987
Tes	-0.943020942	1.84229E-05	0.015364023	0.520143	Cacnb2	-1.804141053	0.000262143	0.054654351	0.286351
Igfbp7	-0.964216138	0.000663064	0.083512139	0.512557	Matn2	-1.804615149	0.000177908	0.047624487	0.286257
Sparcl1	-0.964877087	6.18076E-07	0.002047675	0.512322	Sh3rf2	-1.808183221	0.000175938	0.047624487	0.28555
Tgfb3	-0.977256496	0.000446008	0.069574032	0.507945	Kcnab1	-1.827919106	0.000321748	0.059122523	0.281671
Efhd1	-0.982461908	0.000170121	0.047291574	0.506115	Kcnma1	-1.842176544	0.000125273	0.039945545	0.278901
Rarres2	-0.986598723	0.000371969	0.063972363	0.504666	Gipc2	-1.879541166	0.000208817	0.050873859	0.27177
Ramp1	-0.994161306	0.000606304	0.081750448	0.502028	Fbxo2	-1.931527294	2.8081E-05	0.017424912	0.262152
Arhgef25	-1.003005891	6.26483E-05	0.02761846	0.498959	Sv2a	-2.066383715	0.000103675	0.037441518	0.238757
Atf5	-1.015353033	0.000466278	0.070769966	0.494707	Angptl7	-2.107137375	0.000325444	0.059299203	0.232107
Cacna2d1	-1.024826784	0.000607011	0.081750448	0.491469	Casq1	-2.124445129	3.35621E-05	0.018874634	0.229339
Srpx	-1.028597883	0.000119568	0.03981086	0.490186	Tceal6	-2.140516477	3.60168E-06	0.006007331	0.226799
Sept5	-1.028788479	2.39784E-05	0.016771729	0.490122	Msc	-2.794469316	0.000137452	0.042576856	0.144139
Ppp1r12b	-1.029258105	3.70149E-05	0.019575486	0.489962	Lrrc75b	-2.797037752	0.000436485	0.069087641	0.143882

Sost	-1.039567918	7.83283E-05	0.030879882	0.486473	Cdh18	-3.021646843	0.000696601	0.085335567	0.123138
Npnt	-1.048669793	9.40843E-05	0.034576782	0.483414	Cyp4f14	-3.298312419	0.000439704	0.069087641	0.10165
Csrp2	-1.050509557	2.06113E-05	0.016061401	0.482798	Gm11541	-3.70752512	0.00022991	0.05303341	0.076546
Galnt16	-1.051229782	0.000204389	0.050873859	0.482557	Gm11639	-3.810824907	0.000348391	0.061133504	0.071257
Abca9	-1.064130002	0.000457994	0.070430363	0.478261	Krt14	-4.159706848	0.000317666	0.058871376	0.05595
Gpsm2	-1.065436833	0.000712776	0.086322564	0.477828	Pklr	-4.803371594	0.000199805	0.050376497	0.035813
Npy1r	-1.082881735	5.76837E-05	0.026611807	0.472085	Epcam	-4.851266639	0.000405896	0.067183507	0.034644
Pde5a	-1.085785602	9.04028E-05	0.033796628	0.471136	Scn10a	-4.88921117	0.000285148	0.055204239	0.033744
Medag	-1.088141954	0.000491782	0.072539467	0.470367	Onecut2	-5.505000307	8.70236E-05	0.033104073	0.022021
Dact3	-1.088811299	0.000199447	0.050376497	0.470149	Pkp3	-5.538616874	8.20688E-05	0.031776763	0.021513
Sema3c	-1.104926563	0.000625347	0.083186515	0.464926	Ly6g2	-5.584506795	0.000473258	0.070769966	0.02084
Heyl	-1.119539421	1.70411E-05	0.015364023	0.460241	Pcdha9	-5.660012976	0.000652057	0.083512139	0.019777
C2	-1.12131389	0.000455317	0.070430363	0.459675	Ttr	-5.743751963	0.000144048	0.043380327	0.018662
Olfml2b	-1.13569966	0.000469157	0.070769966	0.455114	Cabp4	-6.131594306	0.000313219	0.058547714	0.014263
Dkk3	-1.137513427	4.17854E-06	0.006408929	0.454542	Cblc	-6.302650195	0.000268668	0.055036622	0.012668
Prelp	-1.145158156	0.000916971	0.098918842	0.45214	Cpn1	-6.394426027	0.000485001	0.072029363	0.011887
Fgf1	-1.158406746	3.05647E-05	0.017911711	0.448007	Gdf1	-7.189480262	2.86689E-05	0.017424912	0.006851
Olfml1	-1.165365387	0.00091082	0.098918842	0.445851	Akr1b7	-7.795129597	0.000120996	0.03981086	0.004502
Rgs7bp	-1.166154371	0.000257483	0.054472117	0.445608	Aoc1	-8.025595618	0.000750326	0.088745451	0.003838
Igfbp6	-1.168456479	0.000417127	0.068519483	0.444897	Mgat4c	-8.442351457	0.000838721	0.095430365	0.002875
Aspn	-1.322192342	0.000254462	0.054472117	0.441381	Crisp1	-9.069918908	0.000224771	0.052405482	0.001861
Htra3	-1.179904463	0.000853378	0.095874608	0.44034					

Supplemental Table 2. Detailed RNA-seq data of intimal DEGs induced by paternal HCD feeding in F1 female LDLR$^{-/-}$ mice

Gene	log2 (FC)	P-value	FDR	FC	Gene	log2 (FC)	P-value	FDR	FC
Otop1	8.814022723	0.00016446	0.045782214	450.0753441	Fcer1g	0.941486608	1.53919E-05	0.018073585	1.920506182
Hist1h2ap	5.932732553	0.000364659	0.066444582	61.08442003	Lrrc25	0.938580645	0.000140936	0.041861881	1.916641677
Gm7233	5.408642117	6.75498E-05	0.034873408	42.47794644	Evi2b	0.933430588	3.15694E-05	0.028521597	1.909811947
Tmprss3	5.171289901	0.000279492	0.059413936	36.03407487	Coro1a	0.929436253	5.26138E-05	0.031448092	1.904531636
Axdnd1	4.790355893	1.92153E-05	0.02083224	27.67201687	Spi1	0.928419726	0.000112114	0.037983976	1.903190171
D7Ertd443e	4.69969579	0.000476106	0.075474474	25.98659651	Cxcl16	0.925479659	0.000138853	0.041861881	1.899315611
Nps	4.476442521	0.000501548	0.076049463	22.26093865	Rnf149	0.923996186	0.000438393	0.074565456	1.897363615
Slco4c1	3.891207736	0.000617368	0.086373158	14.83782511	Bin2	0.920916851	0.000559181	0.081734682	1.893318139
Erich6	3.727604239	0.000181859	0.048088332	13.24709613	Evi2a	0.920464249	0.00017175	0.04655064	1.89272426
Spaca9	3.499097137	4.64935E-05	0.031448092	11.3066304	Gatm	0.915902505	0.000308673	0.061403285	1.886748994
Lrr1	3.385946612	0.000707963	0.091373544	10.45373517	Cd53	0.9147479	0.000166151	0.045782214	1.885239612
Ccn1	2.413898377	0.000240566	0.056932649	5.329123906	Apoe	0.909719617	0.000774821	0.098632164	1.878680348
Sytl3	2.40457603	0.000497054	0.075898797	5.29479941	Ncf4	0.908214424	0.000155325	0.044314757	1.876721304
Igfbp2	2.200582039	4.07062E-12	8.82633E-08	4.596647511	Ly86	0.90280727	0.000114411	0.038075161	1.869700605
Thbs1	2.001286144	4.95477E-05	0.031448092	4.003567539	Ccr5	0.900905018	7.86533E-05	0.035529994	1.867236954
Npy	1.888606974	0.000117456	0.038075161	3.702775225	Milr1	0.893617731	0.000424139	0.072989005	1.857829017
Rgs1	1.843967418	6.31906E-07	0.001957374	3.589959124	Lpxn	0.889798363	9.94457E-05	0.037812439	1.852917134
Edn1	1.819222498	0.00025637	0.056932649	3.52890966	Fyb	0.887962033	0.000545004	0.081498755	1.850560155
Col6a4	1.801391876	0.00070097	0.091012742	3.485563416	C1qb	0.883649534	0.000486507	0.075474474	1.845036726
Arl5c	1.726177667	2.39837E-05	0.023099773	3.30850089	Ptpn6	0.880146215	0.000382501	0.067981695	1.840561831
Hs6st2	1.650970311	2.80748E-07	0.001217492	3.140447848	Acta2	0.873815783	5.56424E-05	0.031448092	1.832503283
Spp1	1.635567432	2.49707E-07	0.001217492	3.107097325	Vav1	0.868863225	0.000640755	0.086373158	1.826223357
Trim7	1.60423652	0.000655995	0.08673138	3.040348116	Selplg	0.867304285	0.0004702	0.075474474	1.824251051
Tmem171	1.564816949	0.000345597	0.064713179	2.95839963	Cyba	0.863918602	0.000109374	0.037812439	1.819974961
F7	1.564298468	2.01265E-07	0.001217492	2.957336619	Ctla2a	0.861228411	7.33339E-05	0.034969723	1.816584418
Stap1	1.542367008	4.88393E-05	0.031448092	2.912719972	Cytip	0.856972995	0.000557988	0.081734682	1.811234062
Apoc2	1.525318985	5.12282E-06	0.01110782	2.878503536	Hcls1	0.851590361	2.45028E-05	0.023099773	1.804489016
Clca3a2	1.474242485	0.000340821	0.064713179	2.778377215	Map3k7cl	0.851255083	3.39334E-05	0.02943113	1.804069706
Crlf1	1.451698573	0.00014029	0.041861881	2.735299054	Adgre1	0.836872036	0.000489703	0.075474474	1.786173268
Kif26b	1.4041084	5.45458E-05	0.031448092	2.646541726	Cd52	0.832569072	0.000103412	0.037812439	1.780853787
Ckap2l	1.330827152	1.4315E-06	0.003448794	2.515468549	Aldh3b1	0.826351599	3.70447E-05	0.03089389	1.773195491
Cd300lf	1.329974215	3.91231E-05	0.031418757	2.513981817	Cthrc1	0.814819765	0.000295344	0.060414591	1.759078378
Gdf15	1.313397121	9.01627E-05	0.037596122	2.48526057	Cd14	0.81470148	0.000282809	0.05953539	1.758934159
Spdl1	1.299908971	0.000591835	0.084985095	2.46213347	Pltp	0.812721738	0.000467082	0.075474474	1.756522111
Cx3cr1	1.290257093	0.000286502	0.059732817	2.445716351	Irf5	0.812022348	0.000453884	0.075126489	1.755670791
Bcl2a1b	1.279942942	6.24598E-06	0.011285962	2.428293728	Pirb	0.810185353	0.000166803	0.045782214	1.753436705
Plxdc1	1.279689019	0.000777849	0.098632164	2.427866373	Ftl1	0.799837161	0.00064874	0.086373158	1.740904617
Sirpb1c	1.264338944	0.000644354	0.086373158	2.40217115	Was	0.797860502	0.000102168	0.037812439	1.73852101
Il1rn	1.238865596	0.000239893	0.056932649	2.360128802	Sinhcaf	0.788145015	7.84813E-05	0.035529994	1.726852686
Sox4	1.231543589	6.30181E-09	6.83211E-05	2.348180955	Anln	0.787410972	0.000175492	0.04697762	1.725974287
Aurkb	1.212065689	0.000190883	0.048693251	2.316691093	Cyth4	0.786039576	0.000788604	0.099414475	1.724334392
Clec4d	1.196445855	0.000143045	0.041914109	2.291743935	Gngt2	0.782041279	0.000109864	0.037812439	1.719562173
Cd72	1.186526759	8.22929E-05	0.03641544	2.276041336	Igsf6	0.773974673	0.00031851	0.062784037	1.709974336
Nfasc	1.185230732	0.000569819	0.082369189	2.2739976	Itgam	0.767836515	8.22905E-06	0.013725424	1.702714454
Lilr4b	1.184608253	0.000690258	0.090161822	2.273016652	Mmp14	0.762944296	7.32983E-05	0.034969723	1.696950281
Glipr1	1.173458573	5.80143E-05	0.031448092	2.255517646	Ms4a6d	0.750958302	0.000275563	0.05915873	1.682910323
Gm49339	1.170611589	5.64402E-05	0.031448092	2.251071044	Snx20	0.741459565	0.000488941	0.075474474	1.6718664
Frzb	1.170374212	3.40482E-07	0.001230445	2.250700689	Tuba1c	0.730830393	1.5734E-05	0.018073585	1.659594054
Ctss	1.16864278	0.000349188	0.064713179	2.248001159	Gpsm3	0.7059351	0.00014839	0.042900641	1.631201605
Pstpip1	1.158761745	1.16587E-05	0.018056756	2.23265718	Ifi30	0.704391959	0.00025459	0.056932649	1.629457766
Ccn2	1.134168525	7.41875E-05	0.034969723	2.194920255	Fcgr3	0.702591548	0.000308103	0.061403285	1.627425552
Lgals3	1.115035532	0.000640801	0.086373158	2.166003437	Litaf	0.696689496	0.00019641	0.048951201	1.62078137
Cdk1	1.113832564	0.000330876	0.0641332	2.164198102	Hmga1	0.6795614	6.43406E-05	0.034026738	1.601652756
Neurl3	1.109323573	2.38947E-05	0.023099773	2.157444688	Plekho1	0.666310048	0.000675041	0.088708538	1.587008712
Ms4a7	1.106807671	0.000122919	0.038075161	2.153685621	Arrb2	0.650727569	0.000397656	0.070100639	1.569959746
Cd300c2	1.102187528	8.58767E-05	0.036511061	2.146799606	Aprt	0.648037635	0.000121509	0.038075161	1.567035253
Ubash3b	1.094640255	0.000270777	0.058712485	2.135598225	Tpd52	0.635919696	5.70199E-05	0.031448092	1.553928039
Atp1a3	1.091523161	0.000649302	0.086373158	2.130989022	Ikbke	0.631447429	0.000528926	0.079643754	1.549118415
Parvg	1.090906185	0.000199583	0.049176827	2.130077889	Iqgap2	0.614766398	0.000379189	0.067950116	1.531310026
Wfdc17	1.090386122	0.000101446	0.037812439	2.129310175	Limd2	0.607409968	0.00045242	0.075126489	1.523521614
Tnc	1.085979119	0.000608246	0.086373158	2.122815693	Dnase2a	0.604207268	0.000187276	0.048436456	1.520143235
Top2a	1.085710409	0.000333122	0.0641332	2.122420344	Efhd2	0.600905852	0.000361545	0.066435362	1.516668567
AB124611	1.070913958	1.26554E-05	0.018073585	2.100763796	Fam49b	0.599712999	0.000645745	0.086373158	1.515415069
Lyz2	1.070197414	0.000252575	0.056932649	2.099720668	Hlf	-0.618969271	5.40157E-05	0.031448092	0.651135963
Atp8b4	1.065670791	5.11918E-05	0.031448092	2.093142874	Sema3d	-0.636338673	4.73006E-05	0.031448092	0.643343581
Dpep2	1.061560999	1.04758E-06	0.002839344	2.087188643	Negr1	-0.702208873	0.000257833	0.056932649	0.614630441
Orai2	1.051866225	0.000203075	0.049475032	2.073209953	Pex11a	-0.810678946	0.000490795	0.075474474	0.570113494
Actg2	1.051164949	5.80384E-06	0.011285962	2.072202436	Tppp	-0.860030965	1.47568E-05	0.018073585	0.550940733
Csf2ra	1.043455029	0.000633565	0.086373158	2.061157898	Slc4a4	-0.874341114	0.000259942	0.056932649	0.545502943
Basp1	1.036689435	0.000474994	0.075474474	2.05151462	Abhd6	-0.885157676	0.000120073	0.038075161	0.541428345
Ugt1a7c	1.028626129	0.00025757	0.056932649	2.040080567	Ebf1	-0.95553329	9.75937E-05	0.037812439	0.515650945
Lrmp	1.019211241	1.58372E-05	0.018073585	2.026810546	Hif3a	-1.595860056	0.0002984	0.060469321	0.330824949
Serpina3g	1.01834632	5.76704E-05	0.031448092	2.025595803	Gm10037	-1.94192995	0.000105042	0.037812439	0.260268036
Ltbp2	1.01670044	9.43354E-05	0.037812439	2.023286245	Fabp3	-2.137495076	0.000620785	0.086373158	0.227274058
F10	1.015288451	8.53201E-05	0.036511061	2.021306991	Phospho1	-2.241313966	0.000258791	0.056932649	0.211493618
Arl11	1.014329815	0.00056166	0.081734682	2.019964327	Kcnj11	-2.794348201	0.00010779	0.037812439	0.144150905
Tyrobp	0.992101862	0.000119198	0.038075161	1.989080772	Sgk2	-3.194678311	0.000489733	0.075474474	0.109220963

Ddah1	0.991874612	0.000347714	0.064713179	1.988767481	Bmp8b	-4.242227155	0.000104865	0.037812439	0.052839948
Lgmn	0.982223052	0.000334227	0.0641332	1.975507128	Spopfm2	-4.327514979	0.000442827	0.074565456	0.049806748
Fcgr1	0.977848156	0.000558785	0.081734682	1.969525581	Cntn1	-4.632324321	0.000249897	0.056932649	0.040321013
Cyp4f18	0.969206874	0.00064903	0.086373158	1.957764012	Pnma2	-5.461485655	0.000469048	0.075474474	0.022694938
Tesk2	0.968910585	0.000290722	0.060035394	1.957361985	Ppp2r2c	-5.513345586	0.000635161	0.086373158	0.021893621
Rac2	0.962215876	0.000443617	0.074565456	1.948300047	Trim63	-6.884744609	0.000406144	0.071019571	0.00846224
Dok3	0.953031235	0.000370054	0.066865611	1.935935967	Mypn	-7.085265575	7.28396E-05	0.034969723	0.007364148

Supplemental Table 3. PANDORA-seq detects significantly changed sperm rsRNA and tsRNA induced by HCD feeding

**Sequence**	Log2(FC)	P Value Adjusted	Annotation
TACGGCAGCGCCGAAGGAGCCTCGGTTGGCCCCGG	7.737179	0.000608	28S-rRNA
GTGGAGCACGAGCGTACGCGTTAGGACCCGAAAGATGGTGAACT	7.363636	0.002284	28S-rRNA
CGAGCGGTCGGCGTCCCCCAACTTCTTAGAGGGACA	7.314633	0.008506	18S-rRNA
TACGCACGGCCGGTACA	7.210977	0.007452	18S-rRNA
GGGACAAGTGGCGTTCAGCCACCCGAGATTGAGCA	7.158858	0.04325	18S-rRNA
ACGAAAGTCGGAGGTTCGAAGACGATCAGA	7.152217	0.016147	18S-rRNA
TCCCGTGGGGAGCTTTGAGAGGCCTGGC	7.151624	0.011528	45S-rRNA
TTGGAATGAGTCCACTTTAAATCCTTTAACGAGGATC	7.091064	0.019866	18S-rRNA
TGGCGTTCAGCCACCCGAGATTGAGCAATAA	7.034794	0.006009	18S-rRNA
TGTGAAGCAGAATTCACCAAGCGTTGG	7.005126	0.025807	28S-rRNA
GAACGCACGTTCGTGTGGAACCTGGCGCTAGACCATTC	6.990223	0.029182	28S-rRNA
AATGTGATTTCTGCCCAGTGCTCTGAATGT	6.982574	0.020823	28S-rRNA
TCCTTCTGATCGAGGCCCAGCCCGTGGACGGT	6.973696	0.004625	28S-rRNA
TTGTCTGTGTGTGTTTGGGTC	6.968994	0.013491	45S-rRNA
GCAGAAGGGCAAAAGCTCGCTTGATCTTGATTTT	6.933828	0.016587	28S-rRNA
TTAATCAAGAACGAAAGTCGGAGGTTCGAAGAC	6.928401	0.019785	18S-rRNA
GGCGAAGCCAGAGGAAACTCTGGTGGAGGTCCGTAGCGGTCCTGA	6.925182	0.033996	28S-rRNA
GAGGGACAAGTGGCGTTCAGCCACCCGAGATTGAGCAA	6.880727	0.016251	18S-rRNA
GACGGCCGGGGGCATTCGTATTGCGCCGCTAGAGGTGAAATTCTT	6.862389	0.014962	18S-rRNA
AAGCTCGCTTGATCTTGATTTTCAGTACGAATA	6.77083	0.025265	28S-rRNA
TGCACGCGCGCTACACTGACTGGCTCAGC	6.757318	0.012363	18S-rRNA
GAGAACTTTGAAGGCCGAA	6.754801	0.034431	28S-rRNA
TCCCTCCCGGCTCCGGCCGGGGGTCGGGCGCCGGCGGCT	6.726978	0.007723	18S-rRNA
AATCAGTTATGGTTCCTTTGGTCGCTCGCT	6.723958	0.026952	18S-rRNA
AGGGCAAAAGCTCGCTTGATCTTGATTTTCA	6.711767	0.033052	28S-rRNA
TGGTAATTCTAGAGCTAATACATGCCGACGGGCG	6.687416	0.010053	18S-rRNA
AGGCGGAACGATACGGCAGCGCCGAAGGAGCCTCGGTTGGCCCCG	6.634378	0.042352	28S-rRNA
CCCGCGGGGCCCGAAGCGTTTACTTTGAAAAAATTAGA	6.61478	0.037421	18S-rRNA
GAACTGGTGCGGACCAGGGGAATCCGACTGTTTAAT	6.579478	0.02754	28S-rRNA
GATGTCCGGGGCTGCACGCGCGCTACACTGACTGGTT	6.566395	0.026789	18S-rRNA
ATATCTTATTGACCCAGATATATTTTGATCAACGGACCAA	6.550755	0.034716	16S-rRNA
GACCTCGATGTTGGATCAGGACATCCCAATGGTGTAGAAGCTAT	6.547439	0.042921	16S-rRNA
TTTTATCCGGTAAAGCGAATGATTAGAGGT	6.537559	0.048871	28S-rRNA
TGCCGACTGGCGATGCGGCGGCGTTATTCCC	6.531319	0.030564	18S-rRNA
TTGGAGGGCAAGTCTGGTGC	6.525904	0.046509	18S-rRNA
CCGCAGCTAGGAATAATGGAATAGGACCGCGGTTCT	6.479516	0.018173	18S-rRNA
TCTTAGAGGGACAAGTGGCGTTCAGCCACCCGAGATTGAGCAAT	6.472874	0.048816	18S-rRNA
TCTGCTCTGGTCGAGGTTGGCGGTTGAGGGTG	6.437727	0.019983	45S-rRNA
TGCAAAGCTGAAACTTAAAGGAATTGACGGAAGGGCAC	6.380262	0.027023	18S-rRNA
AGCTCCCTCCCGGCTCCGGCCGGGGGTCGGGCGCCGGCGGCT	6.364207	0.04556	18S-rRNA
TCGTCGCGCCGTCGGGTCCCGGGGGGACCGTCG	6.283278	0.037741	28S-rRNA
TGCGGCCCCGGGTTCCTCCCGGGGCTACGCCTGTCTGAGCGTCG	6.23186	0.005663	5.8S-rRNA
TAGACCATTCGTAGACGACCTGCTTCTGGGTCGG	6.143321	0.038302	28S-rRNA
GCGGAACGATACGGCAGCGCCGAAGGAGCCTCGGTTGGCCCCGG	6.086812	4.92007635833771e-05	28S-rRNA
TGGCGCTGGAGCGTCGGGCCCATACCCGGCCGTCGCCGG	6.082484	0.042505	28S-rRNA
TCTGATCGAGGCCCAGCCCGTGGACGGTGTGAGGC	6.024933	0.038877	28S-rRNA
TGGATGGCGCTGGAGCGTCGGGCC	6.007826	0.041928	28S-rRNA
TTTGGTGTATGTGCTTGGCTGAGGAGCCAATGGGGCGAAG	5.976743	0.03059	28S-rRNA
ATCTGCTCTGGTCGAGGTTGGCGGTTGAGGGTG	5.956908	0.030692	45S-rRNA
TAAGTCCCTGCCCTTTG	5.829283	0.029645	18S-rRNA
CCGGGCCGTACCCATATCCGCAGCAGGTCTCCAAGGTG	5.69654	0.031139	28S-rRNA
TAATCAAGAACGAAAGTCGGAGGTTCGAAGACGA	5.693601	0.033409	18S-rRNA
GTGGAGCCGGGCGTGGAATGCGAGTGCCTAGTGGGCCACTTTT	5.604064	0.000523	28S-rRNA
TATTAAAGTTGCTGCAGTTAAAAAGCTCGTAGTTGGAT	5.55805	0.026316	18S-rRNA
AGCGTACGCGTTAGGACCCGAAAGATG	5.544108	0.041129	28S-rRNA
AGATACCGTCGTAGTTCCGACCATAAACGATGCCGACTGGCG	5.502296	0.022095	18S-rRNA
TTAAGCCATGCATGTCTAAGTACGCACGGCCGGTACAGTGAAA	5.49154	0.02575	18S-rRNA
TTGCTGCAGTTAAAAAGCTCGTAGTTGGAT	5.441047	0.019505	18S-rRNA
GGGCCGCCCTCTCGCCCGTCACGTTGAACG	5.434551	0.002837	28S-rRNA
TAGATGTCCGGGGCTGCACGCGCGCTACACTGACTGGC	5.406279	0.033996	18S-rRNA
TTTGGTGTATGTGCTTGGCTGA	5.398482	0.035526	28S-rRNA
TCGAACCATCTAGTAGCTGGTTCCCTCCGAAGTTTCCCTCAGG	5.388576	0.010485	28S-rRNA
AGAACGAAAGTCGGAGGTTCGAAGACGATCAGATACCG	5.336231	0.012919	18S-rRNA
TTTGGTGTATGTGCTTGGCT	5.295712	0.011126	28S-rRNA
AAATGGGTAAGAAGCCCGGCTCGCTGG	5.254555	0.022522	28S-rRNA
TGGTGGAGCGATTTGTCTGGTTAATTCCG	5.237982	0.023898	18S-rRNA
GCTCTGGTCGAGGTTGGCGGTTGAGGGT	5.226543	0.013604	45S-rRNA
TGGCCGTTCTTAGTTGGTGGAGCGATTTGTCTGGTTAATTCC	5.216827	0.035737	18S-rRNA
TTGACCCAACACGGGAAACCTCACCCGGCCCGGACACGGACAGG	5.194224	0.012919	18S-rRNA
TAAATGGGTAAGAAGCCCGGCTCGCTGGC	5.163271	0.042842	28S-rRNA
GCCGCGGGTGCGCGTCTCTCGGGGTCGGGGGTGC	5.101372	0.045534	28S-rRNA
TGTCCCGCGCGTGTGGGGGGAAC	5.087428	0.003896	28S-rRNA
TGCTCTCCCGGCACCCATCCCCGCCGCGGCTCTGGCTTTTCTAC	4.968741	0.029286	45S-rRNA
GGGGGCCCAAGTCCTTCTGATCGAGGCCCAGCCCGTGGACGGT	4.95743	0.013423	28S-rRNA
TTGATCTCGCTCTCGGGGACGGGACCGTTCT	4.928574	0.034122	45S-rRNA
TCGGTCGTCCGACCTGGGTATAGGGGCGAAAGACTA	4.922921	0.025217	28S-rRNA
AATCGAACCATCTAGTAGCTGGTTCCCTCCGAAGTTTCCCTCAGG	4.913629	0.04767	28S-rRNA
TATGTGCTTGGCTGAGGAGCCAATGGGGCGAAG	4.852284	0.00724	28S-rRNA

GCAGGAGGTGTCAGAAAAGTTACCACAGGGATAA	4.83689	0.038732	28S-rRNA
TGAGCAATAACAGGTCTGTGATGCCCTTAGATGTCCGGGG	4.832397	0.001065	18S-rRNA
TTCTGATCGAGGCCCAGCCCGTGGACGGTGTGAGGC	4.810624	0.004404	28S-rRNA
TATATTAAAGTTGCTGCAGTTAAAAAGCTCGTAGTTGGAT	4.74591	0.028853	18S-rRNA
TGAGTGTCCCGCGGGGCCCGAAGCGTTTACTTTGAAAAAATTAG	4.744737	0.003453	18S-rRNA
TTGACTCAACACGGGAAACCTCACCCGGCCCGGACACGGACAG	4.729382	0.00121	18S-rRNA
TTTGACTCAACACGGGAAACCTCACCCGGCCCGGACACGGACA	4.684663	0.019459	18S-rRNA
TGGGGGGCCCAAGTCCTTCTGATCG	4.681307	0.023898	28S-rRNA
TCGCGGGCCGCCGGTGAAATACCACTACTCTCATCGT	4.653874	0.046182	28S-rRNA
CGCGACCTCAGATCAGACGTGGCGACCCGCTGGATTTAAGCAT	4.649539	0.023879	28S-rRNA
TTGACAGATTGATAGCTCTTTCTCGATTCCGT	4.644106	0.013423	18S-rRNA
TGCCCTTAGATGTCCGGGGCTGCACGCGCGCTACACTGACTGGCT	4.622184	0.00114	18S-rRNA
TTTACCCTACTGATGATGTGTTGTTGCCATGGTAATCCTGCT	4.61841	0.045429	28S-rRNA
GCGCGCTACACTGACTGGCTCAGCGTGTGCCTACCCTACGCCGG	4.559556	0.012919	18S-rRNA
TGTGGTAATTCTAGAGCTAATACATGCCGAC	4.493104	0.005602	18S-rRNA
ATTGTGAAGCAGAATTCACCAAGCGTTGG	4.484694	0.040351	28S-rRNA
CGTCCCGGCTTCCCTGGGGGGGACCCGGCGTCTGTGGGCT	4.479907	0.045389	45S-rRNA
TGAGTGTCCCGCGGGGCCCGAAGCGTTTACTTTGAAAAAATTAGA	4.463454	0.020801	18S-rRNA
TTATGGTTCCTTTGGTCGCTCGCTCCTCTCCTACTTGGATAACT	4.447297	0.048533	18S-rRNA
TCAAGAACGAAAGTCGGAGGTTCGAAGACGATCAGATACCGTCGT	4.430236	0.0398	18S-rRNA
AAGGAGTCTAACGCGTGCGCG	4.429081	0.032156	28S-rRNA
GTTGGATCTTGGGAGCGGGCGGGCGGTCCGCCGCGAGGCGAG	4.404915	0.044627	18S-rRNA
ATGGCCTCCGTTGCCCTCGGCCGATCGAAAGGGAGTCGGGTT	4.398194	0.011497	28S-rRNA
TCTGCTCTGGTCGAGGTTGGCGGTTGAGGGT	4.34297	0.001447	45S-rRNA
TCCCGACCCGGGGAGGTAGTGACGAAAA	4.326298	0.033772	18S-rRNA
GGTAGTCGCCGTGCCTACCATGGTGACCACGGGTGACGGGGA	4.323368	0.041424	18S-rRNA
CGAGCGGTCGGCGTCCCCCAACTTCTTAGAGGGACAA	4.265185	0.024989	18S-rRNA
AAAGCGAATGATTAGAGGTCTTGGGGCCGA	4.257457	0.041013	28S-rRNA
TGGGGGGAACCTCCGCGTCGGTGTTCCCCCGCCGG	4.239694	0.008515	28S-rRNA
TGGCCTCCGTTGCCCTCGGCCGATCGAAAGGGAGTCGGGTT	4.232505	0.001847	28S-rRNA
TTAAATGGGTAAGAAGCCCGGCTCGCTGGC	4.202876	0.034388	28S-rRNA
TGCTCTGGTCGAGGTTGGCGGTTGAGGGTGT	4.193972	0.006316	45S-rRNA
CAGACCGTGAAAGCGGGGCCTCACGATCCTTCTGA	4.193467	0.013867	28S-rRNA
TTGAGCAATAACAGGTCTGTGATGCCCTTAGATGTCCGGGG	4.17802	0.017229	18S-rRNA
AGGGATAACTGGCTTGTGGCGGCCAAGCGTTCATAGCGACGTCGC	4.174676	0.003017	28S-rRNA
TCGTAGACGACCTGCTTCTGGGTCGGGGTTTCGTACGTAGCA	4.170671	0.032156	28S-rRNA
TATGTGCTTGGCTGAGGAGCCAATGGGGCGAAGCTACCATCT	4.154111	0.001443	28S-rRNA
TGGTTTGACTGTCTCGCCGGTGTCGCGCTTCTCT	4.136217	0.040984	45S-rRNA
TCAAGAACGAAAGTCGGAGGTTCGAAGACGATCAGATACCGTC	4.133327	0.001606	18S-rRNA
TGGATGGCGCTGGAGCGTCGGGCCCATACCCGGCCGTCGCCGG	4.113493	0.008842	28S-rRNA
TGGATGGCGCTGGAGCGTCGGGCCCATACCCGGCCGTCG	4.103832	0.000755	28S-rRNA
TCGAACCATCTAGTAGCTGGTTCCCTCCGAAGTTTCCCT	4.0907	0.012245	28S-rRNA
GACCGCGGTTCTATTTTGTTGGTTTTCGGAACTG	4.077837	0.037872	18S-rRNA
CTCCGGGGACAGTGCCAGGTGGGGAGTTTGA	4.022072	0.020104	28S-rRNA
GTCCTTCTGATCGAGGCCCAGCCCGTGGACGGTGTGAGGCCGGT	3.986362	0.047479	28S-rRNA
TTCCTTTGGTCGCTCGCTCCTCTCCTACTTGGATAACT	3.977479	0.008442	18S-rRNA
ACCGCGACCTCAGATCAGACGTGGCGACCCGCTGAATTTAAGCAT	3.962956	0.004767	45S-rRNA
TTCTGATCGAGGCCCAGCCCGTGGACGGTGTGAGGCCG	3.953766	0.012742	28S-rRNA
TGGAGCGATTTGTCTGGTTAATTCCG	3.949428	0.043574	18S-rRNA
GTCCTTCTGATCGAGGCCCAGCCCGTGGACGGTGTGAGGCCGG	3.946304	0.015572	28S-rRNA
TGGATGGCGCTGGAGCGTCGGGCCCATACCCGGCCGT	3.944652	0.001176	28S-rRNA
AACACGGGAAACCTCACCCGGCCCGGACACGGACA	3.919594	0.0336	18S-rRNA
TAATCAAGAACGAAAGTCGGAGGTTCGAAGACGATCAGATACC	3.906731	0.012919	18S-rRNA
TTGAACGCACGTTCGTGTGGAACCTGGCGCTAGACCA	3.895381	0.037406	28S-rRNA
TTGACTCAACACGGGAAACCTCACCCGGCCCGGACACGGACAGG	3.889922	0.035991	18S-rRNA
TGGATCTTGGGGGCGGGCGGGCGGTCCGCCGCGAGGCGA	3.865695	0.019499	18S-rRNA
TTGGTGGAGCGATTT	3.851872	0.048812	18S-rRNA
TTTAAATGGGTAAGAAGCCCGGCTCGCTGGC	3.848112	0.034631	28S-rRNA
GCAGGTCTCCAAGGTGAACAGCCTCTGGCATGTTGGAA	3.811799	0.025265	28S-rRNA
TCTTGGGAGCGGGCGGGCGGTCCGCCGCGAGGCGA	3.805734	0.031646	18S-rRNA
AAATCGGTCGTCCGACCTGGGTATAGGGGCGAAAGACTA	3.784213	0.001817	28S-rRNA
TAGGAATAATGGAATAGGACCGCGGTTCT	3.767646	0.039895	18S-rRNA
TAGCTGGCGCTCTCGCTCCCGACGTACGCAGTTTTATCCG	3.757857	0.002468	28S-rRNA
GTTGGATCTTGGGAGCGGGCGGGCGGTCCGCCGCGAGGCGAGT	3.751806	0.02659	18S-rRNA
TAGTTCCGACCATAAACGATGCCGACTGGCGATGCGGCGGCGT	3.745005	0.04208	18S-rRNA
GAGGGGCTCTCGCTTCTGGCGCCAAGCGTCCGTCCCGCG	3.729077	0.041226	28S-rRNA
TGGGGCGAAGCTACCATCTGTGGGATTATGACTGAACGCCT	3.724819	0.006368	28S-rRNA
TAGCTGGCGCTCTCGCTCCCGACGTACG	3.722533	0.025265	28S-rRNA
TGACTCTAGATAACCTCGGGCCGATCGCACGCC	3.721409	0.005134	18S-rRNA
TCGTCGCGCCGTCGGGTCCCGGGGGGACCGT	3.714565	0.004106	28S-rRNA
TTTGTACACACCGCCCGTCGCTACTACCG	3.712593	0.036736	18S-rRNA
TAGACGACCTGCTTCTGGGTCGGGGTTTCGTACG	3.696022	0.009495	28S-rRNA
TTAATGTGAATTGCAGGACACATTGATCATCGACACTT	3.679714	0.014622	5.8S-rRNA
CTGCTCTGGTCGAGGTTGGCGGTTGAGGGTGTGC	3.672768	0.011426	45S-rRNA
TTGCGTTGATTAAGTCCCTGCCCTTT	3.665351	0.008842	18S-rRNA
GAACGAAAGTCGGAGGTTCGAAGACGATCAGATACCG	3.647831	0.031932	18S-rRNA
ACGCAGGTGTCCTAAGGCGAGCTCAGGGAGGA	3.631979	0.046367	28S-rRNA
TGGCGCGCGGGCGGGGCCGGGGGTGGGGT	3.631449	0.024214	28S-rRNA
TATGGTTCCTTTGGTCGCTCGCTCCTCTCCTACTTGGATAACT	3.630112	0.007607	18S-rRNA
TGGCCGTTCTTAGTTGGTGGAGCGATTTGTCTGGTTAATTCCG	3.60917	0.013423	18S-rRNA
TGCTTGGCTGAGGAGCCAATGGGGCGAAGCTACCATCTGTGGGA	3.60862	0.004803	28S-rRNA
TGCGGCCCCGGGTTCCTCCCGGGGCTACGCCTGTCTGAGCGTCGC	3.605622	0.003615	5.8S-rRNA

TTAGACCGTCGTGAGACAGGTTAGTTTTACCCTACTGATGATGT	3.593303	0.039895	28S-rRNA
CGCGCGCTACACTGACTGGCTCAGCGTGTGCCTACCCTACGCCGG	3.585925	0.022365	18S-rRNA
GTGGAGCCGGGCGTGGAATGCGAGTGCCTAGTGGGCCACTTTTG	3.56926	0.00516	28S-rRNA
TGGTTCCTTTGGTCGCTCGCTCCTCTCCTACTTGGAT	3.561053	0.03632	18S-rRNA
TGAGGAGCCAATGGGGCGAAGCTACCATCTGTGGGATT	3.560899	0.023826	28S-rRNA
TCGGGACCGGGGTCCGGTGCGGAGAGCC	3.556033	0.043529	28S-rRNA
TAACGCAGGTGTCCTAAGGCGAGCTCAGGGAG	3.5459	0.005545	28S-rRNA
TACATGCCGACGGGCGCTGACCCCCCTTCCCGGGGGGGG	3.536489	0.020823	18S-rRNA
TAACAGGTCTGTGATGCCCTTAGATGTCCGGGG	3.524857	0.001817	18S-rRNA
TGGATCTTGGGAGCGGGCGGGCGGTCCGCCGCGAGGCGG	3.52098	0.023879	18S-rRNA
AGGGCAAAAGCTCGCTTGATCTTGATTTT	3.503119	0.022365	28S-rRNA
TTGTGAAGCAGAATTCACCAAGCGTTGG	3.496175	0.005802	28S-rRNA
TGGATGGTTTAGTGAGGCCCTCGGATCGGCCCCGCCGGGGTCGGC	3.490415	0.019407	18S-rRNA
CGCGACCTCAGATCAGACGTGGCGGCCCGCTGAATTTAAGCAT	3.482188	0.023204	28S-rRNA
TGTGCTTGGCTGAGGAGCCAATGGGGCGAAGCTACCATCT	3.481208	0.004292	28S-rRNA
TACACCTGTCAAACGGTAACGCAGGTGTCCTAAGGCGAGCT	3.470919	0.022031	28S-rRNA
GGCGGCGTCCGGTGAGCTCTCGCTGGCCCTTGAAAATCCGGGGG	3.470915	0.017263	28S-rRNA
TCCGGGGCTGCACGCGCGCTACACTGACTGGCTCA	3.453262	0.035737	18S-rRNA
GTGGAGCCGGGCGTGGAATGCGAGTGCCTAGTGGGCCACTTT	3.444203	0.045644	28S-rRNA
TTATGGTTCCTTTGGTCGCTCGCTCCTCTCCTACTTGGATAA	3.442569	0.038431	18S-rRNA
CGTTGAACGCACGTTCGTGTGGAACCTGGCGCTAGAC	3.439959	0.044528	28S-rRNA
AACCTCCCGTGGAGCAGAAGGGCAAAAGCTCGCTTGAT	3.438721	0.009826	28S-rRNA
TACTTGGATGGGAGACCGCCTGGGAATACCGG	3.434591	0.007531	5S-rRNA
CTGAGTGTCCCGCGGGGCCCGAAGCGTTTACTTTGAAAAAAT	3.434533	0.025265	18S-rRNA
TTAGTGACGCGCATGAATGGATGAACGAGATTCCCAC	3.424672	0.02383	28S-rRNA
TGTGCTTGGCTGAGGAGCCAATGGGGCGAAGCTACCATCTGT	3.419245	0.023227	28S-rRNA
TTCCTTTGGTCGCTCGCTCCTCTCCTACTTGGA	3.411226	0.003851	18S-rRNA
TTTCCTTCTCTAGCTATCTTCCGGAAAGGGTGCGGGCTTCT	3.410155	0.036867	45S-rRNA
TTGACAGATTGATAGCTCTTTCTCGATTCCGTGG	3.400569	0.011692	18S-rRNA
CGCGACCTCAGATCAGGCGTGGCGACCCGCTGAATTTAAGCAT	3.38868	0.025663	28S-rRNA
TGGGTAAGAAGCCCGGCTCGCTGGC	3.384478	0.013491	28S-rRNA
TGGGAGACCGCCTGGGAATACCGGGTGCTG	3.382712	0.031437	5S-rRNA
TTACCCTACTGATGATGTGTTGTTGCCATGGTAATCCTGCT	3.375243	0.031534	28S-rRNA
GCTCTGGTCGAGGTTGGCGGTTGAGGGTGTGC	3.362104	0.006736	45S-rRNA
AGCCACCCGAGATTGAGCAATAACAGGTCTGTG	3.361671	0.022166	18S-rRNA
TGGAGCACGAGCGTACGCGTTAGGACCCGAAAGATG	3.360251	0.006415	28S-rRNA
CGGGAAACCTCACCCGGCCCGGACACGGACAGGATTGACAG	3.354298	0.047479	18S-rRNA
GACAGCAGGACGGTGGCCATGGAAGTCG	3.354239	0.043565	28S-rRNA
CGCGACCTCAGATCGGACGTGGCGACCCGCTGAATTTAAGCAT	3.351189	0.019499	28S-rRNA
AACGCAGGTGTCCTAAGGCGAGCTCAGGGAGGA	3.348039	0.006707	28S-rRNA
GCAGAGCAGCTCCCTCGCTGCGATCTATTGAAAGTCAGCCCTCGA	3.334592	0.041506	28S-rRNA
AACGCAGGTGTCCTAAGGCGAGCTCAGGGAG	3.333309	0.043913	28S-rRNA
GAACGAAAGTCGGAGGTTCGAAGACGATCAGATACCGTC	3.330349	0.027254	18S-rRNA
TTTTGTTGGTTTTCGGAACTGAGGCCATGATTAAGAG	3.325901	0.029645	18S-rRNA
CTGGCTCAGCGTGTGCCTACCCTACGCCGG	3.319223	0.021545	18S-rRNA
AGAAACCTCCCGTGGAGCAGAAGGGCAAAAGCTCGCTTGATCTTG	3.312328	0.011528	28S-rRNA
TTGACGGAAGGGCACCACCAGGA	3.299208	0.020774	18S-rRNA
TTGAACCCCATTCGTGATGGGGATCGGGGATTG	3.29542	0.0097	18S-rRNA
ACGGGAAACCTCACCCGGCCCGGACACGGACAGG	3.292554	0.002421	18S-rRNA
ATAGCGACGTCGCTTTTTGATCCTTCG	3.28242	0.01607	28S-rRNA
TGCCCTTAGATGTCCGGGGCTGCACGCGCGCTACACTGACTGG	3.277951	0.005802	18S-rRNA
GTTCCTTTGGTCGCTCGCTCCTCTCCTACTTGGATAACT	3.27493	0.042518	18S-rRNA
TGAGTGTCCCGCGGGGCCCGAAGCGTTTACTTTGAAAAAAT	3.251937	0.042267	18S-rRNA
AATCGGTCGTCCGACCTGGGTATAGGGGCGAAAGAC	3.247797	0.034478	28S-rRNA
ACGGACCAAGGAGTCTAACGCGTGCGCGA	3.246547	0.006209	28S-rRNA
TCGTCGCGCCGTCGGGTCCCGGGGGGA	3.245211	0.032614	28S-rRNA
TTGGCTGAGGAGCCAATGGGGCGAAGCTACCATCTGTGGGA	3.243014	0.049283	28S-rRNA
ACCTCCCGTGGAGCAGAAGGGCAAAAGCTCGCTTGATCTTG	3.232278	0.034517	28S-rRNA
TGCGTTGATTAAGTCCCTGCCCTTTG	3.229621	0.02497	18S-rRNA
TTGAACGCACGTTCGTGTGGAACCTGGCGCTAGACCATTC	3.229544	0.019543	28S-rRNA
CGCAGGTGTCCTAAGGCGAGCTCAGGGAGGA	3.226937	0.031957	28S-rRNA
TGAACGCACGTTCGTGTGGAACCTGGCGCTAGACCATTC	3.22646	0.012919	28S-rRNA
AAGAACGAAAGTCGGAGGTTCGAAGACGATCAGATACCGTC	3.216448	0.010599	18S-rRNA
AGTGACGCGCATGAATGGATGAA	3.206358	0.044397	28S-rRNA
GACCGCGGTTCTATTTTGTTGGTTTTCGGAA	3.203409	0.014428	18S-rRNA
TAACGCAGGTGTCCTAAGGCGAGCTCAGGGAGGA	3.197635	0.006147	28S-rRNA
TGGATCTTGGGAGCGGGCGGGCGGTCCGCCGCGAGGCGA	3.194025	0.011157	18S-rRNA
TGGCCGTTCTTAGTTGGTGGAGCGATTTGTCTGGTTAATTCCGA	3.191886	0.040763	18S-rRNA
CCCTTAGATGTCCGGGGCTGCACGCGCGCTACACTGACTGGCT	3.160547	0.019983	18S-rRNA
GTCCTTCTGATCGAGGCCCAGCCCGTGGACGGTGTGAGGCCG	3.159774	0.023246	28S-rRNA
AAACTTTAAATGGGTAAGAAGCCCGGCTCGCTGGC	3.153711	0.022713	28S-rRNA
TGGTCGCTCGCTCCTCTCCTACTTGGATAA	3.153181	0.047005	18S-rRNA
TCTAAGTACGCACGGCCGGTACAGTGAAACTGCGAATGGCT	3.147269	0.034431	18S-rRNA
AGTTGGATCTTGGGAGCGGGCGGGCGGTCCGCCGCGAGGCGA	3.141113	0.020823	18S-rRNA
TGCCCTTTGTACACACCGCCCGTCGCTACTACCG	3.130652	0.020774	18S-rRNA
TGTGCTTGGCTGAGGAGCCAATGGGGCGAAGCTACCATCTGTGGG	3.120414	0.006209	28S-rRNA
TTGGATCTTGGGAGCGGGCGGGCGGTCCGCCGCGAGGCGAGT	3.117444	0.014652	18S-rRNA
AAGGAGTCTAACGCGTGCGCGA	3.116505	0.005642	28S-rRNA
CGCGACCTCAGATCAGACGTGGCGACCCGCTGAATTTAAGCATA	3.094274	0.018596	28S-rRNA
TCCTTCTGATCGAGGCCCAGCCCGTGGACGGTGTGAGGCCGG	3.091946	0.010954	28S-rRNA
TTAGACCGTCGTGAGACAGGTTAGTTTTACCCTACTGA	3.081182	0.020723	28S-rRNA
TGCGTCCCGGCTTCCCTGGGGGGGACCCGGCGTCTGTGGGC	3.077536	0.023322	45S-rRNA

TTTGTACACACCGCCCGTCGCTACTACCGA	3.06747	0.019983	18S-rRNA
TCGTCGCGCCGTCGGGTCCCGGGGGGAC	3.062191	0.022131	28S-rRNA
TGATTAAGTCCCTGCCCTTT	3.055889	0.010954	18S-rRNA
TGGCTCTAAGGGCTGGGTCGGTCGGGCTGGGGCGCGAAGCGG	3.048375	0.036434	28S-rRNA
TGGCTCTAAGGGCTGGGTCGGTCGGGCTGGGGCGCGAAGCGGG	3.04488	0.024816	28S-rRNA
TGCCAGGTGGGGAGTTTGA	3.038915	0.028758	28S-rRNA
TGCGTCGATGAAGAACGCAGCTAGCTGCGA	3.037224	0.044528	5.8S-rRNA
AAACGGTAACGCAGGTGTCCTAAGGCGAGCTCAGGGAGGAC	3.026075	0.035457	28S-rRNA
TTTGACTCAACACGGGAAACCTCACCCGGCCCGGACACGGACAG	3.017111	0.02568	18S-rRNA
TATGGTTCCTTTGGTCGCTCGCTCCTCTCCTACTTGGATAA	3.014088	0.028571	18S-rRNA
AAGAACGAAAGTCGGAGGTTCGAAGACGATCAGATACCGTCGTA	3.013195	0.028346	18S-rRNA
TAGACAGCAGGACGGTGGCCATGGAAGTCGGAATCCG	3.006218	0.026607	28S-rRNA
TGTGGGGGGACCCTCCGCGTCGGT	2.990172	0.026352	28S-rRNA
TTTTCGGAACTGAGGCCATGATTAAGAGGGACGGCCGGGG	2.976628	0.008583	18S-rRNA
TGCCGACTGGCGATGCGGCGGCGTTATTCCCATGACCCGCCGGG	2.976446	0.033281	18S-rRNA
CGCGACCTCAGATCAGACGTGGCGACCCGCTGAATTTAAGCAT	2.965561	0.023135	28S-rRNA
TTCCTTTGGTCGCTCGCTCCTCTCCTACTTGGATAA	2.965547	0.046509	18S-rRNA
TAAAGCGAATGATTAGAGGTCTTGGGGCCGAAA	2.964634	0.034431	28S-rRNA
TAGCGACGTCGCTTTTTGATCCTTCG	2.957591	0.039647	28S-rRNA
TGTGCTTGGCTGAGGAGCCAATGGGGCGAAGCTACCAT	2.956547	0.010954	28S-rRNA
TGGTTCCTTTGGTCGCTCGCTCCTCTCCTACTTGGATAA	2.950937	0.007924	18S-rRNA
TCCCTCCCGGCTCCGGCCGGGGGTCGGGCGCCGGCGGCTT	2.94695	0.039076	18S-rRNA
TGCGTTGATTAAGTCCCTGCCCTTT	2.924326	0.03507	18S-rRNA
CACGGGAAACCTCACCCGGCCCGGACACGGACAGG	2.923954	0.042791	18S-rRNA
TTGGTCGCTCGCTCCTCTCCTACTTGG	2.922094	0.023227	18S-rRNA
GTGCGGGCGGGCGCGACCCGCTCCGGGGACAGTGCCAG	2.915512	0.022095	28S-rRNA
GGTAACGCAGGTGTCCTAAGGCGAGCTCAGGGAG	2.91395	0.031015	28S-rRNA
GACAGCAGGACGGTGGCCATGGAAGTCGG	2.89497	0.041472	28S-rRNA
GGGGGGCCCAAGTCCTTCTGATCGAGGC	2.894026	0.042055	28S-rRNA
TTAGAGGGACAAGTGGCGTTCAGCCACCCGAGATTGAGCA	2.891846	0.017804	18S-rRNA
TCAAGAACGAAAGTCGGAGGTTCGAAGACGATCAG	2.871888	0.01079	18S-rRNA
TTCGAACGTCTGCCCTATCAACTTT	2.861753	0.038404	18S-rRNA
TTAAAAAGCTCGTAGTTGGATCTTGGGAGCGGGCGGGCGG	2.853122	0.013447	18S-rRNA
TCTGCTCTGGTCGAGGTTGGCGGTTGAGGGTGTGC	2.852981	0.034101	45S-rRNA
TTGCGTCCCGGCTTCCCTGGGGGGGACCCGGCGTCTGTGGGC	2.844319	0.04233	45S-rRNA
TCTCGGAAGCTAAGCAGGGTCGGGCCTGGTTA	2.827632	0.044397	5S-rRNA
TTTGACTCAACACGGGAAACCTCACCCGGCCCGGACACGGACAGG	2.815921	0.045711	18S-rRNA
GGGACGGCCGGGGGCATTCGTATTGCGCCGCTAGAGGTGAA	2.804449	0.035481	18S-rRNA
AACACGGGAAACCTCACCCGGCCCGGACACGGACAGG	2.802967	0.028131	18S-rRNA
CACGGACCAAGGAGTCTAACGCGTGCGCGAGT	2.801661	0.02497	28S-rRNA
AACAGGTCTGTGATGCCCTTAGATGTCCGGGGCTGCACGCGCG	2.795695	0.047788	18S-rRNA
TGATCCTTCGATGTCGGCTCTTCCT	2.785799	0.04276	28S-rRNA
AAGCGCGGGTAAACGGCGGGAGTAACTATGACTCTCT	2.779076	0.044627	28S-rRNA
AGGATAGCTGGCGCTCTCGCTCCCGACGTACGCA	2.776492	0.032956	28S-rRNA
AACACGGGAAACCTCACCCGGCCCGGACACGGACAG	2.766721	0.042267	18S-rRNA
GTTGGATCTTGGGAGCGGGCGGGCGGTCCGCCGCGAGGCGA	2.760969	0.026346	18S-rRNA
TGCCGACGGGCGCTGACCCCCCTTCCCGGGGGGGG	2.749215	0.041506	18S-rRNA
TACCGCAGCTAGGAATAATGGAATA	2.738258	0.024401	18S-rRNA
TTGATCCTTCGATGTCGGCTCTTCCTATC	2.724361	0.036564	28S-rRNA
TAAAAAGCTCGTAGTTGGATCTTGGGAGCGGGCGGGCGG	2.721804	0.044262	18S-rRNA
TCCTTCTGATCGAGGCCCAGCCCGTGGACGGTGTGAGGCCG	2.712123	0.016458	28S-rRNA
ACGGGAAACCTCACCCGGCCCGGACACGGACAGGATTGACAG	2.710031	0.041506	18S-rRNA
TTGAACGCACGTTCGTGTGGAACCTGGCGCTAAACCATTC	2.700737	0.025265	28S-rRNA
TGGGAGACCGCCTGGGAATCCCGGGTGCTGTAGGCT	2.697036	0.045833	5S-rRNA
TGGAGCCGGGCGTGGAATGCGAGTGCCTAGT	2.694931	0.008047	28S-rRNA
TTGATTAAGTCCCTGCCCTTT	2.693862	0.046509	18S-rRNA
GGCGGGTGTTGACGCGATGTGATTTCTGCCCAGTGCTCTGA	2.674957	0.009666	28S-rRNA
TGCTCTGGTCGAGGTTGGCGGTTGAGGGTGTGC	2.669834	0.038127	45S-rRNA
TAGTTGGATCTTGGGAGCGGGCGGGCGGTCCGCCGCGAGGCGAG	2.666877	0.025704	18S-rRNA
TGAAGGTGAAGGGCCCCGCCCGGGGGCCCGAGGTGGG	2.666849	0.035625	28S-rRNA
ATCAAGAACGAAAGTCGGAGGTTCGAAGACGATCAGA	2.663848	0.029157	18S-rRNA
AGATGTCCGGGGCTGCACGCGCGCTACACTG	2.661848	0.040145	18S-rRNA
TACCACCCTGAACGCGCCCGATCTCGTCTGATCTCGGAAGCTG	2.64116	0.04767	5S-rRNA
TGGTTCCTTTGGTCGCTCGCTCCTCTCCTACTTGGATAACTG	2.609833	0.023529	18S-rRNA
TGAGGCGGGGGGGCGAGCCCCGAGGGGCTCT	2.604141	0.048346	28S-rRNA
TCAGTCGGTCCTGAGAGATGGGCGAGTGCCGTTCCGAAGGGA	2.602593	0.044627	28S-rRNA
TTTTCGGAACTGAGGCCATGATTAAGAGGGACGGCCGGG	2.602005	0.040145	18S-rRNA
TGGTTCCTTTGGTCGCTCGCTCCTCTCCTAC	2.59972	0.019064	18S-rRNA
TGATCTCGGAAGCTAAGCAGGGTCGGGCCTGGTTA	2.586608	0.032614	5S-rRNA
TACCGCAGCTAGGAATAATGGAATAGGACCGCGGTTCT	2.585698	0.022376	18S-rRNA
TTGGATCTTGGGAGCGGGCGGGCGGTCCGCCGCGAGGCGA	2.577692	0.04032	18S-rRNA
TTGGTCGCTCGCTCCTCTCCTACTTGGATAA	2.573553	0.042267	18S-rRNA
AACAGGTCTGTGATGCCCTTAGATGTCCGGGG	2.569349	0.031855	18S-rRNA
TGTCCGGGGCTGCACGCGCGCTACACTGACTGGCT	2.553925	0.03031	18S-rRNA
TTTCCCTCAGGATAGCTGGCGCTCTCGCTCCCGACGTA	2.547372	0.0427	28S-rRNA
TTAGACCGTCGTGAGACAGGTTAGTTTTACCCTACTGATGA	2.529703	0.017021	28S-rRNA
TGTGGGGGGAACCTCCGCGTCG	2.515768	0.049079	28S-rRNA
CCGCGACCTCAGATCAGACGTGGCGACCCGCTGAATTTAAGCAT	2.51477	0.046735	45S-rRNA
TCCTTCTGATCGAGGCCCAGCCCGTGGACGGTGTGAGGCCGGT	2.512028	0.025491	28S-rRNA
TAGGAATAATGGAATAGGACCGCGGTTC	2.510914	0.041544	18S-rRNA
TTCCGTGGGTGGTGGTGCATGGCCGTTCTTA	2.502536	0.036669	18S-rRNA
TAGATGTCCGGGGCTGCACGCGCGCTACACTGACTGGCT	2.50225	0.034623	18S-rRNA

ACGGTAACGCAGGTGTCCTAAGGCGAGCTCAGGGAGGA	2.49713	0.040384	28S-rRNA
TAGACGACCTGCTTCTGGGTCGGGGTTTCGTACGTAG	2.479114	0.033409	28S-rRNA
TGGCGACCCGCTGAATTT	2.476336	0.043316	28S-rRNA
TGAAAGCGGGGCCTCACG	2.468253	0.014898	28S-rRNA
TGTCCGGGGCTGCACGCGCGCTACACTGAC	2.455139	0.041236	18S-rRNA
GTCCTTCTGATCGAGGCCCAGCCCGTGGACGGTGTGAGGC	2.454463	0.040379	28S-rRNA
TGGAGCACGAGCGTACGCGTTAGGACCCGAAAGATGGTGAACT	2.446141	0.009557	28S-rRNA
TTCCGGCGGCGTCCGGTGAGCTCTCGCTGGCCC	2.441486	0.013984	28S-rRNA
TTTCCCTCAGGATAGCTGGCGCTCTCGCTCCCGACGTACGCA	2.430087	0.029526	28S-rRNA
TGGAGCCGGGCGTGGAATGCGAGTGCCTAGTGGGCCACTTTT	2.422322	0.031139	28S-rRNA
TACCACCCTGAACGCGCCCGATCTCGTCTGATCTCGGAAGC	2.387189	0.035254	5S-rRNA
TGGAACCTGGCGCTAGACCATTCGTAGACGACCTGC	2.386641	0.039647	28S-rRNA
TTGGTCGCTCGCTCCTCTCCTACTTGGATAACTGTGGTAA	2.364803	0.033605	18S-rRNA
AAATCTCGCGCCGGGCCGTACCCATATCCGCAGC	2.361967	0.04096	28S-rRNA
TGTCCGGGGCTGCACGCGCGCTACACTGACTGGCTC	2.358214	0.046182	18S-rRNA
AAACGGTAACGCAGGTGTCCTAAGGCGAGCTCAGGGAG	2.328068	0.048374	28S-rRNA
TATGGTTCCTTTGGTCGCTCGCTCCTCTCCTAC	2.316014	0.032068	18S-rRNA
AGTTGAACATGGGTCAGTCGGTCCTGAGAGATGGGCGA	2.310388	0.026316	28S-rRNA
TGGAGCCGGGCGTGGAATGCGAGTGCCTAGTGGGCCACTTT	2.307224	0.026316	28S-rRNA
CTCTGGTCGAGGTTGGCGGTTGAGGGTGTGC	2.249149	0.031534	45S-rRNA
TCTGGCACGGTGAAGAGACATGAGAGG	2.235861	0.02568	28S-rRNA
TTGGTCGCTCGCTCCTCTCCTA	2.227199	0.033996	18S-rRNA
TTGTCTGGTTAATTCCGATAACGAACGAGACTCTGG	2.221548	0.015439	18S-rRNA
TAGTCAGCGGAGGAAAAGAAACTAAC	2.214818	0.024672	28S-rRNA
TTTTCGGAACTGAGGCCATGATTAAGAGGGACGGCC	2.209768	0.041506	18S-rRNA
GGGTCTTCCCGGAGTCGGGTTGCTTGGGA	2.206698	0.046182	28S-rRNA
TTAGTCAGCGGAGGAAAAGAAACTAAC	2.203492	0.04085	28S-rRNA
TGGAGCCGGGCGTGGAATGCGAGTGCCTAG	2.167113	0.024708	28S-rRNA
TCGCGGGCCGCCGGTGAAATACCACTACTCTCATC	2.071401	0.033052	28S-rRNA
TGGAGCCGGGCGTGGAATGCGAGTGCCTAGTGGGCCACTTTTG	2.000634	0.043928	28S-rRNA
TTTGTCTGGTTAATTCCGATAACGAACGAGACTCTGG	1.983647	0.028853	18S-rRNA
GTGGAGCCGGGCGTGGAATGCGAGTGCCTAGT	1.967123	0.020165	28S-rRNA
TGGAGCACGAGCGTACGCGTTAGGACCCGAAAGATGGTGAAC	1.945309	0.047158	28S-rRNA
TCGGGTCTTCCCGGAGTCGGGTTGCTTGGGA	1.944342	0.039801	28S-rRNA
AGTGCCAGGTGGGGAGTTTGA	1.700055	0.044885	28S-rRNA
TGGAGCCGGGCGTGGAATGCGAGTGCCTAGTGGGC	1.458571	0.047343	28S-rRNA
ACGGCCATACCACCCTGAACGCGCCCGATCTCGTCT	-1.97267	0.019499	5S-rRNA
CGGTCGGGCTGGGGCG	-2.00635	0.041236	28S-rRNA
TACGGCCATACCACCCTGAACGCGCCCGATCTCGTCT	-2.01604	0.045187	5S-rRNA
GGGGGCCGGCGGCGGCGGCGACTCTGGACGCGAG	-2.0287	0.037847	28S-rRNA
CGGGGGGCCGGCGGCGGCGGCGACTCTGGACGCGAG	-2.18539	0.032068	28S-rRNA
CGGTCGGGCTGGGGC	-2.2103	0.03632	28S-rRNA
CCTGTCTGAGCGTCGC	-2.21681	0.020116	5.8S-rRNA
GGCGGGTGTTGACGCGATGTGAT	-2.25501	0.025265	28S-rRNA
GGGGGCCGGCGGCGGCGGCGACTCTGGACGCG	-2.33509	0.00249	28S-rRNA
GGGGGGCCGGCGGCGGCGGCGACTCTGGACGCG	-2.41686	0.001614	28S-rRNA
CCGGGTGCTGTAGGCTT	-2.43479	0.011796	5S-rRNA
GGGGGGCCGGCGGCGGCGGCGACTCTGGACGC	-2.46159	0.006218	28S-rRNA
CGGGGGGCCGGCGGCGGCGGCGACTCTGGACGCGAGC	-2.47692	0.036867	28S-rRNA
AGGACGGTGGCCATGGAAGTCGGAATCCGCTAAGGAGTGTGTAA	-2.4786	0.040212	28S-rRNA
AGATCAGACGTGGCGACCCGCTGAATTTAAG	-2.51109	0.03867	28S-rRNA
CGGTCGGGCTGGGGCGC	-2.55948	0.023549	28S-rRNA
CGCGGTTCCGGCGGCGTCCGGTGAGCTCTCGCTGGC	-2.56884	0.04085	28S-rRNA
TGGGGCGCGAAGCGGGG	-2.58459	0.000544	28S-rRNA
TCGCGGCGTGGGAAATGTGGCGTACGGAAGATCCA	-2.58607	0.041933	28S-rRNA
GGGGGCCGGCGGCGGCGGCGACTCTGGACGC	-2.70713	0.000608	28S-rRNA
GCCTGTCTGAGCGTCGC	-2.76048	0.047875	5.8S-rRNA
CCGCCGCGCCGGGGAGGTGGAG	-2.81513	0.032771	28S-rRNA
AGGTGGGGAGTTTGACTGGGGCGGTA	-2.82227	0.044262	28S-rRNA
AGACGTGGCGACCCGCTGAATT	-2.85983	0.018802	28S-rRNA
ACCGCCCGTCCCCGCCCC	-2.88092	0.004946	18S-rRNA
AAGGGCTGGGTCGGTCGGGC	-2.93012	0.034081	28S-rRNA
AGGATAGCTGGCGCTCTCG	-2.93583	0.032162	28S-rRNA
GGTCGCGCCGTCGGG	-2.96741	0.016244	28S-rRNA
GGGGGCCGGCGGCGGCGGCGACTCTGGACGCGA	-2.98133	0.000207	28S-rRNA
AATACCGGGTGCTGTAGGCTT	-2.99412	0.042267	5S-rRNA
ACGCCTGTCTGAGCGTCGC	-2.99503	0.034266	5.8S-rRNA
GGGTGCTGTAGGCTT	-3.02578	0.006717	5S-rRNA
AAGACGGACCAGAGCGAAAGCATTTGCCAAGAATGTTTT	-3.02593	0.005163	18S-rRNA
ACGGCCCTGGCGGAGCGCTGAGAAGA	-3.02882	0.01816	18S-rRNA
ACGGGGTCGGCGGCGAC	-3.03958	0.0237	28S-rRNA
GGGCCTGGTTAGTACTTGGATGGGAGA	-3.0546	0.002364	5S-rRNA
ATTGATCATCGACACTTCGAACGCACTTG	-3.08901	0.027023	5.8S-rRNA
CGGAACGGAACGGGACGGGAGCGGCCGCGGGTGCGCGTCTCT	-3.10875	0.049425	28S-rRNA
AAATCGGTCGTCCGACCTGGG	-3.11786	0.037421	28S-rRNA
AGCGGTGGATCACTCGGCTCG	-3.13284	0.017946	5.8S-rRNA
AGCGGTGGATCACTCGGCTCGTGCGT	-3.13458	0.044433	5.8S-rRNA
GGGGCCGGCGGCGGCGGCGACTCTGGACG	-3.13978	0.04211	28S-rRNA
ACCACCCTGAACGCGCCCGATCTCGTCTGATC	-3.15654	0.008203	5S-rRNA
CGGGGGGCCGGCGGCGGCGGCGACTCTGGACG	-3.16274	0.02631	28S-rRNA
ACTTGGATAACTGTGG	-3.16995	0.033996	18S-rRNA
CGCCTGTCTGAGCGTCGC	-3.22934	0.00359	5.8S-rRNA

AGAACTGGTGCGGAC	-3.24456	0.033772	28S-rRNA
AGGGCGCGGGCCCGGGTGGAG	-3.2765	0.028627	28S-rRNA
CGCGGTTCCGGCGGCGTCCGGTGAGCTCTCGCTGGCCCT	-3.28159	0.041544	28S-rRNA
GGTGGAGGTCCGTAGCGGTCCTGACGTG	-3.28947	0.032863	28S-rRNA
TCGATGTGGTGCTCCGGAG	-3.30052	0.045089	45S-rRNA
CGCCTGGGAATACCGGGTGCTGTAGGCT	-3.33542	0.012959	5S-rRNA
CGCCCGTCCCCGCCC	-3.36839	0.048428	18S-rRNA
CAGGTGGGGAGTTTGACTGGGGCGG	-3.37157	0.020378	28S-rRNA
CGGCGGCGGCGGCGACTC	-3.37586	0.006736	28S-rRNA
ACGTTCGTGGGGAACCTGGCGCTAAACCATTCG	-3.38724	0.01819	28S-rRNA
CTGGGGCGCGAAGCGGGG	-3.39237	2.70312747112557e-06	28S-rRNA
CGCGGTTCCGGCGGCGTCCGGTGAGCTCTCGCTGG	-3.40296	0.02956	28S-rRNA
AGACGTGGCGACCCGCTGAATCTAAG	-3.43097	0.033176	28S-rRNA
ACCCTGAACGCGCCCGATCTCGTCTGATC	-3.43357	0.024708	5S-rRNA
AGAGGAAACTCTGGTGGAGGTCCGTAGCGGTCCTGACGT	-3.43984	0.033526	28S-rRNA
GTGGGGAACCTGGCGC	-3.44379	0.019866	28S-rRNA
AGAGGAAACTCTGGTGGAGGTCCGTAGCGGTCCTGACGTG	-3.44743	0.012722	28S-rRNA
CCTCAGATCAGACGTGGCGACCCG	-3.44822	0.000997	28S-rRNA
AGACGACCTGCCTCTGGGTCGGGGTTTCGTACGTAG	-3.44828	0.048692	28S-rRNA
ACCACCCTGAACGCGCCCGATCTCGTCTGATCT	-3.44944	0.013796	5S-rRNA
AGACGCGGCGACCCGCTGAATT	-3.45337	0.013643	28S-rRNA
ATTCGTGATGGGGATCGGGGATTGCAATTATTCCC	-3.4557	0.02667	18S-rRNA
GGGAACGGGCTTGGCGG	-3.4696	0.048533	28S-rRNA
AGACGTGGCGACCCGCTGAATTTAAGCA	-3.48032	0.002143	28S-rRNA
AAGGATTGGCTCTAAGGGCTGGGT	-3.48508	0.028254	28S-rRNA
GCGGGCCGCCGGTGAAATACCACTACTCTGATCGTTTTTT	-3.49482	0.014456	28S-rRNA
ATACCACCCTGAACGCGCCCGATCTCGTCTGATC	-3.51001	0.028368	5S-rRNA
AGGCGTGGCGACCCGCTGAATT	-3.5144	0.037536	28S-rRNA
AGCGGTGGATCACTCGGCTCGTG	-3.51541	0.027529	5.8S-rRNA
ACGGTGAAGAGACATGAGAGGTGTAGAA	-3.51754	0.023556	28S-rRNA
AGGGTCGGGCCTGGTTAG	-3.54432	0.003525	5S-rRNA
AGACGTAGCGACCCGCTGAATT	-3.57626	0.049439	28S-rRNA
AGGGTCGGGCCTGGT	-3.5889	0.00401	5S-rRNA
ACCCTGAACGCGCCCGATCTCGTCTGATCTCGGAAGCTAAG	-3.60693	0.026458	5S-rRNA
AGATCTCGCGCCGGGCCG	-3.61122	0.014652	28S-rRNA
CTGGGAATACCGGGTGCTGTAGGCTT	-3.68975	0.023322	5S-rRNA
CTCGGGGTCGGGGGTG	-3.69186	0.046509	28S-rRNA
ATCGATGTGGTGCTCCGGA	-3.69338	0.033052	45S-rRNA
GTTGAACCCCATTCGTGATGGGGATCGGGGATTGCAAT	-3.71245	0.044377	18S-rRNA
AGTGGGCCACTTTTGGTAAGCAGAACTGGCGCTGCGGGATGAA	-3.71292	0.041662	28S-rRNA
GGGATCCCGAGGCCTCTC	-3.71394	0.004448	28S-rRNA
ACCGGGTGCTGTAGGCCT	-3.75661	0.047799	5S-rRNA
ATTCGTGATGGGGATCGGGGATTGCAAT	-3.76012	0.025276	18S-rRNA
GGAATACCGGGTGCTGTAGGC	-3.76866	0.022095	5S-rRNA
TACCTGGTTGATCCTGC	-3.7752	0.003753	45S-rRNA
CGGAAGCTAAGCAGGGTCGGGCCTGGT	-3.78048	0.04556	5S-rRNA
GCGTTGAACGCACGTTCGTGTGGAACCTGGCGC	-3.78942	0.049782	28S-rRNA
CGATCTCGTCTGATCTCGGAAGC	-3.79415	0.012919	5S-rRNA
AGACATGGCGACCCGCTGAATT	-3.79852	0.043529	28S-rRNA
AAAGTCTTTGGGTTCCGGGGGGAG	-3.83659	0.010824	18S-rRNA
AGGACGGTGGCCATGGAAGTCGGAATCCGC	-3.83702	0.002088	28S-rRNA
ATGAGAGGTGTAGAA	-3.83762	0.009218	28S-rRNA
TCAGATCAGACGTGGCGACCCG	-3.87505	0.040566	28S-rRNA
GGACGTGGCGACCCGCTGAATT	-3.88309	0.002898	28S-rRNA
CGCCTGGGAATACCGGGTGCTGTAGGCTT	-3.8976	0.013752	5S-rRNA
CGACTCTTAGCGGTGGAT	-3.89986	0.039338	45S-rRNA
GCGGGCGGGGCCGGGG	-3.92593	1.47539531616097e-07	28S-rRNA
GGTGGATCACTCGGCTCGTGCGTCGATGAAGAACG	-3.92891	0.042008	5.8S-rRNA
GCGCGCCGGGCCCGGGTCTTCCC	-3.93313	0.040993	28S-rRNA
ATGGAAGTCGGAATCCGC	-3.96065	0.019459	28S-rRNA
AACGGCGAGTGAACAGGGAAGAG	-3.96561	0.015194	28S-rRNA
AGACGACCCGCTTCTGGGTCGGGGTTTCGTACG	-3.97336	0.044371	28S-rRNA
ACGCCTGTCTGAGCGTCGCT	-3.98812	0.034122	5.8S-rRNA
GGGGCCGGCGGCGGCGGCGACTCTGGACGCG	-3.98918	0.000159	28S-rRNA
CGACTCTTAGCGGTGGATCACT	-4.01032	0.011464	45S-rRNA
AGACGTGGCGACCCGCCGAATT	-4.01124	0.004067	28S-rRNA
CGGGGCCGGGGGTGGG	-4.01888	0.001764	28S-rRNA
ACCCTGAACGCGCCCGATCTCGTCTGATCT	-4.02167	0.009151	5S-rRNA
GATCCTTCGATGTCGGC	-4.02415	0.040351	28S-rRNA
AGACGACCTGCTTCTGGGTCGGGGTTTCGTGCGTAG	-4.03352	0.032167	28S-rRNA
ATCGACACTTCGAACGCACTTG	-4.03873	0.045936	5.8S-rRNA
CGGGCGGGGCCGGGG	-4.03881	8.78199340270015e-05	28S-rRNA
CGGCGCGCCGGGCTCGGGTCTTCC	-4.04387	0.025187	28S-rRNA
CGGAACGGAACGGGACGGGAGCGGCCGCGGGTGCGCGTCTCTCGG	-4.06045	0.004472	28S-rRNA
GGGCGGGGCCGGGGG	-4.06673	0.027924	28S-rRNA
CTCAGATCAGACGTGGCGACCCG	-4.06871	0.000207	28S-rRNA
ACGGCCCTGGCGGAGC	-4.07518	0.001118	18S-rRNA
AGACGACCCGCTTCTGGGTCGGGGTTTCGTACGTAG	-4.08357	0.032956	28S-rRNA
GGGGCCGGCGGCGGCGGCGACTCTGGACGC	-4.09505	1.0467125719249e-06	28S-rRNA
ACGGCGCGACCGCTCTCC	-4.09609	0.004838	28S-rRNA
CGTCTGATCTCGGAAGCTAAGCAGGGTCGGGCCTGGT	-4.09883	0.039709	5S-rRNA
GCTCCGGGACGGCTGGG	-4.1238	0.023826	28S-rRNA

CGGGTTGCTTGGGAATG	-4.13103	0.0071	28S-rRNA
CCGGGGGGCGGGCGCCGGC	-4.13769	0.007075	18S-rRNA
GGCGGGTGTCGACGCGATGTGATTT	-4.13923	0.030243	28S-rRNA
ACCGCCCGTCCCCGCCC	-4.14313	4.06550055702131e-09	18S-rRNA
ACTTTTGGTAAGCAGAACTGGCGCTGCGGGATGAAC	-4.15625	0.013423	28S-rRNA
CACGGCGCGACCGCTCTCC	-4.16023	0.020774	28S-rRNA
AGAGGAAACTCTGGTGGAGGTCCGTAGCGGTCCTGACGTGCAAA	-4.17505	0.025273	28S-rRNA
CGGCGGCGGCGACTCTGGACGCGAGCCGGGCCC	-4.17586	0.013801	28S-rRNA
GGTGAAGAGACATGAGAGGTGTAGAATAAG	-4.1961	0.04144	28S-rRNA
CGACTCTTAGCGGTGGGTCACTCGGCT	-4.21706	0.02098	45S-rRNA
AGACGACCTGCTTCTGGGTCGGGGTTCCGTACGTAG	-4.22635	0.008884	28S-rRNA
ATGAGAGGTGTAGAATAAGTGGGAGGCCCCCGGCGCCCGG	-4.23212	0.044268	28S-rRNA
AGTCAGCGGAGGAGAAGAAAC	-4.23648	0.008244	28S-rRNA
GGGGGCGGGCGCCGGCGG	-4.23962	1.04725860303025e-05	18S-rRNA
CGGAACGGAACGGGACGGGAGCGGCCGCGGGTGCGCG	-4.24457	0.001439	28S-rRNA
AAGTGGCGTTCAGCCACCCGAGATTGAG	-4.25987	0.032956	18S-rRNA
GCGACGAGTAGGAGGGCCGCTGCGGTGAGCCTTGAAGCC	-4.28622	0.008506	28S-rRNA
AGACGTGGCGACCCGCTGGATT	-4.29371	0.002722	28S-rRNA
CGGCTCCGGGACGGCTGGGA	-4.2968	0.001066	28S-rRNA
CGCGACCTCAGATCAGGCGTGGCGACCCGC	-4.30408	0.031456	28S-rRNA
AGGTGGGGAGTTTGAC	-4.32472	0.02707	28S-rRNA
AGATCAGACGTGGCGACCCGCTGAATTTAAGCA	-4.32801	0.025265	28S-rRNA
CGCGCCGGGCCCGGGTCTTCCC	-4.33419	0.002658	28S-rRNA
GGTGGATCACTCGGCTCGTG	-4.34264	0.005209	5.8S-rRNA
AGTGAGGCCCTCGGAT	-4.34413	0.007696	18S-rRNA
ATGGAAGTCGGAATCCGCTAAGGAGTGTGTAACAACTCACC	-4.34452	0.003459	28S-rRNA
AGACGACCTGCTTCTGGGCCGGGGTTTCGTACGTAG	-4.35425	0.008506	28S-rRNA
GGGGCGCGAAGCGGGG	-4.39728	1.47539531616097e-07	28S-rRNA
GGGGCTGGGCGCGCGCCGCGGCTGGA	-4.39967	0.011528	28S-rRNA
GGCGGGCGGGGCCGGGG	-4.40921	9.40437129904846e-05	28S-rRNA
AGACGTGGCGGCCCGCTGAATT	-4.4199	0.004292	28S-rRNA
AGACGACCTGCTTCTGGGTCGGGGCTTCGTACGTAG	-4.43058	0.021626	28S-rRNA
AGGGCGCGGGCCCGGGTGGAGC	-4.43068	0.025295	28S-rRNA
AGGACGGTGGCCATGGAAGTCGGAATCCGCTAAGGAGTGTGTAG	-4.43153	0.020379	28S-rRNA
GCTGCGGGATGAACCGAACGCCGGGTTAAGGCGCC	-4.43504	0.015837	28S-rRNA
AGACGCGGCGACCCGCTGAATTTAAGCA	-4.43806	0.03543	28S-rRNA
AGACGTGGCGACCCGCTGGATTTAAGCA	-4.44849	0.022376	28S-rRNA
CGAAGTGGAGAAGGGTTCCATGTGAACAGCAGTTGAA	-4.45718	0.020774	28S-rRNA
CGGCTCCGGGACGGCTGG	-4.46391	2.07651360745009e-07	28S-rRNA
ACGTTGGACGCACGTTCGTGTGGAACCTGGCGCTAAACCATTCG	-4.4642	0.030422	28S-rRNA
ACTTGGATGGGTGACCGCCTGGGAATACCGGGTGCTGTAGGCTT	-4.48542	0.016123	5S-rRNA
GGCTCCGGGACGGCTGGGA	-4.48827	0.002913	28S-rRNA
ACCGCCCGTCCCCGCC	-4.49602	0.00133	18S-rRNA
AGGCGACCTGCTTCTGGGTCGGGGTTTCGTACG	-4.49861	0.022376	28S-rRNA
AGACGTGGCGACTCGCTGAATT	-4.503	0.031869	28S-rRNA
AGATACCCGTCGCCGCGCTCTCT	-4.50733	0.018258	28S-rRNA
AGACGACCTGCTTCTGGGTCGGGGTTTTGTACGTAG	-4.50946	0.042842	28S-rRNA
AGGTCTCCAAGGTGAACAGCCTCTGGCATGTTGGAACAATGTAGG	-4.50946	0.023062	28S-rRNA
ACTTCGAACGCACTTGCGGCCCCGGGTTCCTCCCGGGGCTACG	-4.55158	0.047479	5.8S-rRNA
ACTTGTATGGGAGACCGCCTGGGAATACCGGGTGCTGTAGGCTT	-4.55635	0.024401	5S-rRNA
ACGTTGAACGCACGTTCGTGTGGAGCCTGGCGCTAAACCATTCG	-4.55672	0.023826	28S-rRNA
GTTGCCCTCGGCCGATCGAAAGGGAGTCGGGTTCAGATCCC	-4.57666	0.042771	28S-rRNA
GGGCGGGCGGTCCGCCGCGAGGCGAG	-4.57807	0.000542	18S-rRNA
ATCGATGTGGTGCTCCGGAGT	-4.59317	0.012282	45S-rRNA
AGACGTGGCGACCTGCTGAATT	-4.59975	0.002763	28S-rRNA
ATTCGTATTGCGCCGCTAGAGGTGAAATTCTTGGAC	-4.60909	0.020774	18S-rRNA
CCCGGGGCCGAGGGAG	-4.61819	3.39445030830454e-07	28S-rRNA
GAAAATGGATGGCGCTGGAGCGTCGGGCC	-4.61856	0.032614	28S-rRNA
CCGGGGGGCGGGCGCCGGCGG	-4.63921	0.002725	18S-rRNA
CGGGGGGCGGGGCGG	-4.641	0.000279	28S-rRNA
GTGCGTCGATGAAGAACGCAGCTAGCTG	-4.6644	0.001383	5.8S-rRNA
AGATACCCGTCGCCGCGCTCTCTCT	-4.69739	0.031576	28S-rRNA
GGCCCTGGCGGAGCGCTGAGAAGACG	-4.69988	0.034266	18S-rRNA
CGGGGGGCGGGGCGGA	-4.70667	0.000214	28S-rRNA
CGCGACCTCGGATCAGACGTGGCGACCCGC	-4.71752	0.022031	28S-rRNA
CGCGACCTCAGATCAGACGCGGCGACCCGC	-4.7192	0.013727	28S-rRNA
CGACCCTTAGCGGTGGATCACTCGGCT	-4.72509	0.043881	45S-rRNA
TGATCCTTCGATGTCGGC	-4.72565	0.039013	28S-rRNA
AACGGCGAGTGAACAGGGAAGAGCCCAG	-4.7269	0.005642	28S-rRNA
AGTGCGGTAACGCGACCGATCCCGGAGAAGC	-4.744	0.014631	28S-rRNA
GTTGAACGCACGTTCGTGTGGAACCTGGC	-4.74536	0.033467	28S-rRNA
AGACGTGGCGACCCGCTGAATC	-4.75324	0.008244	28S-rRNA
CGGTGGATCACTCGG	-4.7609	0.004167	5.8S-rRNA
ATCGATGTGGTGCTCCGGAGTTC	-4.76111	0.019499	45S-rRNA
AGACGTGGTGACCCGCTGAATT	-4.76182	0.000609	28S-rRNA
CTTAGATCGATGTGGTGCTCCG	-4.78544	0.029645	45S-rRNA
GGCGGGCGGGGCCGG	-4.79342	0.0155	28S-rRNA
AGACGTGGTGACCCGCTGAATTTAAGCA	-4.79823	0.026956	28S-rRNA
AGATGTGGCGACCCGCTGAATT	-4.80949	0.015313	28S-rRNA
CGTAACTTCGGGATAAGGATTGGCTCTAAGGG	-4.82835	0.037933	28S-rRNA
GATGTGATTTCTGCCCAGTGCTCTGAATG	-4.83679	0.026389	28S-rRNA
CCGGGGCCGAGGGAG	-4.84868	7.09644184658536e-08	28S-rRNA

ACTTGGATGCGAGACCGCCTGGGAATACCGGGTGCTGTAGGCTT	-4.8491	0.034081	5S-rRNA
AGATTGATAGCTCTTTCTCGATTCCG	-4.85138	0.005209	18S-rRNA
CAGAGGAAACTCTGGTGGAGGTCCGTAGCGGTCCTGACGTG	-4.85426	0.005089	28S-rRNA
AGGCGTGGCGACCCGCTGAATTTAAGCA	-4.86278	0.011353	28S-rRNA
CAGGTGGGGAGTTTGACTGGGGCGGTA	-4.86416	0.011126	28S-rRNA
AGATCAGACGTGGCGACC	-4.8679	0.005933	28S-rRNA
AGCGGGGAAAGAAGACCCTGTTGAGCTTGACTCTAGTC	-4.87146	0.006218	28S-rRNA
TCCCGGGGCCGAGGGAGC	-4.87467	4.45567841586272e-07	28S-rRNA
CTCAGATCAGACGTGGCGACC	-4.8975	0.004056	28S-rRNA
CATACCACCCTGAACGCGCCCGATCTCGTCTGATC	-4.89792	0.04325	5S-rRNA
CGGCTCCGGGACGGCTGGG	-4.8984	8.3564578572891e-05	28S-rRNA
AGACGACCTGCTTCCGGGTCGGGGTTTCGTACG	-4.91814	0.041438	28S-rRNA
AGACGTGGCGACCCGCTAAATT	-4.91982	0.02707	28S-rRNA
AGACGTGGCGACCCGCTGAGTTTAAGCA	-4.92501	0.034641	28S-rRNA
CAGAGGAAACTCTGGTGGAGGTCCGTAGCGGTCCTGACG	-4.92891	0.031456	28S-rRNA
AGCCGGCTTAGAACTGGTGCGGAC	-4.93206	0.046182	28S-rRNA
GATCTCGTCTGATCTCGGAAGC	-4.93825	0.000425	5S-rRNA
AACGGCGAGTGAACAGGGAAGAGCC	-4.97381	0.00089	28S-rRNA
AGGACCCGAAAGATGGTGAACTATGCCTGGGCAGGGCGAAGC	-4.97805	0.003169	28S-rRNA
GGCGGGTGTTGACGCGGTGTGATTTC	-4.98483	0.045426	28S-rRNA
TCCCGGGGCCGAGGGAG	-4.99445	4.01046640430286e-11	28S-rRNA
ACCTGTGGGATTATGACTGAACGCCTCTAAGT	-5.00165	0.009076	28S-rRNA
CGTGCGTCGATGAAGAACGCAGCTAGCTG	-5.01831	0.046509	5.8S-rRNA
AGAGGAAACTCTGGTGGAGGTCCGTAG	-5.01868	0.02118	28S-rRNA
ATGGAAGTCGGAATC	-5.02438	0.0441	28S-rRNA
ACCCGTCGCCGCGCTCTCTCTCTC	-5.02988	0.024805	28S-rRNA
AAGACGGACCAGAGCGAAGGCATTTGCCAAGAATGTTTT	-5.03035	0.038447	18S-rRNA
AGAGGAAACCCTGGTGGAGGTCCGTAGCGGTCCTGACGTG	-5.03738	0.0427	28S-rRNA
AGGGGCGAAAGACTAATCGAACCATCTAGTAGCTGGTTC	-5.04226	0.020508	28S-rRNA
GGCGCGCGCCGCGGCTGGACG	-5.06804	0.049299	28S-rRNA
TGGGGTCGGCGGGGGACC	-5.07828	2.09129683946319e-05	28S-rRNA
ATCTGTGGGATTATGACTGAGCGCCTC	-5.09142	0.040351	28S-rRNA
CGTGGACGGTGTGAGGCCGGTAGCGGCCC	-5.09559	0.034431	28S-rRNA
ATGTTGAACGCACGTTCGTGTGGAACCTGGCGC	-5.09806	0.022365	28S-rRNA
CGGGGCCGAGGGAGC	-5.11584	3.71218952310282e-05	28S-rRNA
ACGGCGCGACCGCTCTC	-5.12613	0.037266	28S-rRNA
ATTGCGCCGCTAGAGGTGAAATTCTTGGA	-5.13241	0.002364	18S-rRNA
AGGTCTCCAAGGTGAACAGCCTCTGGCATGTTGGAACAATG	-5.13811	0.000114	28S-rRNA
CCCGGGGCCGAGGGA	-5.14529	0.025117	28S-rRNA
CTGTTGAGCTTGACTC	-5.14896	0.025457	28S-rRNA
AGACGTGGCGACCAGCTGAATT	-5.16812	0.023634	28S-rRNA
GTACGACTCTTAGCGGTGGATCACTCGGCT	-5.19193	9.79006137087332e-06	45S-rRNA
AAGCAGGGTCGGGCCTGGTTAG	-5.19298	0.000285	5S-rRNA
AGACGTGGCGACCCGCCGAATTTAAGCA	-5.19622	0.009123	28S-rRNA
GCCCGATCTCGTCTGA	-5.19991	0.0377	5S-rRNA
AGCCTCTGGCATGTTGGAACAATG	-5.20257	0.003634	28S-rRNA
CGCGGTTCCGGCGGCGTCCGGTGAGCTCTCGCTGGCCC	-5.20369	0.005436	28S-rRNA
ATGAGAGGTGTAGAATAAG	-5.21371	0.0079	28S-rRNA
CTCTTAGATCGATGTGGTGCT	-5.22208	0.005642	45S-rRNA
ACCCGTCGCCGCGCTCTCCCCC	-5.22802	0.004571	28S-rRNA
TCGGGTCTTCCCGGAGTCGGGTTGCTTGGGAATG	-5.22986	0.001309	28S-rRNA
TGGTGCGGACCAGGGGAATCCGACTGTTTAATTAAAACAAAG	-5.23579	0.029157	28S-rRNA
AGACGACCTGCTTCTGGGTCGGGGTTTCGTATGTAG	-5.24261	0.033772	28S-rRNA
ACCGGGTACTGTAGGCTT	-5.26309	0.046509	5S-rRNA
GGCTCCGGGACGGCTGGG	-5.29947	2.53097274455251e-06	28S-rRNA
CGTGCGTCGATGAGGAACGCAGCTAGCTGCGAGAAT	-5.3142	0.022734	5.8S-rRNA
CGGGGGGCGGGGCGGAC	-5.32915	1.40739278656071e-06	28S-rRNA
AGACGTGGCGACCCGCTGAATTTAAGCATAT	-5.34699	9.79006137087332e-06	28S-rRNA
AGACGTGGCGACCCGTTGAATT	-5.35721	0.036749	28S-rRNA
CGCTCCCCGGCGCCGCTCGTGGGGGGCCCAAGTCCTTCTGAT	-5.35956	8.36292796038969e-07	28S-rRNA
AGTCGCCGTGCCTAC	-5.36768	0.032068	18S-rRNA
ATTGCGCCGCTAGAGGTGAAATTCTTGGAC	-5.41056	0.015615	18S-rRNA
CGCGGTTCCGGCGGCGTCCGGTGAGCTCTCGC	-5.42001	0.019288	28S-rRNA
GGCGGGCGGGGCCGGG	-5.44964	0.003774	28S-rRNA
CCGGGGCCGAGGGAGC	-5.47544	1.6856949737359e-07	28S-rRNA
CGGGCGGGCGTGGGCG	-5.47979	0.039066	28S-rRNA
AGTAACGGCGAGTGAACAGGGAA	-5.48541	0.003778	28S-rRNA
CGGGCGGGCGGTCCGCCGCGAGGCGAG	-5.51819	2.9128312866596e-07	18S-rRNA
AGAGGAGACTCTGGTGGAGGTCCGTAGCGGTCCTGACGTG	-5.53264	0.023834	28S-rRNA
ACCCGTCGCCGCGCTCTCTCTCTCC	-5.54779	0.038571	28S-rRNA
CGGGGGGCGGGCGCCGGCGG	-5.57031	3.79994064589038e-06	18S-rRNA
GAAGGGCCCCGCCCGGGGGCCCGAGGTGGGATCCCGAGGCC	-5.57142	0.035893	28S-rRNA
AGACGTGGAGACCCGCTGAATT	-5.58801	0.025273	28S-rRNA
CGGGGGGCGGGGCGGACTG	-5.59943	0.002785	28S-rRNA
GGGTCGGGGGGCGGGG	-5.64092	0.002853	45S-rRNA
ACTCTGATCGTTTTTTCACTGACCCGG	-5.65245	0.003666	28S-rRNA
GGTGGCCATGGAAGTCGGAATCCGCTAAGGAGTGTGTAACAACT	-5.65349	0.000863	28S-rRNA
ACCCGTCGCCGCGCTCTCTCTCT	-5.66646	0.021745	28S-rRNA
ATCGATGTGGTGCTCC	-5.67179	0.001939	45S-rRNA
GCGCGCCGGGCCCGGGTCTTCCCG	-5.76099	0.048692	28S-rRNA
AGACGTGGCGACCCGCTGAATTTAAGCATATTAGT	-5.8151	0.0071	28S-rRNA
AGTGGGCCACTTTTGGTAAGCAGAACTGGCGCTGCGGGATGAAC	-5.83305	0.049782	28S-rRNA

GCAGCTAGGAATAATGGAA	-5.83338	0.049439	18S-rRNA
AGACGATCTGCTTCTGGGTCGGGGTTTCGTACGTAG	-5.84891	0.009076	28S-rRNA
GGGGCGGGGCGGACT	-5.87203	0.029645	28S-rRNA
AGGGGAATCCGACTGTTTAATTAAAACAAAG	-5.88483	3.10333001629589e-05	28S-rRNA
CGCGCCGGGGAGGTGGAGCACGAG	-5.88674	0.004167	28S-rRNA
CTCCACCCGCGCGTCGTTCCCCTCT	-5.8888	0.021984	28S-rRNA
AATACCGGGTGCTGTAGGC	-5.89277	0.010918	5S-rRNA
AAATATTCAAACGAGAACTTTGAAGGCCGAAGTGGAGAAGGGTTC	-5.89981	0.026966	28S-rRNA
CGCGACCTCAGATCAGATGTGGCGACCCGC	-5.90451	0.033409	28S-rRNA
AGACGTGGCAACCCGCTGAATT	-5.91084	0.006368	28S-rRNA
CCCGGGGCCGAGGGAGC	-5.9136	3.39445030830454e-07	28S-rRNA
GGCGGGTGTTGGCGCGATGTGAT	-5.92319	0.025273	28S-rRNA
CCCGTCGCCGCGCTCTCCCCC	-5.97899	0.012542	28S-rRNA
GAATCCCGGGGCCGAGGGAG	-5.98116	0.043529	28S-rRNA
TTGGAAAGCGTCGCGGTTCCGGCGGCGTCCGGTGAGCTC	-6.01453	0.030312	28S-rRNA
CGGGTCTTCCCGGAGTCGGGTTGCTTGGGAATG	-6.04032	1.78005794039731e-06	28S-rRNA
GGAAGTCGGAATCCGCTAAGGAGTGTGTAACAACTCA	-6.04511	0.041562	28S-rRNA
CCACCGCCCGTCCCCGCCC	-6.05203	0.020801	18S-rRNA
GGACGTGGCGACCCGCTGAATTTAAGCATAT	-6.075	0.005683	28S-rRNA
GTGCGTCGATGAAGAACGCAGTTAGCTGCGAGAAT	-6.0823	0.041651	5.8S-rRNA
AGACGGGGCGACCCGCTGAATT	-6.1047	0.045534	28S-rRNA
AGGGGGCGGGGCGGA	-6.11215	0.030564	28S-rRNA
CGGCGGCGGCGACTCTGGACGCGAGCCGGGCC	-6.11378	0.001842	28S-rRNA
CGGAACGGAACGGGACGGGAGCGGCCGCGGGTGCGCGTC	-6.13766	0.031456	28S-rRNA
CGCGACGAGTAGGGGGGCCGCTGCGGTGAGCCTTGAAGCC	-6.17571	0.044885	28S-rRNA
GGTGGATCACTCGGCTCG	-6.19861	3.85800976959798e-05	5.8S-rRNA
GGCGGGTGCTGACGCGATGTGATTTC	-6.20187	0.011692	28S-rRNA
CGTAGCGGTCCTGACGTGCAAATCGGTCGTCCGACCTGGG	-6.20939	0.005348	28S-rRNA
ACCATCTGCGGGATTATGACTGAACGCCTCTAAGT	-6.2123	0.014384	28S-rRNA
AGAGGAAGCTCTGGTGGAGGTCCGTAGCGGTCCTGACGTG	-6.21745	0.04651	28S-rRNA
CCGCCGGGCAGCTTCCGGGAAACCAAAGTCTTTGGGTTC	-6.2287	0.048734	18S-rRNA
ACTCCCCGGCGCCGCTCGTGGGGGGCCTAAGTCCTTCTGAT	-6.29385	0.022944	28S-rRNA
AGGAAACTCTGGTGG	-6.29556	0.047519	28S-rRNA
GTCCGGGGCTGCACGCGC	-6.29942	0.024241	18S-rRNA
AGTAACGGCGAGTGAACAGGGAAGAGCC	-6.33534	7.21692625529059e-06	28S-rRNA
GATCTCGTCTGATCTCGGAAGCTAAG	-6.3557	0.000187	5S-rRNA
CCTCCCGGCTCCGGCCGGGGGTCGGGCG	-6.39021	0.000846	18S-rRNA
AGGACCCGAAAGATGGTGAACTATGCCTGGG	-6.3909	1.64886286141753e-05	28S-rRNA
GGGGGCCGGCGGCGGCGGCGACTCTGGGCGCG	-6.40247	0.038583	28S-rRNA
GGGGCGCGAGGCGGGG	-6.41373	0.044493	28S-rRNA
ACGCGAGCCGGGCCC	-6.41463	0.04767	28S-rRNA
AAGCAGAACTGGCGCTGCGGGATGAACCGAACG	-6.46445	0.030564	28S-rRNA
CCCTGAACGCGCCCG	-6.47641	0.047091	5S-rRNA
GCGCACGGGGTCGGCGGCGAC	-6.48921	0.00712	28S-rRNA
CTCGGCGCCCCCTCGATGCTCTTAGCTGAGTGTC	-6.49467	0.004945	18S-rRNA
AGGGTCGGGCCTGGTTAGTACTTGGATGGGAGACCGCC	-6.53043	0.049445	5S-rRNA
CCGCGGCGGTGCGCCGCGACCGGCTCCGGGAC	-6.53127	0.028316	28S-rRNA
TCCGAAGGGACGGGCGATGGCCT	-6.54331	0.041544	28S-rRNA
CGACCTCAGATCAGACGTGGCGACCCG	-6.56423	0.026389	28S-rRNA
CACGGCCCTGGCGGAGC	-6.58569	0.026399	18S-rRNA
AGGGGCGAAAGACTAATCGAACCATCTAG	-6.59331	0.032068	28S-rRNA
CGGGGGCGGGGCGGA	-6.59877	0.03573	28S-rRNA
TCGGGGGGCGGGGCG	-6.60514	0.000291	28S-rRNA
CGCGACTTCAGATCAGACGTGGCGACCCG	-6.60747	0.047611	28S-rRNA
AGGCGTGGCGACCCGCTGAATTTAAGCATAT	-6.61708	0.006368	28S-rRNA
GGGGTCGGCGGGGGACC	-6.6626	3.39445030830454e-07	28S-rRNA
GCCGCCCGTGGGGGGCCCAAGTCCTTCTGAT	-6.6689	0.032797	28S-rRNA
AGACGTGGCGACCTGCTGAATTTAAGCA	-6.67111	0.006316	28S-rRNA
TCCTTCGATGTCGGC	-6.71241	0.04085	28S-rRNA
GGCCATACCACCCTGAACGCGCCCGATCTCGTCTGATCT	-6.719	0.049079	5S-rRNA
CCGCTCCCCGGCGCCGCTCGTGGGGGGCCCAAGTCCTTCTGAT	-6.7213	1.81985537940568e-06	28S-rRNA
CGCCGGGCCCGGGTCTTCCC	-6.72816	0.032068	28S-rRNA
AACGGCGAGTGAGCAGGGAAGAGCC	-6.74361	0.047853	28S-rRNA
CTCAGATCAGACGTGGCGACCCGCTGAATTTAAGCATAT	-6.74484	0.041562	28S-rRNA
GCCCGCCGCGCCGGGGAGGTGGAGCACGAG	-6.76478	0.005326	28S-rRNA
AGTAACGGCGAGTGAACAGGGAAGAGCCCAG	-6.78348	0.000471	28S-rRNA
ACTAATCCTAGCCCTAGCCCTAC	-6.79317	0.017992	16S-rRNA
AGTAACGGCGAGTGAACAGGGAAGAGC	-6.79928	0.006346	28S-rRNA
CGGGTGGGGTCCGCG	-6.81041	0.02497	28S-rRNA
ACTCCCCGGCGCCGCTCGTGGGGGGCCCAAGTCCTT	-6.85306	0.043395	28S-rRNA
CGGTGGATCACTCGGC	-6.85795	0.02309	5.8S-rRNA
GCCGGGGGGCGGGCGC	-6.86138	0.020774	18S-rRNA
AGACGCGGCGACCCGCTGAATTTAAGCATAT	-6.89771	0.007758	28S-rRNA
GGTGAGCCTTGAAGCCTAGGGCGCGGGCCCGGG	-6.90169	0.026952	28S-rRNA
GGGCGGGCGTGGGCG	-6.90549	0.029526	28S-rRNA
GCGGGGGTGGGCGGGC	-6.91537	0.01079	28S-rRNA
GGGGCGGGCGCCGGCGG	-6.92839	0.023241	18S-rRNA
AGGGGCCGGCGGCGGCGGCGACTCTGGACGC	-6.94861	0.011746	28S-rRNA
AACGGCGGGTGAACAGGGAAGAGCC	-6.95087	0.041236	28S-rRNA
ATGATTAAGAGGGACGGCCGGGGGCACTCG	-6.95496	0.010824	18S-rRNA
CTGGCGCTGTGGGAT	-6.98593	0.040774	28S-rRNA
AGTAACGGCGAGTGGACAGGGAAGAGCC	-7.00723	0.042921	28S-rRNA

ACGGCCATACCACCCTGAACGCGCCCGACCTCGTCTGATC	-7.02438	0.038583	5S-rRNA
ACTCTTAGATCGATGTGGTGCTCCGGAGT	-7.02866	0.028827	45S-rRNA
GGGCGGGCGGTCCGCCGCGAGGCG	-7.03043	0.007607	18S-rRNA
AGCAGCCGACTTAGGACTGGTGCGGAC	-7.04119	0.028254	28S-rRNA
GTGGAAGTCGGAATCCGCTAAGGAGTGTGTAACAACT	-7.04526	0.047663	28S-rRNA
AGACGTGGCGACCCGCTGAATTTAGGCATAT	-7.06392	0.011475	28S-rRNA
AGATCAGACGTGGCGACCCGCTGAATTTAAGCATATTAGT	-7.09815	4.82041453840159e-05	28S-rRNA
AGCCCTGAAAATGGATGGCGCTGGAGCGTCGGGCC	-7.10002	5.32983265725958e-05	28S-rRNA
ACCTGCCGAATCAAC	-7.11425	0.00912	28S-rRNA
ACGTTGAACGCACGTTCGTGTGGAATCTGGCGC	-7.12265	0.039801	28S-rRNA
AGATGTGGCGACCCGCTGAATTTAAGCA	-7.12622	0.046275	28S-rRNA
AGGTGTCCTAAGGCGAGCCCAGGGAGGACAGAAACCTCCCG	-7.13425	0.023517	28S-rRNA
AACCCGGTGAGCTCCCTCCCGGCTCCGGCCAGGGGT	-7.13959	0.029645	18S-rRNA
GACCGGCTCCGGGACGGC	-7.1564	0.04757	28S-rRNA
AGACGTGGCGACCCGCTGAATTTAAGCGTAT	-7.19421	0.012691	28S-rRNA
CACCTGGTTGATCCTGC	-7.20774	0.016354	45S-rRNA
AGACGACCTGCTTCTGGGTAGGGGTTTCGTACGTAG	-7.21119	0.025265	28S-rRNA
AACCCGGTGGGCTCCCTCCCGGCTCCGGCCGGGGG	-7.223	0.02575	18S-rRNA
TTTGAACGCACGTTCGTGTGGAACCTGGCGCTAAACCATTCG	-7.25362	0.030702	28S-rRNA
GCGGTTCCGGCGGCGTCCGGTGAGCTCTCG	-7.25435	0.018451	28S-rRNA
CGGCCGATCGAAAGGGAGTCGGGTTCAGATCCCCGAATC	-7.31804	0.041506	28S-rRNA
AGGGGAATCCGACTGTTTAATTAAAACAAAGCATCG	-7.32309	0.044268	28S-rRNA
ACTCCCCGGCGCCGCTCGTGGGGGGCCCAAATCCTTCTGAT	-7.33328	0.012963	28S-rRNA
CGGGGGGCGGGCGCCGGCG	-7.33793	0.001723	18S-rRNA
GGGGGCGGGCGCCGG	-7.35361	0.001575	18S-rRNA
GATCGATGTGGTGCTCCGGAGTTC	-7.37066	0.009519	45S-rRNA
TCCCCCGCCTCCCCTC	-7.39183	0.009382	28S-rRNA
GTGGCGACCCGCTGAATTTAAGCATAT	-7.45097	0.00221	28S-rRNA
AGACGTGGCGACCCGCTTAATT	-7.46701	0.009868	28S-rRNA
ACGTTCGTGGGGAACCTGG	-7.47336	0.008406	28S-rRNA
ACGTTGAACGCACGTTCGTGTGGAACCTGGAGC	-7.48625	0.012309	28S-rRNA
AGACGTGGCGACCCGCCGAATTTAAGCATAT	-7.54173	0.01145	28S-rRNA
ATCGCGAAGGCCCGCGGCGGGTGTTGA	-7.56939	0.011526	28S-rRNA
ACGTGTTAGGACCCGAAAGATGGTGAAC	-7.61142	0.004905	28S-rRNA
CCCGGGCGGGCGTGGGC	-7.63464	0.005683	28S-rRNA
AGCGGGGAAAGAAGACCCTGTTGAGCTTGACTC	-7.88855	0.000956	28S-rRNA
AGACGTGGCGGCCCGCTGAATTTAAGCATAT	-7.98683	0.004489	28S-rRNA
TCCCGGGCGGGCGTGGGC	-8.12469	0.001309	28S-rRNA
AAGGTGAACAGCCTCTGGCATGTTGGAACAATGTAGG	-8.31886	0.00089	28S-rRNA
CACGGCCCTGGCGGAGCGCTGAGAAGA	-8.36221	0.000272	18S-rRNA
CGGGGTCGGCGGCGAC	-8.40176	1.14834286852693e-05	28S-rRNA
GTAACTTCGGGATAAGGATTGGCTCTAAGGGCTGGGTCGGT	-8.40693	0.003142	28S-rRNA
GGTTCCGGCGGCGTCCGGTGAGCTCT	-8.48832	0.000274	28S-rRNA
CGAATCCCCGCCGCGCGTCGCGGCGTGGGAAATGTGGCG	-8.55178	0.002071	28S-rRNA
CGGGCGGGCGGTCCGCCGCGAGGCGAGC	-8.55354	1.13958320983031e-06	18S-rRNA
AGATCAGACGTGGCGACCCGCTGAATTTAAGCATAT	-8.75554	7.1931477355126e-05	28S-rRNA
CACCGCCGCGGCCCGGGTTCGTTTCCCGGTCAGGGAAC	6.921804	0.0118	mature-tRNA-Glu-TTC;mature-tRNA-Glu-CTC
AGGTCACGTCGGGGTCAC	5.843326	0.025491	mature-tRNA-Trp-CCA
GCCCGGCTAGCTCAGTCGGTAGAGCATGAGACTCTTAAT	5.746843	0.049153	mature-tRNA-Lys-CTT_5_end
ACCGCCGCGGCCCGGGTTCGTTTCCCGGTCAGGGAAC	5.108295	0.008506	mature-tRNA-Glu-CTC;mature-tRNA-Glu-TTC
TGGTCTAGTGGTTAGGATTCGGCG	3.807017	0.023135	mature-tRNA-Glu-CTC;mature-tRNA-Glu-TTC
GGTGAATATAGTTTACAAAAAACATTAGACTGTGAATCTGACAAC	3.152935	0.025548	mature-mt_tRNA-His-GTG_5_end
GGTGAATATTGTTTACAAAAAACATTAGACTGTGAATCTGACAA	2.719916	0.017946	mature-mt_tRNA-His-GTG_5_end
GGTGAATATAGTTTACAAAAAACATTAGACTGTGAATCTGACAA	2.520269	0.016774	mature-mt_tRNA-His-GTG_5_end
GGTGAATATAGTTTACAAAAAACATTAGACTGTGAATCTGAC	2.482346	0.049782	mature-mt_tRNA-His-GTG_5_end
ATCTCGGTGGAACCTCC	-2.51219	0.024804	mature-tRNA-Gln-CTG;mature-tRNA-Gln-TTG
TCCCACATGGTCTAGCGGTTAGGATTCCT	-2.8984	0.022277	mature-tRNA-Glu-TTC_5_end
CCAGGCGGCCCGGGTTCGACTCCCGGTGTGGGAA	-3.01027	0.006707	mature-tRNA-Glu-TTC_3_end
GCATGGGTGGTTCAGTGGTAGAATTCTC	-3.22032	0.040173	mature-tRNA-Gly-GCC_5_end
ACTCTGGACTCTGAATC	-3.54229	0.0288	mature-tRNA-Gln-CTG;mature-tRNA-Gln-TTG
GCATGGGTGGTTCAGTGGTAGAATTCT	-3.6291	0.007699	mature-tRNA-Gly-GCC_5_end
TCCGGCTCGAAGGACC	-3.76234	0.031456	mature-tRNA-Tyr-GTA
AGGGTTCAAGTCCCTGTTCGGGCGCC	-4.07211	0.028941	mature-tRNA-Lys-TTT
TCCCCGGCATCTCCACC	-4.20008	0.027071	mature-tRNA-Ala-TGC;mature-tRNA-Ala-CGC;mature-tRNA-Ala-AGC
AATGGTTAGCACTCTGGACTTTGAATC	-4.41884	0.032156	mature-tRNA-Gln-TTG;mature-tRNA-Gln-CTG
TCCCACATGGTCTAGCGGTTAGGATTCC	-4.54318	6.1376770263067e-05	mature-tRNA-Glu-TTC_5_end
GGCTCGTTGGTCTAGGGGTATGATTCT	-4.80311	0.024805	mature-tRNA-Pro-TGG_5_end;mature-tRNA-Pro-AGG_5_end;mature-tRNA-Pro-CGG_5_end
GGTTCCATGGTGTAATGGTTAGCACTCT	6.921804	0.0118	mature-tRNA-Gln-CTG_5_end;mature-tRNA-Gln-TTG_5_end
AGGCGGCCCGGGTTCGACTCCCGGTGTGGGAACC	5.843326	0.025491	mature-tRNA-Glu-TTC
CCCACATGGTCTAGCGGTTAGGATTCCTGGT	5.746843	0.049153	mature-tRNA-Glu-TTC
GCTTCTGTAGTGTAGTGGTTATCACGTTCGCCT	5.108295	0.008506	mature-tRNA-Val-CAC_5_end
ACCGGGCGGAAACAC	3.807017	0.023135	mature-tRNA-Val-CAC;mature-tRNA-Val-AAC;mature-tRNA-Gly-ACC
AGCGGTTAGGATTCCTGGTTTTCACC	3.152935	0.025548	mature-tRNA-Glu-TTC
CAATGAACACTCTGACC	2.719916	0.017946	mature-mt_tRNA-Val-TAC
AAGAAAGATTGCAAGAACT	2.520269	0.016774	mature-mt_tRNA-Ser-GCT_5_end
AATTAATATAACTGACTTCCAATTAGTAGATTCTGAATAAACC	2.482346	0.049782	mature-mt_tRNA-Gly-TCC
ACATGGTCTAGCGGTTAGGATTCCTGGTTTTCACC	-2.51219	0.024804	mature-tRNA-Glu-TTC
GCTTCTGTAGTGTAGTGGTTATCACGTTCGC	-2.8984	0.022277	mature-tRNA-Val-CAC_5_end
AGTGGTTAGGATTCGGCGCTCTCACCG	-3.01027	0.006707	mature-tRNA-Glu-CTC

Supplemental Table 4. Primer Sequences used for QPCR

Genes	Primer sequences	Genes	Primer sequences
hGAPDH	5'- GGCCTCCAAGGAGTAAGACC-3'<br>5'- AGGGGAGATTCAGTGTGGTG-3'	hIL-1β	5'- CGCCAATGACTCAGAGGAAGA-3'<br>5'- AGGGCGTCATTCAGGATGAA-3'
hMCP-1	5'- GATGCAATCAATGCCCCAGTC-3'<br>5'- TCCTTGGCCACAATGGTCTT-3'	hICAM-1	5'- ATGCCCAGACATCTGTGTCC-3'<br>5'- GGGGTCTCTATGCCCAACAA-3'
hIL-6	5'- GGTACATCCTCGACGGCATCT-3'<br>5'- GTGCCTCTTTGCTGCTTTCAC-3'	hVCAM-1	5'- TTTGACAGGCTGGAGATAGACT-3'<br>5'- TCAATGTGTAATTTAGCTCGGCA-3'
mGAPDH	5'-AACTTTGGCATTGTGGAAGG-3'<br>5'-GGATGCAGGGATGATGTTCT-3'	mALKBH3	5'-GCCAGGTAGCCATCCCTT-3'<br>5'-AGGGGAAGCTGGCTGAGT-3'
mDNMT2	5'-AGCCTGTGGCTTTCAGTATCA-3'<br>5'-TTGGCTGACTTTCTTCAACTACTGC-3'	mRNase L	5'-TAGGCGAACACATCAATGAGGA-3'<br>5'-CTGCCTCTGGAACGCTGAG-3'
mNSUN2	5'-TTGGACGACGACGAGGA-3'<br>5'-CCATTCTCCTCTAGCGAACATG-3'	mRNase T2	5'-ACTATGGCCCGATAGAGCAGA-3'<br>5'-ATTGGCTGCGATTAGAAGACC-3'
mMETTL3	5'-CAGTGCTACAGGATGACGGCTT-3'<br>5'-CCGTCCTAATGATGCGCTGCAG-3'	mDICER1	5'-GGTCCTTTCTTTGGACTGCCA-3'<br>5'-GCGATGAACGTCTTCCCTGA-3'
mADAR1	5'-TGAGCATAGCAAGTGGAGATACC-3'<br>5'-GCCGCCCTTTGAGAAACTCT-3'	mANG	5'-CATCCCAACAGGAAGGAAGGA-3'<br>5'-ACCTGGAGTCATCCTGAGCC-3'
mALKBH1	5'-AAGCGAAGACCCCGAAGTTTA-3'<br>5'-CAGTGGCGACTTGCTCTGA-3'	mBMP4	5'-TTCCTGGTAACCGAATGCTGA-3'<br>5'-CCTGAATCTCGGCGACTTTTT-3'
mSox17	5'-GATGCGGGATACGCCAGTG-3'<br>5'-CCACCACCTCGCCTTTCAC-3'	mID1	5'-CCTAGCTGTTCGCTGAAGGC-3'<br>5'-CTCCGACAGACCAAGTACCAC-3'
mIL-1β	5'-TTCAGGCAGGCAGTATCACTC-3'<br>5'-GAAGGTCCACGGGAAAGACAC-3'	mTMEM1000	5'-GACAATGGAGAAAAACCCCAAGA-3'<br>5'-GGTAGCAGGAGAGTTCGGC-3'
mE-Selectin	5'-ATGCCTCGCGCTTTCTCTC-3'<br>5'-GTAGTCCCGCTGACAGTATGC-3'	mTGFB1	5'-CCACCTGCAAGACCATCGAC-3'<br>5'-CTGGCGAGCCTTAGTTTGGAC-3'
mEndothelin	5'-GCACCGGAGCTGAGAATGG-3'<br>5'-GTGGCAGAAGTAGACACACTC-3'		
