# Supplementary figures and images for: Paternal hypercholesterolemia elicits sex-specific exacerbation of atherosclerosis in offspring
Source: JCI Insight. 2024 Sep 10;9(17):e179291. doi: 10.1172/jci.insight.179291 (PMC11385100; doi:10.1172/jci.insight.179291)

p-p65

65 kDA

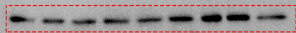

p65

65 kDA

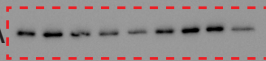

Actin

42 kDA

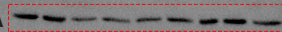

Supplement: Unedited blot and gel images [file jciinsight-9-179291-s013.pdf]
